# Supplementary material for: Evolution of multiple cell clones over a 29-year period of a CLL patient
Source: Nat Commun. 2016 Dec 16;7:13765. doi: 10.1038/ncomms13765 (PMC5171825; doi:10.1038/ncomms13765)
Supplement: Supplementary Information — Supplementary Figures and Supplementary Tables. [file ncomms13765-s1.pdf]

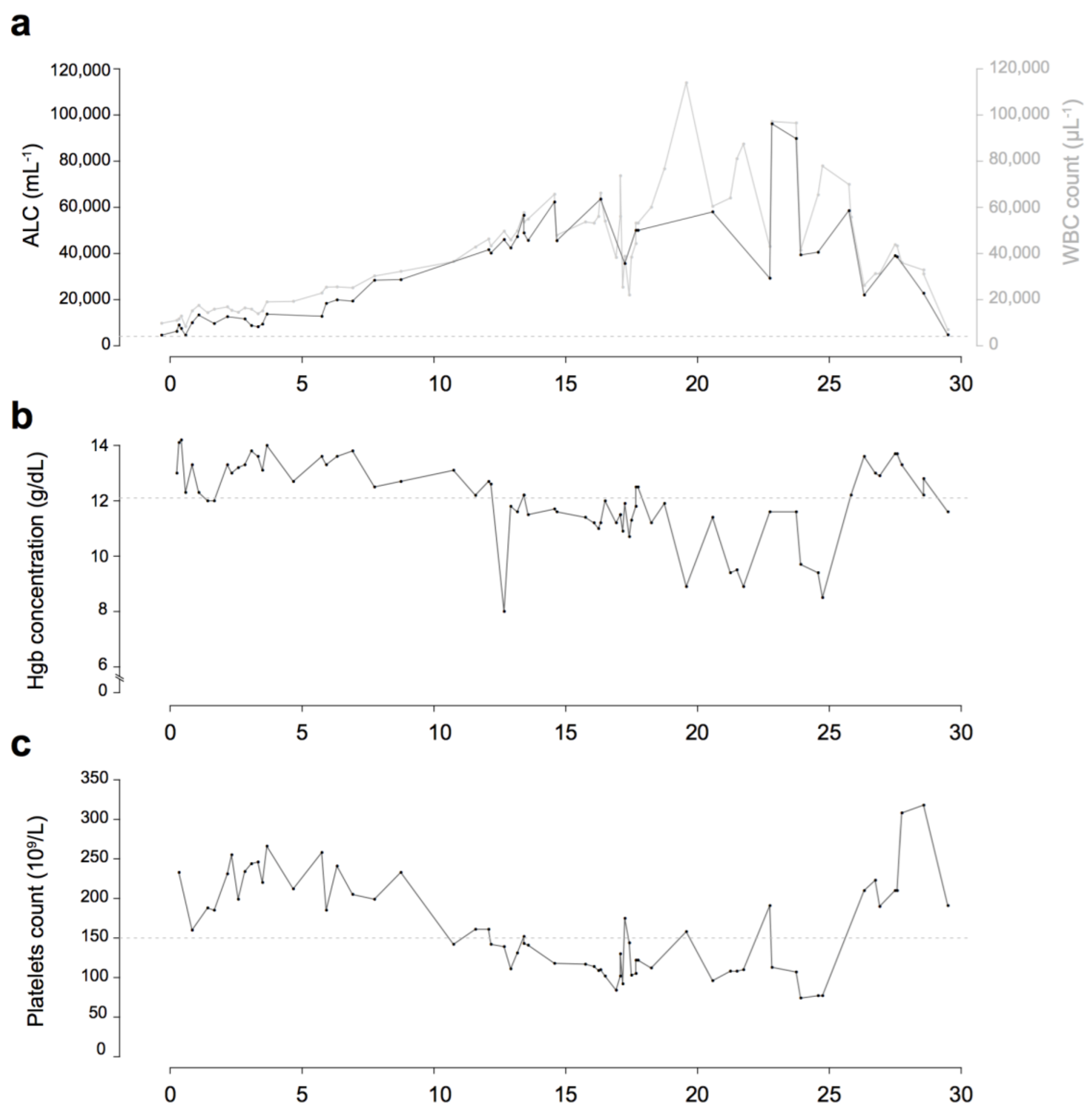

Supplementary Figure 1. Clinical information over 30-years of follow-up. **(a)** The absolute lymphocyte count (ALC, black) and white blood cell (WBC) count (grey) data from year -1 (1 year before diagnosis) to year 29 after diagnosis. The dashed grey line represents the normal upper limit of ALC (4,000 mL^{-1}). **(b)** The hemoglobin (Hgb) concentration data from year 0 to year 29. The dashed grey line represents the normal lower limit (12.1 g/dL). **(c)** The platelets count data from year 0 to year 29. The dashed grey line represents the normal lower limit (150 x 10^9/L).

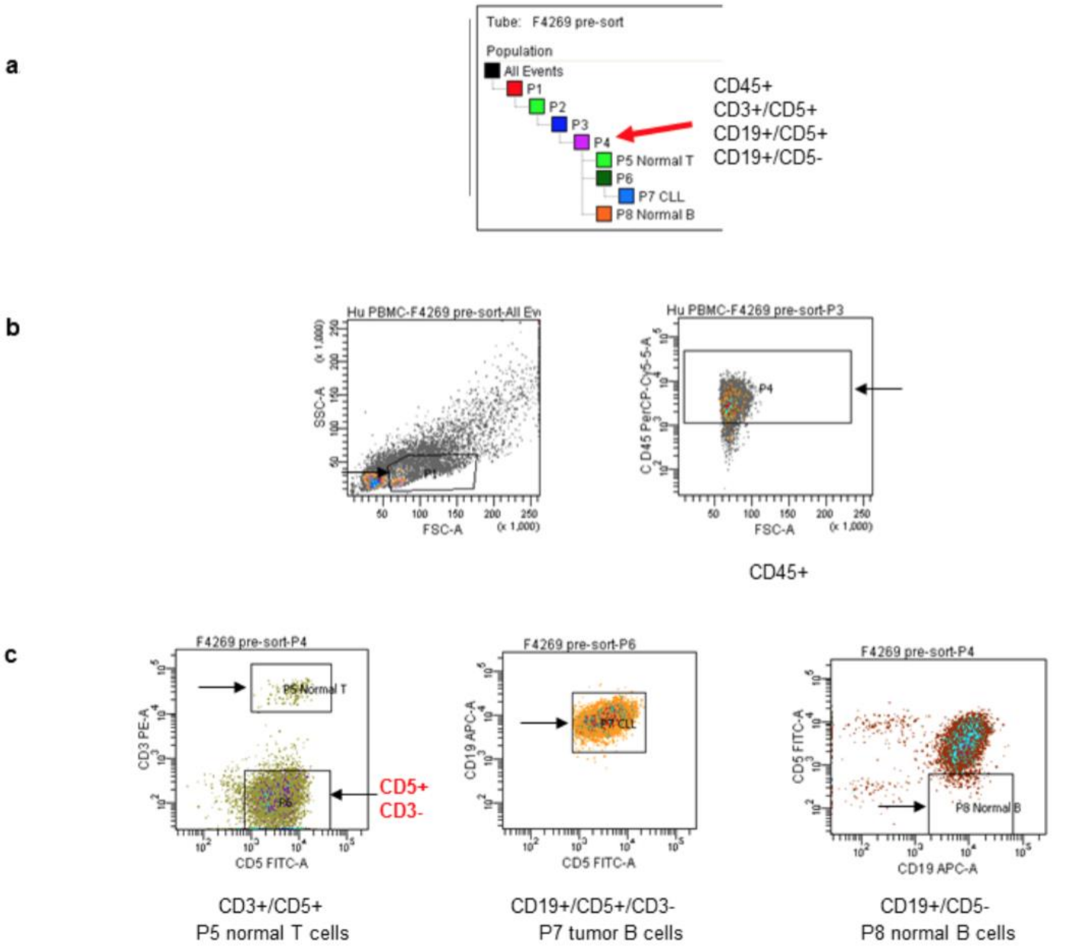

Supplementary Figure 2. FACS cell sorting. Typical plots from cell sorting are shown with A) representing the overall schema, B) gating of all viable cells positive for CD45, and C) the gating for normal T cells, tumor B cells and normal B cells. Normal B cell numbers were too small to use in analyses.

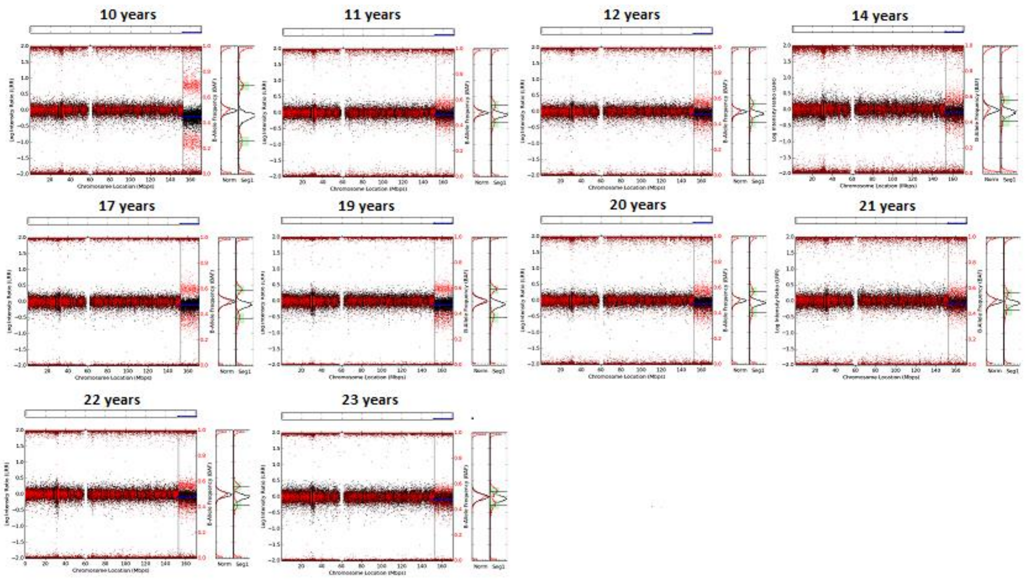

Supplementary Figure 3. CNV profiles of chr6 from year 10 to year 23 after diagnosis based on SNP microarray.

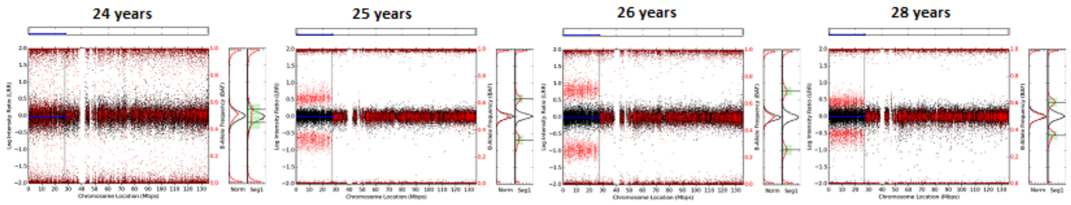

Supplementary Figure 4. CNV profiles of chr10 from year 24 to year 28 after diagnosis based on SNP microarray.

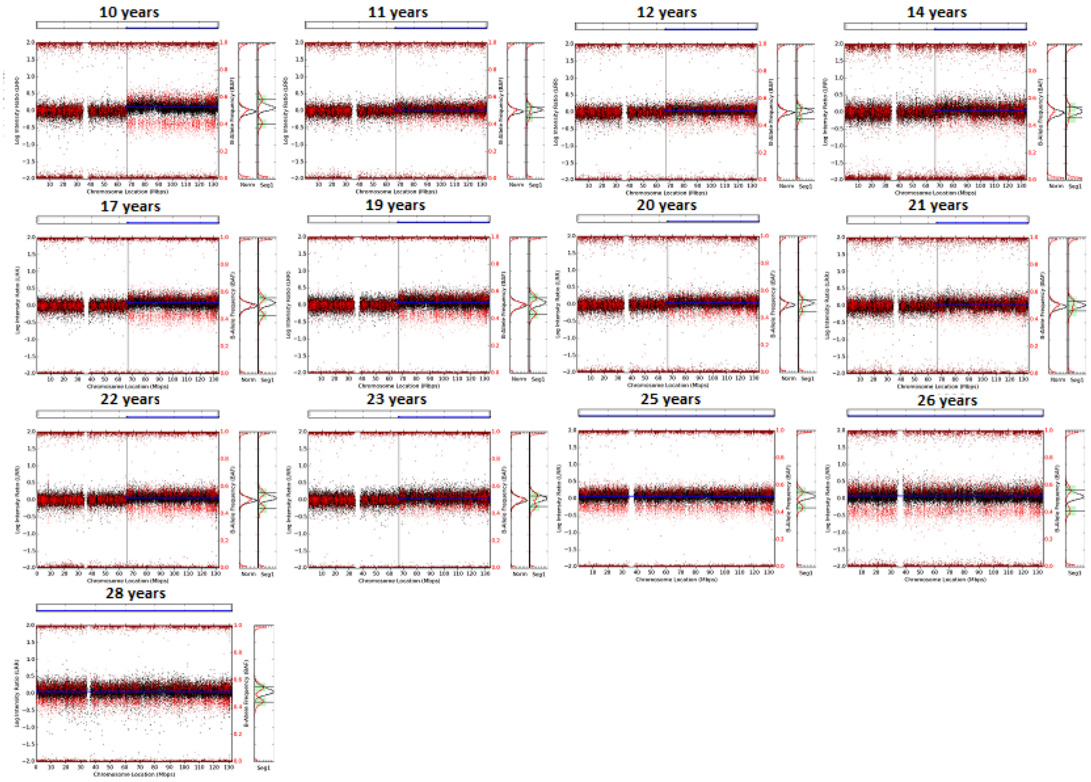

Supplementary Figure 5. CNV profiles of chr12 from year 10 to year 28 after diagnosis based on SNP microarray.

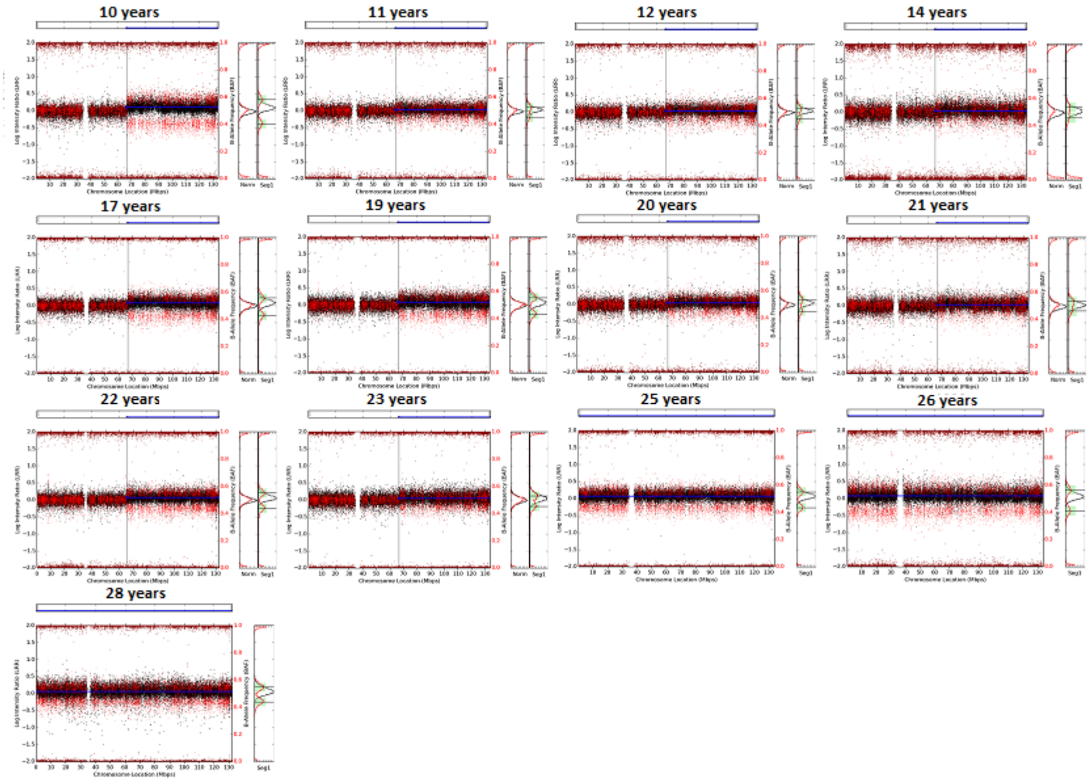

Supplementary Figure 6. CNV profiles of chromosome 13 from year 10 to year 28 after diagnosis based on SNP microarray.

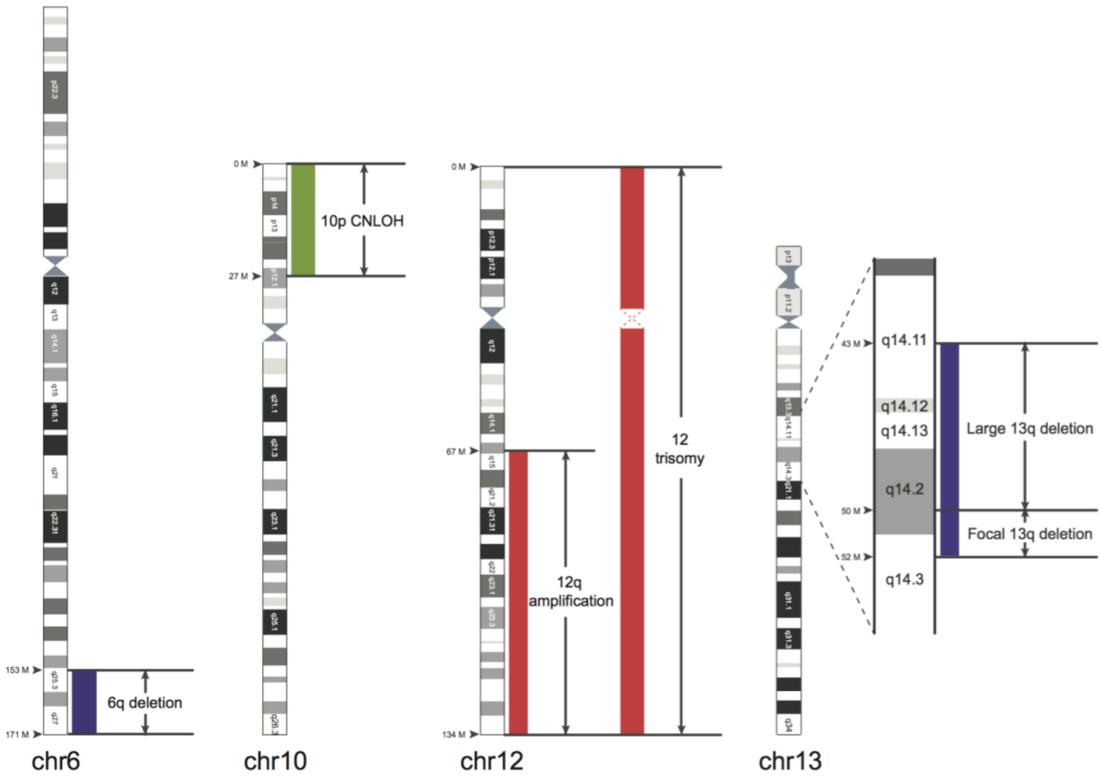

Supplementary Figure 7. Map of the detected CNVs. Red indicates amplification, blue deletion, and green CNLOH.

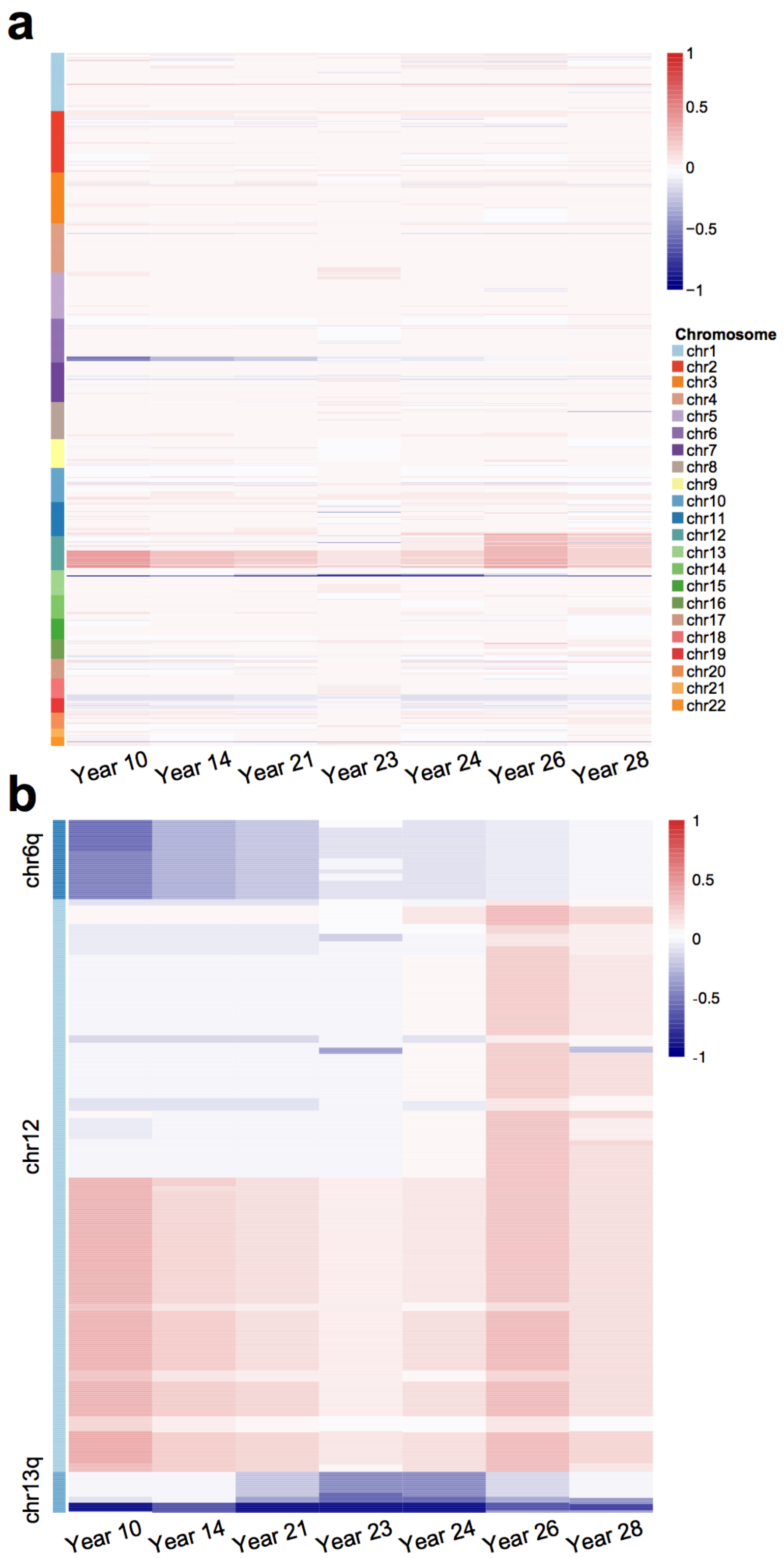

Supplementary Figure 8. CNV profiles of 7 time points based on whole genome sequencing. **(a)**. The CNV profiles of all chromosomes. **(b)**. The CNV profiles of 6q, chr12 and 13q. Red-amplification, blue-deletion.

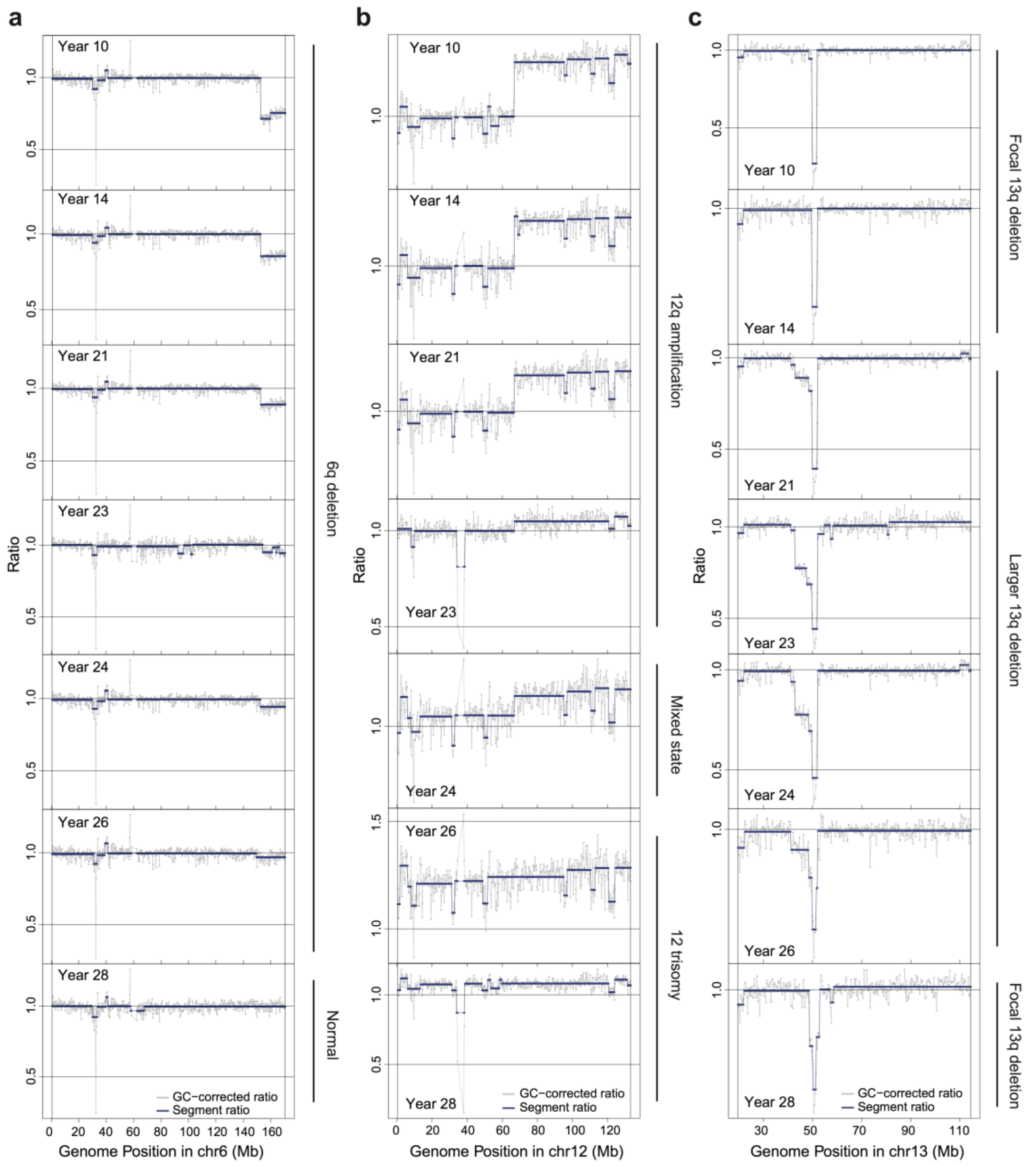

Supplementary Figure 9. The detailed segment ratios of chr6, chr12 and chr13 in the 7 WGS samples. **(a)** The detailed segment ratios of chr6. **(b)** The detailed segment ratios of chr12. **(c)** The detailed segment ratios of chr13.

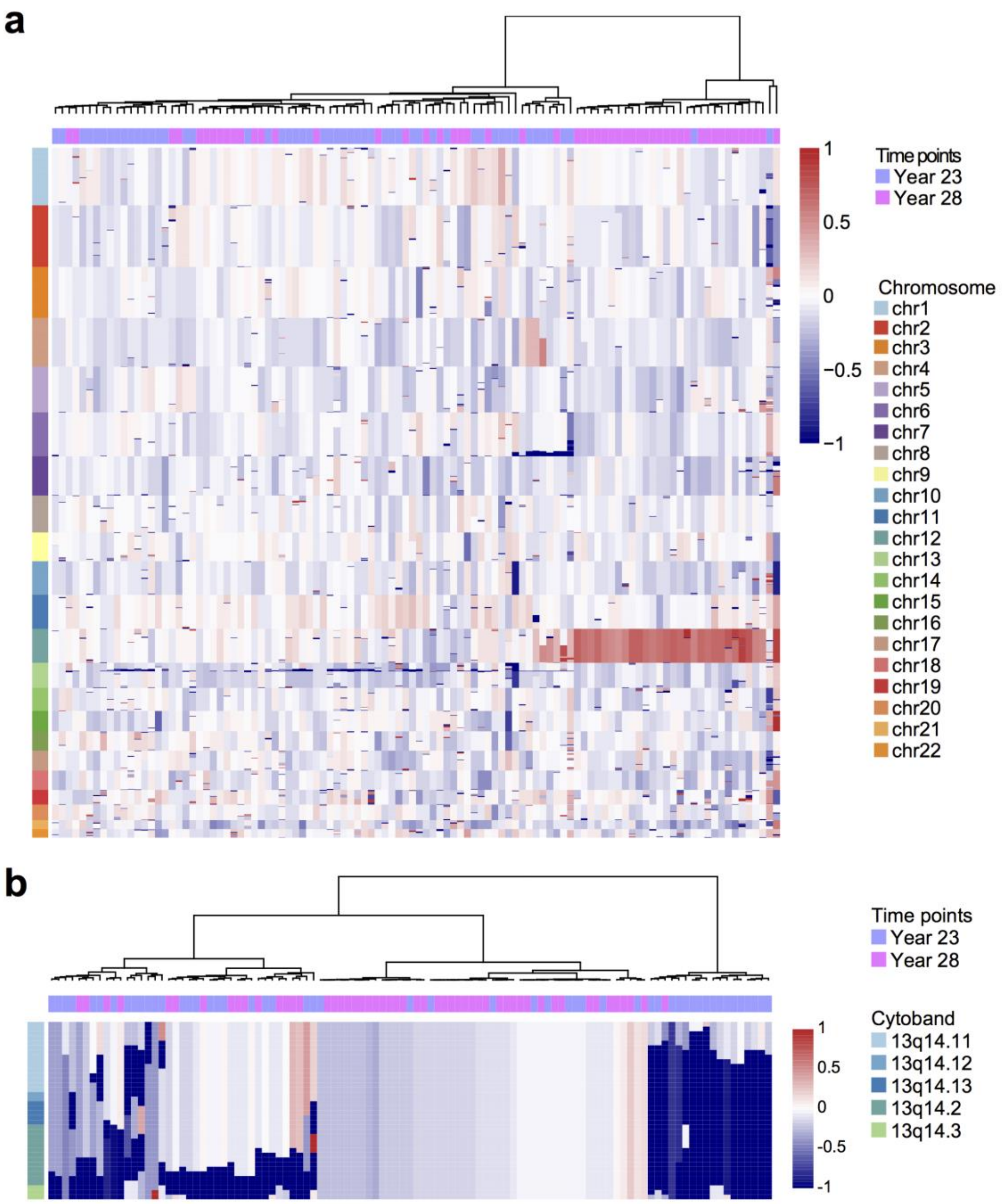

Supplementary Figure 10. The CNV profiles of single cells from year 23 and year 28 after diagnosis. **(a)** The whole genome CNV profiles of all single cells. **(b)** The CNV profiles of 13q14 in all single cells. Red indicates amplification, and blue indicates deletion.

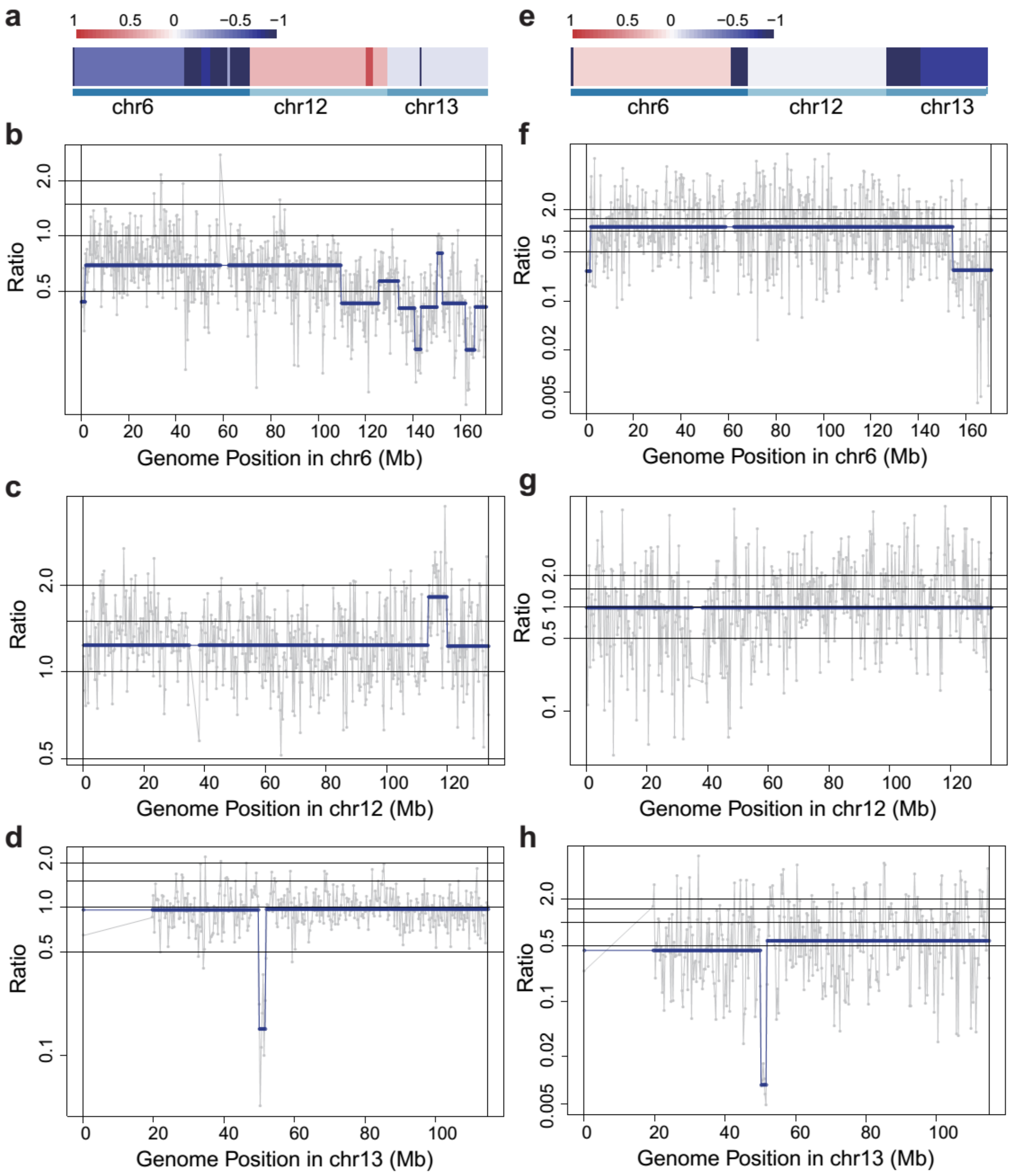

Supplementary Figure 11. The CNV profiles of 2 cells removed from the 3 main CNV analysis. **(a)** The CNV profiles of the cell with 6q deletion and chr12 trisomy. **(b-d)** The detailed segment ratios of chr6, chr12 and chr13 of the cell in **(a)**. **(e)** The CNV profiles of the cell with 6q deletion and large 13q deletion. **(f-h)** The detailed segment ratios of chr6, chr12 and chr13 of the cell in **(e)**.

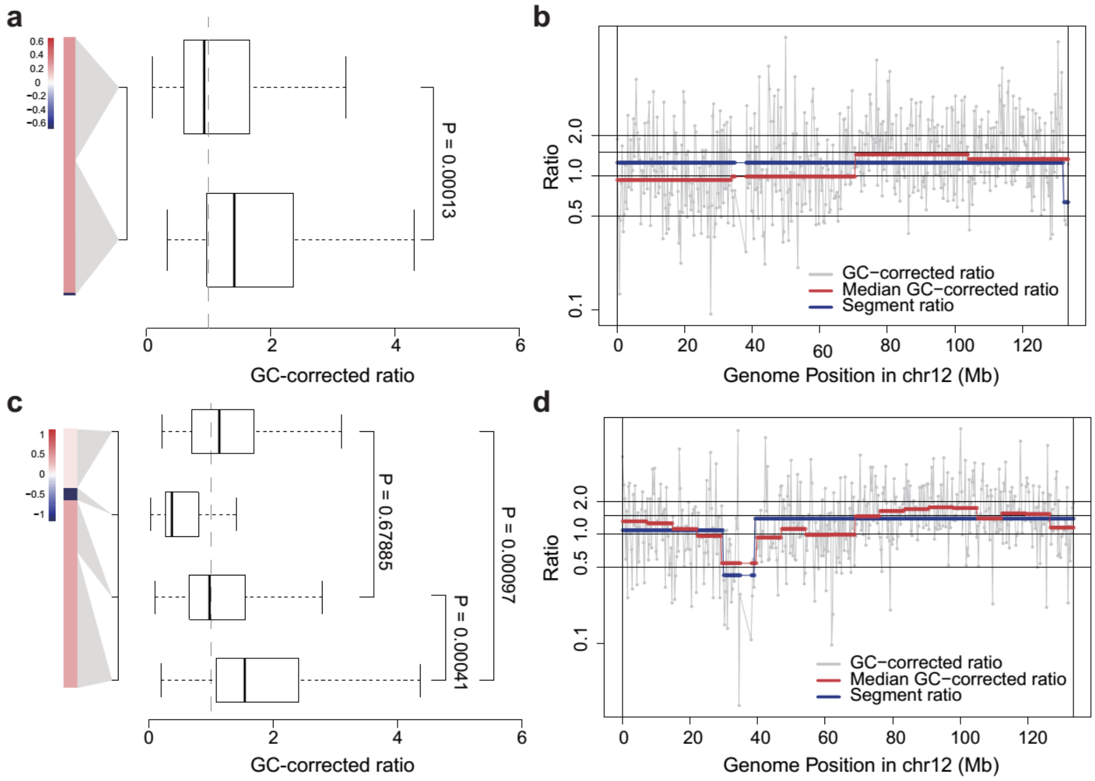

Supplementary Figure 12. False positive amplification in chr12 in 2 single cells. **(a)** The difference of the GC-corrected ratio between the false positive and true amplification region (Student t-test) of the cell with whole chr12 amplification. **(b)** The comparison of the GC-corrected ratio and the segment ratio of the cell in **(a)**. **(c)** The difference of the GC-corrected ratio between the false positive and true amplification region (Student t test) of the cell with larger chr12 amplification. **(d)** The comparison of the GC-corrected ratio and the segment ratio of the cell in **(c)**.

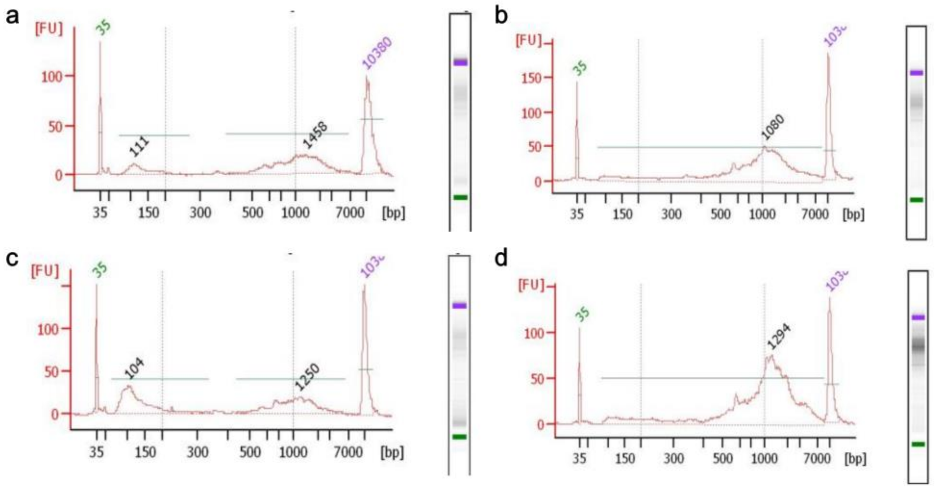

Supplementary Figure 13. The typical Agilent 2100 bioanalyzer detection results of 4 random selected cDNA products of amplified single-cell RNA.

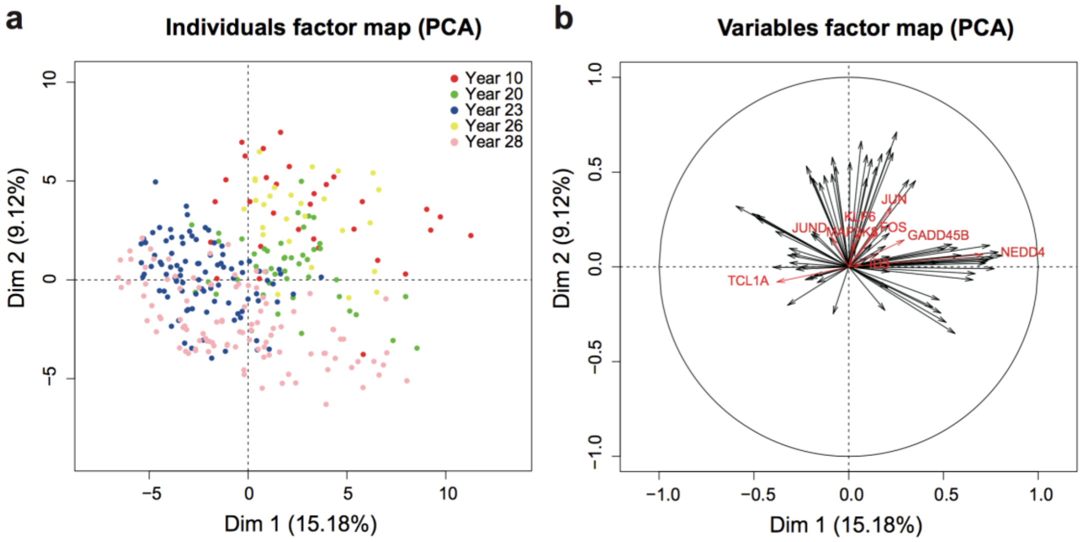

Supplementary Figure 14. The principle component analysis of single-cell RNA-sequences in five samples from 10-28 years after diagnosis. **(a)**. The PCA results of all the qualified single cells. **(b)**. The variable factors of 80 selected genes for the hierarchical analysis based on the PCA analysis. Red indicates the genes which may play a role in tumor progression.

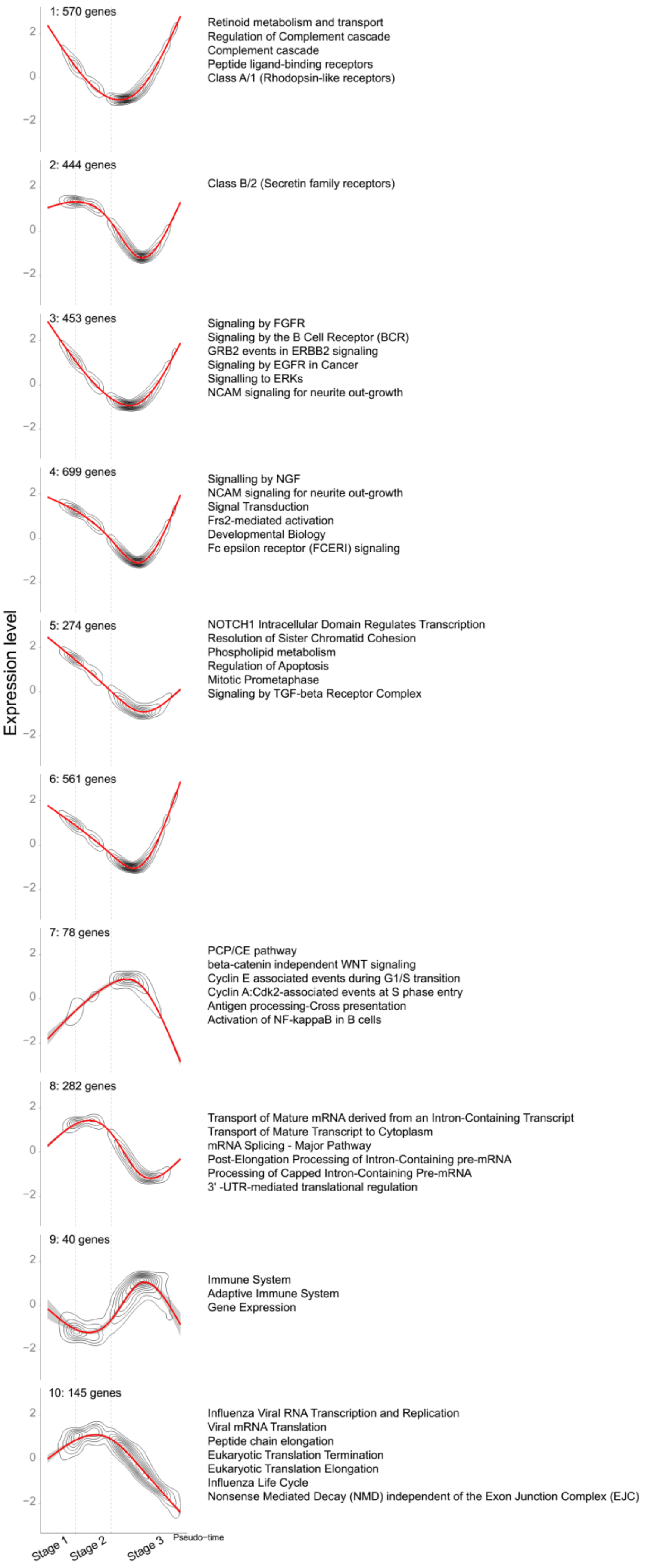
Expression level
1: 570 genes
Retinoid metabolism and transport
Regulation of Complement cascade
Complement cascade
Peptide ligand-binding receptors
Class A/1 (Rhodopsin-like receptors)
2: 444 genes
Class B/2 (Secretin family receptors)
3: 453 genes
Signaling by FGFR
Signaling by the B Cell Receptor (BCR)
GRB2 events in ERBB2 signaling
Signaling by EGFR in Cancer
Signalling to ERKs
NCAM signaling for neurite out-growth
4: 699 genes
Signalling by NGF
NCAM signaling for neurite out-growth
Signal Transduction
Frs2-mediated activation
Developmental Biology
Fc epsilon receptor (FCERI) signaling
5: 274 genes
NOTCH1 Intracellular Domain Regulates Transcription
Resolution of Sister Chromatid Cohesion
Phospholipid metabolism
Regulation of Apoptosis
Mitotic Prometaphase
Signaling by TGF-beta Receptor Complex
6: 561 genes
7: 78 genes
PCP/CE pathway
beta-catenin independent WNT signaling
Cyclin E associated events during G1/S transition
Cyclin A:Cdk2-associated events at S phase entry
Antigen processing-Cross presentation
Activation of NF-kappaB in B cells
8: 282 genes
Transport of Mature mRNA derived from an Intron-Containing Transcript
Transport of Mature Transcript to Cytoplasm
mRNA Splicing - Major Pathway
Post-Elongation Processing of Intron-Containing pre-mRNA
Processing of Capped Intron-Containing Pre-mRNA
3' -UTR-mediated translational regulation
9: 40 genes
Immune System
Adaptive Immune System
Gene Expression
10: 145 genes
Influenza Viral RNA Transcription and Replication
Viral mRNA Translation
Peptide chain elongation
Eukaryotic Translation Termination
Eukaryotic Translation Elongation
Influenza Life Cycle
Nonsense Mediated Decay (NMD) independent of the Exon Junction Complex (EJC)
Stage 1
Stage 2
Stage 3
Pseudo−time

Supplementary Figure 15. The clustering of monocle and enriched pathways of each cluster. Only parts of the significant enriched pathways ($P < 0.05$) were selected, and the entire results of pathways enrichment are in **Supplementary Table 6**.

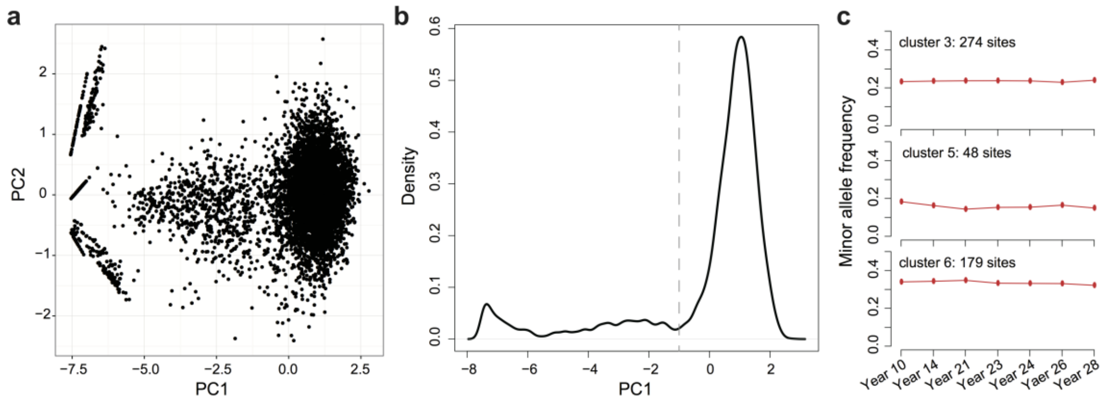

Supplementary Figure 16. The filtering of possible germline SNPs. **(a)** PCA of all the qualified SNPs in 7 WGS samples after depth filtering. **(b)** The distribution of the PC1 values of all the qualified SNPs. The dash grey line indicates the PC1 = -1. **(c)** The 3 clusters removed from the MAF analysis.

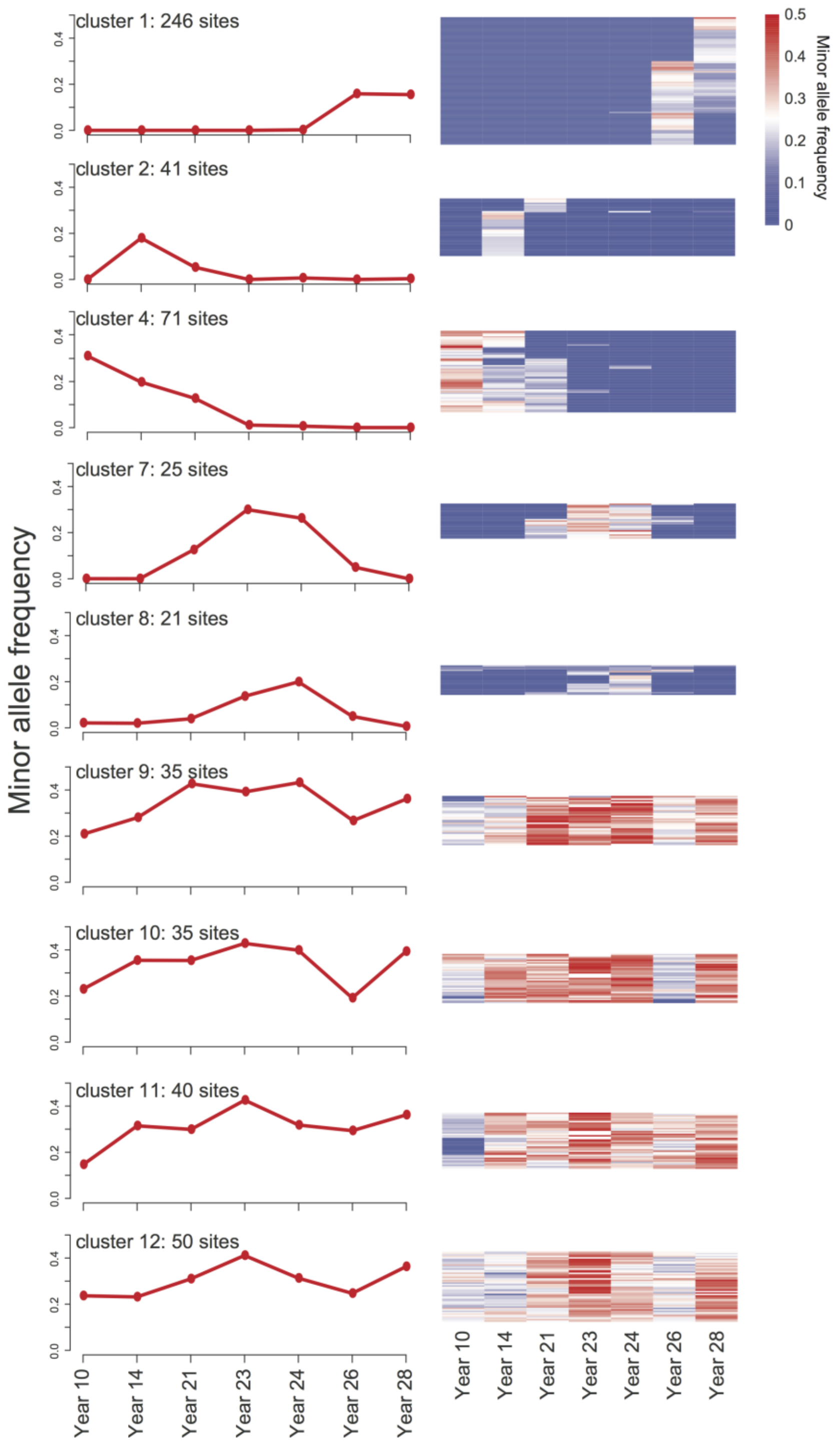

Supplementary Figure 17. The minor allele frequency changes of SNPs from year 10 to year 28 after diagnosis based on whole genome sequencing. The left panel is the MAF pattern using the SOTA method. The right panel is the MAF heatmap of the same sites form the left panel.

Supplementary Table 1 Whole genome sequencing of 7 CLL bulk samples

Sample_ID	Total_Raw_Reads	Total_Mapped_Reads	Uniquely_Mapped_Reads	Mapping_Ratio	Depth	Coverage
Year_10	763,262,252	761,072,367	726,575,091	99.71%	33.21	97.29%
Year_14	720,449,793	718,617,391	684,318,934	99.75%	31.18	97.29%
Year_21	743,416,437	741,338,443	706,921,082	99.72%	32.43	97.29%
Year_23	672,678,471	670,504,166	645,324,062	99.68%	29.83	97.29%
Year_24	742,249,265	740,098,058	704,779,189	99.71%	32.30	97.29%
Year_28	972,996,888	970,077,897	928,104,501	99.70%	42.12	97.29%
Year_28	975,216,411	971,950,779	936,530,795	99.67%	42.25	97.29%

Supplementary Table 2 Light-coverage whole genome sequencing of single cells from 1995 and 2000

Sample_ID	Total_Raw_Reads	Total_Mapped_Reads	Uniquely_Mapped_Reads	Mapping_Ratio	Depth	Coverage
FA-00-1	4,880,646	4,352,370	4,352,370	89.18%	0.13	8.48%
FA-00-101	5,618,637	4,953,585	4,953,585	88.16%	0.15	9.50%
FA-00-102	6,687,556	5,902,972	5,902,972	88.27%	0.18	12.70%
FA-00-103	5,996,990	5,285,985	5,285,985	88.14%	0.16	12.29%
FA-00-104	6,016,013	5,284,337	5,284,337	87.84%	0.16	11.58%
FA-00-105	4,579,686	4,023,293	4,023,293	87.85%	0.12	8.43%
FA-00-107	4,693,787	4,136,609	4,136,609	88.13%	0.13	8.70%
FA-00-108	4,420,269	3,876,384	3,876,384	87.70%	0.12	8.55%
FA-00-16	4,573,877	4,070,774	4,070,774	89.00%	0.13	8.80%
FA-00-19	4,196,608	3,752,958	3,752,958	89.43%	0.12	8.16%
FA-00-20	5,051,755	4,504,961	4,504,961	89.18%	0.14	9.18%
FA-00-25	4,407,329	3,918,645	3,918,645	88.91%	0.12	9.05%
FA-00-28	766,229	766,229	766,229	100.00%	0.02	1.60%
FA-00-29	4,600,911	4,089,339	4,089,339	88.88%	0.13	9.24%
FA-00-3	4,533,015	4,018,675	4,018,675	88.65%	0.12	6.28%
FA-00-31	5,127,462	4,559,775	4,559,775	88.93%	0.14	8.45%
FA-00-33	4,370,513	3,859,114	3,859,114	88.30%	0.12	7.49%
FA-00-34	4,900,398	4,335,787	4,335,787	88.48%	0.13	9.00%
FA-00-36	4,794,808	4,259,153	4,259,153	88.83%	0.13	7.94%
FA-00-38	4,475,416	3,962,120	3,962,120	88.53%	0.12	7.16%
FA-00-4	4,506,586	3,992,044	3,992,044	88.58%	0.12	7.63%
FA-00-42	4,360,154	3,881,477	3,881,477	89.02%	0.12	8.22%
FA-00-43	5,061,369	4,511,448	4,511,448	89.13%	0.14	9.26%
FA-00-45	4,270,733	3,754,547	3,754,547	87.91%	0.12	8.10%
FA-00-46	4,283,566	3,782,553	3,782,553	88.30%	0.12	9.50%
FA-00-47	4,954,105	4,365,982	4,365,982	88.13%	0.13	9.01%
FA-00-50	4,785,370	4,203,879	4,203,879	87.85%	0.13	9.00%
FA-00-51	5,501,411	4,860,751	4,860,751	88.35%	0.15	10.68%

FA-00-52	5,037,927	4,438,805	4,438,805	88.11%	0.14	9.38%
FA-00-53	5,218,838	4,606,395	4,606,395	88.26%	0.14	9.70%
FA-00-54	4,492,678	3,970,463	3,970,463	88.38%	0.12	9.31%
FA-00-55	4,486,415	3,949,813	3,949,813	88.04%	0.12	9.36%
FA-00-57	4,362,166	3,861,013	3,861,013	88.51%	0.12	9.60%
FA-00-59	4,349,893	3,828,885	3,828,885	88.02%	0.12	8.52%
FA-00-60	4,372,478	3,853,560	3,853,560	88.13%	0.12	8.70%
FA-00-61	4,409,898	3,902,986	3,902,986	88.51%	0.12	9.13%
FA-00-62	5,011,247	4,421,803	4,421,803	88.24%	0.14	9.03%
FA-00-64	5,706,376	5,053,528	5,053,528	88.56%	0.16	11.29%
FA-00-65	4,211,908	3,723,022	3,723,022	88.39%	0.11	9.17%
FA-00-67	3,883,006	3,430,358	3,430,358	88.34%	0.11	7.86%
FA-00-68	4,668,945	4,112,032	4,112,032	88.07%	0.13	7.04%
FA-00-69	4,330,275	3,810,785	3,810,785	88.00%	0.12	8.67%
FA-00-7	4,672,979	4,157,573	4,157,573	88.97%	0.13	8.71%
FA-00-70	4,103,044	3,622,282	3,622,282	88.28%	0.11	9.06%
FA-00-71	5,877,376	5,191,605	5,191,605	88.33%	0.16	11.74%
FA-00-72	4,720,247	4,179,129	4,179,129	88.54%	0.13	10.15%
FA-00-74	4,338,554	3,843,118	3,843,118	88.58%	0.12	9.24%
FA-00-76	5,160,014	4,551,706	4,551,706	88.21%	0.14	9.74%
FA-00-77	5,177,235	4,571,839	4,571,839	88.31%	0.14	10.43%
FA-00-78	5,391,797	4,752,220	4,752,220	88.14%	0.15	10.38%
FA-00-79	6,091,028	5,367,992	5,367,992	88.13%	0.17	11.90%
FA-00-80	4,100,598	3,618,095	3,618,095	88.23%	0.11	8.88%
FA-00-82	4,440,875	3,908,902	3,908,902	88.02%	0.12	8.61%
FA-00-84	5,761,687	5,086,547	5,086,547	88.28%	0.16	11.10%
FA-00-85	5,800,620	5,111,273	5,111,273	88.12%	0.16	10.17%
FA-00-86	5,516,315	4,863,455	4,863,455	88.16%	0.15	9.91%
FA-00-88	5,321,739	4,698,382	4,698,382	88.29%	0.15	10.92%
FA-00-92	6,156,000	5,056,901	5,056,901	82.15%	0.16	10.25%
FA-00-93	6,271,194	5,503,772	5,503,772	87.76%	0.17	11.39%
FA-00-94	6,642,946	5,881,425	5,881,425	88.54%	0.18	10.98%

FA-00-96	5,233,511	4,616,662	4,616,662	88.21%	0.14	10.46%
FA-00-99	5,049,381	4,463,514	4,463,514	88.40%	0.14	10.07%
FG-95-1	2,984,477	2,611,938	2,611,938	87.52%	0.08	7.13%
FG-95-10	4,043,962	3,560,688	3,560,688	88.05%	0.00	0.25%
FG-95-11	4,345,944	3,797,620	3,797,620	87.38%	0.12	9.86%
FG-95-13	3,773,210	3,300,467	3,300,467	87.47%	0.10	8.86%
FG-95-14	4,468,956	3,900,205	3,900,205	87.27%	0.12	10.28%
FG-95-15	3,666,253	3,205,429	3,205,429	87.43%	0.10	8.70%
FG-95-16	3,729,272	3,268,316	3,268,316	87.64%	0.10	8.89%
FG-95-17	4,184,100	3,671,854	3,671,854	87.76%	0.11	10.12%
FG-95-18	4,248,961	3,730,999	3,730,999	87.81%	0.12	10.38%
FG-95-19	4,063,241	3,556,877	3,556,877	87.54%	0.11	9.40%
FG-95-2	3,477,954	3,043,859	3,043,859	87.52%	0.09	8.22%
FG-95-20	4,227,993	3,701,063	3,701,063	87.54%	0.11	9.78%
FG-95-21	4,039,983	3,532,087	3,532,087	87.43%	0.11	9.72%
FG-95-22	3,704,915	3,240,122	3,240,122	87.45%	0.10	8.59%
FG-95-23	3,678,096	3,214,409	3,214,409	87.39%	0.10	8.55%
FG-95-26	4,104,753	3,590,791	3,590,791	87.48%	0.11	9.29%
FG-95-27	3,569,634	3,154,781	3,154,781	88.38%	0.10	8.63%
FG-95-28	3,904,768	3,422,088	3,422,088	87.64%	0.11	9.29%
FG-95-3	4,427,740	3,872,356	3,872,356	87.46%	0.12	10.10%
FG-95-32	3,830,468	3,386,749	3,386,749	88.42%	0.10	7.83%
FG-95-34	4,427,848	3,921,457	3,921,457	88.56%	0.12	8.49%
FG-95-35	4,229,861	3,751,036	3,751,036	88.68%	0.12	7.79%
FG-95-4	3,802,757	3,318,862	3,318,862	87.28%	0.10	8.42%
FG-95-42	4,432,836	3,923,615	3,923,615	88.51%	0.12	8.90%
FG-95-46	3,548,963	3,144,737	3,144,737	88.61%	0.10	7.51%
FG-95-49	4,170,947	3,702,730	3,702,730	88.77%	0.11	8.63%
FG-95-5	4,249,966	3,722,513	3,722,513	87.59%	0.11	9.71%
FG-95-51	4,700,888	4,166,530	4,166,530	88.63%	0.13	8.97%
FG-95-53	4,296,290	3,803,043	3,803,043	88.52%	0.12	8.48%
FG-95-55	4,584,340	4,071,614	4,071,614	88.82%	0.13	9.22%

FG-95-56	4,138,890	3,674,982	3,674,982	88.79%	0.11	8.50%
FG-95-57	4,297,494	3,815,105	3,815,105	88.78%	0.12	8.86%
FG-95-58	4,614,681	4,102,584	4,102,584	88.90%	0.13	7.72%
FG-95-6	3,337,781	2,923,067	2,923,067	87.58%	0.09	7.76%
FG-95-60	4,090,621	3,623,711	3,623,711	88.59%	0.11	8.90%
FG-95-64	3,988,909	3,529,565	3,529,565	88.48%	0.11	7.91%
FG-95-67	3,713,302	3,282,393	3,282,393	88.40%	0.10	7.42%
FG-95-68	5,781,770	5,104,528	5,104,528	88.29%	0.16	10.54%
FG-95-7	3,542,386	3,104,383	3,104,383	87.64%	0.10	8.68%
FG-95-70	5,249,616	4,645,976	4,645,976	88.50%	0.14	9.76%
FG-95-74	5,500,799	4,852,737	4,852,737	88.22%	0.15	10.83%
FG-95-75	5,326,727	4,675,481	4,675,481	87.77%	0.14	9.77%
FG-95-76	5,006,100	4,394,686	4,394,686	87.79%	0.14	8.36%
FG-95-79	4,950,643	4,353,539	4,353,539	87.94%	0.13	8.74%
FG-95-8	3,846,393	3,373,106	3,373,106	87.70%	0.10	9.48%
FG-95-87	4,843,872	4,262,462	4,262,462	88.00%	0.13	8.48%
FG-95-9	4,171,632	3,657,244	3,657,244	87.67%	0.11	9.95%
FG-95-90	5,263,771	4,647,658	4,647,658	88.30%	0.14	9.95%
FG-95-91	4,697,778	4,118,096	4,118,096	87.66%	0.13	8.60%
FG-95-94	5,914,159	5,208,819	5,208,819	88.07%	0.16	10.50%
FG-95-95	5,014,037	4,406,235	4,406,235	87.88%	0.14	8.97%
FG-95-97	4,997,912	4,366,462	4,366,462	87.37%	0.13	10.91%
FG-95-98	4,698,251	4,113,844	4,113,844	87.56%	0.13	10.99%
FG-95-99	4,261,226	3,733,197	3,733,197	87.61%	0.12	9.93%

Supplementary Table 3 Light-coverage whole genome sequencing of single cells from year 23 and year 28

Sample_ID	Total_Raw_Reads	Total_Mapped_Reads	Uniquely_Mapped_Reads	Mapping_Ratio	Depth	Coverage
Year_28_1	4,880,646	4,352,370	4,352,370	89.18%	0.13	8.48%
Year_28_101	5,618,637	4,953,585	4,953,585	88.16%	0.15	9.50%
Year_28_102	6,687,556	5,902,972	5,902,972	88.27%	0.18	12.70%
Year_28_103	5,996,990	5,285,985	5,285,985	88.14%	0.16	12.29%
Year_28_104	6,016,013	5,284,337	5,284,337	87.84%	0.16	11.58%
Year_28_105	4,579,686	4,023,293	4,023,293	87.85%	0.12	8.43%
Year_28_107	4,693,787	4,136,609	4,136,609	88.13%	0.13	8.70%
Year_28_108	4,420,269	3,876,384	3,876,384	87.70%	0.12	8.55%
Year_28_16	4,573,877	4,070,774	4,070,774	89.00%	0.13	8.80%
Year_28_19	4,196,608	3,752,958	3,752,958	89.43%	0.12	8.16%
Year_28_20	5,051,755	4,504,961	4,504,961	89.18%	0.14	9.18%
Year_28_25	4,407,329	3,918,645	3,918,645	88.91%	0.12	9.05%
Year_28_28	766,229	766,229	766,229	100.00%	0.02	1.60%
Year_28_29	4,600,911	4,089,339	4,089,339	88.88%	0.13	9.24%
Year_28_3	4,533,015	4,018,675	4,018,675	88.65%	0.12	6.28%
Year_28_31	5,127,462	4,559,775	4,559,775	88.93%	0.14	8.45%
Year_28_33	4,370,513	3,859,114	3,859,114	88.30%	0.12	7.49%
Year_28_34	4,900,398	4,335,787	4,335,787	88.48%	0.13	9.00%
Year_28_36	4,794,808	4,259,153	4,259,153	88.83%	0.13	7.94%
Year_28_38	4,475,416	3,962,120	3,962,120	88.53%	0.12	7.16%
Year_28_4	4,506,586	3,992,044	3,992,044	88.58%	0.12	7.63%
Year_28_42	4,360,154	3,881,477	3,881,477	89.02%	0.12	8.22%
Year_28_43	5,061,369	4,511,448	4,511,448	89.13%	0.14	9.26%
Year_28_45	4,270,733	3,754,547	3,754,547	87.91%	0.12	8.10%
Year_28_46	4,283,566	3,782,553	3,782,553	88.30%	0.12	9.50%
Year_28_47	4,954,105	4,365,982	4,365,982	88.13%	0.13	9.01%
Year_28_50	4,785,370	4,203,879	4,203,879	87.85%	0.13	9.00%
Year_28_51	5,501,411	4,860,751	4,860,751	88.35%	0.15	10.68%
Year_28_52	5,037,927	4,438,805	4,438,805	88.11%	0.14	9.38%

Year_28_53	5,218,838	4,606,395	4,606,395	88.26%	0.14	9.70%
Year_28_54	4,492,678	3,970,463	3,970,463	88.38%	0.12	9.31%
Year_28_55	4,486,415	3,949,813	3,949,813	88.04%	0.12	9.36%
Year_28_57	4,362,166	3,861,013	3,861,013	88.51%	0.12	9.60%
Year_28_59	4,349,893	3,828,885	3,828,885	88.02%	0.12	8.52%
Year_28_60	4,372,478	3,853,560	3,853,560	88.13%	0.12	8.70%
Year_28_61	4,409,898	3,902,986	3,902,986	88.51%	0.12	9.13%
Year_28_62	5,011,247	4,421,803	4,421,803	88.24%	0.14	9.03%
Year_28_64	5,706,376	5,053,528	5,053,528	88.56%	0.16	11.29%
Year_28_65	4,211,908	3,723,022	3,723,022	88.39%	0.11	9.17%
Year_28_67	3,883,006	3,430,358	3,430,358	88.34%	0.11	7.86%
Year_28_68	4,668,945	4,112,032	4,112,032	88.07%	0.13	7.04%
Year_28_69	4,330,275	3,810,785	3,810,785	88.00%	0.12	8.67%
Year_28_7	4,672,979	4,157,573	4,157,573	88.97%	0.13	8.71%
Year_28_70	4,103,044	3,622,282	3,622,282	88.28%	0.11	9.06%
Year_28_71	5,877,376	5,191,605	5,191,605	88.33%	0.16	11.74%
Year_28_72	4,720,247	4,179,129	4,179,129	88.54%	0.13	10.15%
Year_28_74	4,338,554	3,843,118	3,843,118	88.58%	0.12	9.24%
Year_28_76	5,160,014	4,551,706	4,551,706	88.21%	0.14	9.74%
Year_28_77	5,177,235	4,571,839	4,571,839	88.31%	0.14	10.43%
Year_28_78	5,391,797	4,752,220	4,752,220	88.14%	0.15	10.38%
Year_28_79	6,091,028	5,367,992	5,367,992	88.13%	0.17	11.90%
Year_28_80	4,100,598	3,618,095	3,618,095	88.23%	0.11	8.88%
Year_28_82	4,440,875	3,908,902	3,908,902	88.02%	0.12	8.61%
Year_28_84	5,761,687	5,086,547	5,086,547	88.28%	0.16	11.10%
Year_28_85	5,800,620	5,111,273	5,111,273	88.12%	0.16	10.17%
Year_28_86	5,516,315	4,863,455	4,863,455	88.16%	0.15	9.91%
Year_28_88	5,321,739	4,698,382	4,698,382	88.29%	0.15	10.92%
Year_28_92	6,156,000	5,056,901	5,056,901	82.15%	0.16	10.25%
Year_28_93	6,271,194	5,503,772	5,503,772	87.76%	0.17	11.39%
Year_28_94	6,642,946	5,881,425	5,881,425	88.54%	0.18	10.98%
Year_28_96	5,233,511	4,616,662	4,616,662	88.21%	0.14	10.46%

Year_28_99	5,049,381	4,463,514	4,463,514	88.40%	0.14	10.07%
Year_23_1	2,984,477	2,611,938	2,611,938	87.52%	0.08	7.13%
Year_23_10	4,043,962	3,560,688	3,560,688	88.05%	0.00	0.25%
Year_23_11	4,345,944	3,797,620	3,797,620	87.38%	0.12	9.86%
Year_23_13	3,773,210	3,300,467	3,300,467	87.47%	0.10	8.86%
Year_23_14	4,468,956	3,900,205	3,900,205	87.27%	0.12	10.28%
Year_23_15	3,666,253	3,205,429	3,205,429	87.43%	0.10	8.70%
Year_23_16	3,729,272	3,268,316	3,268,316	87.64%	0.10	8.89%
Year_23_17	4,184,100	3,671,854	3,671,854	87.76%	0.11	10.12%
Year_23_18	4,248,961	3,730,999	3,730,999	87.81%	0.12	10.38%
Year_23_19	4,063,241	3,556,877	3,556,877	87.54%	0.11	9.40%
Year_23_2	3,477,954	3,043,859	3,043,859	87.52%	0.09	8.22%
Year_23_20	4,227,993	3,701,063	3,701,063	87.54%	0.11	9.78%
Year_23_21	4,039,983	3,532,087	3,532,087	87.43%	0.11	9.72%
Year_23_22	3,704,915	3,240,122	3,240,122	87.45%	0.10	8.59%
Year_23_23	3,678,096	3,214,409	3,214,409	87.39%	0.10	8.55%
Year_23_26	4,104,753	3,590,791	3,590,791	87.48%	0.11	9.29%
Year_23_27	3,569,634	3,154,781	3,154,781	88.38%	0.10	8.63%
Year_23_28	3,904,768	3,422,088	3,422,088	87.64%	0.11	9.29%
Year_23_3	4,427,740	3,872,356	3,872,356	87.46%	0.12	10.10%
Year_23_32	3,830,468	3,386,749	3,386,749	88.42%	0.10	7.83%
Year_23_34	4,427,848	3,921,457	3,921,457	88.56%	0.12	8.49%
Year_23_35	4,229,861	3,751,036	3,751,036	88.68%	0.12	7.79%
Year_23_4	3,802,757	3,318,862	3,318,862	87.28%	0.10	8.42%
Year_23_42	4,432,836	3,923,615	3,923,615	88.51%	0.12	8.90%
Year_23_46	3,548,963	3,144,737	3,144,737	88.61%	0.10	7.51%
Year_23_49	4,170,947	3,702,730	3,702,730	88.77%	0.11	8.63%
Year_23_5	4,249,966	3,722,513	3,722,513	87.59%	0.11	9.71%
Year_23_51	4,700,888	4,166,530	4,166,530	88.63%	0.13	8.97%
Year_23_53	4,296,290	3,803,043	3,803,043	88.52%	0.12	8.48%
Year_23_55	4,584,340	4,071,614	4,071,614	88.82%	0.13	9.22%
Year_23_56	4,138,890	3,674,982	3,674,982	88.79%	0.11	8.50%

Year_23_57	4,297,494	3,815,105	3,815,105	88.78%	0.12	8.86%
Year_23_58	4,614,681	4,102,584	4,102,584	88.90%	0.13	7.72%
Year_23_6	3,337,781	2,923,067	2,923,067	87.58%	0.09	7.76%
Year_23_60	4,090,621	3,623,711	3,623,711	88.59%	0.11	8.90%
Year_23_64	3,988,909	3,529,565	3,529,565	88.48%	0.11	7.91%
Year_23_67	3,713,302	3,282,393	3,282,393	88.40%	0.10	7.42%
Year_23_68	5,781,770	5,104,528	5,104,528	88.29%	0.16	10.54%
Year_23_7	3,542,386	3,104,383	3,104,383	87.64%	0.10	8.68%
Year_23_70	5,249,616	4,645,976	4,645,976	88.50%	0.14	9.76%
Year_23_74	5,500,799	4,852,737	4,852,737	88.22%	0.15	10.83%
Year_23_75	5,326,727	4,675,481	4,675,481	87.77%	0.14	9.77%
Year_23_76	5,006,100	4,394,686	4,394,686	87.79%	0.14	8.36%
Year_23_79	4,950,643	4,353,539	4,353,539	87.94%	0.13	8.74%
Year_23_8	3,846,393	3,373,106	3,373,106	87.70%	0.10	9.48%
Year_23_87	4,843,872	4,262,462	4,262,462	88.00%	0.13	8.48%
Year_23_9	4,171,632	3,657,244	3,657,244	87.67%	0.11	9.95%
Year_23_90	5,263,771	4,647,658	4,647,658	88.30%	0.14	9.95%
Year_23_91	4,697,778	4,118,096	4,118,096	87.66%	0.13	8.60%
Year_23_94	5,914,159	5,208,819	5,208,819	88.07%	0.16	10.50%
Year_23_95	5,014,037	4,406,235	4,406,235	87.88%	0.14	8.97%
Year_23_97	4,997,912	4,366,462	4,366,462	87.37%	0.13	10.91%
Year_23_98	4,698,251	4,113,844	4,113,844	87.56%	0.13	10.99%
Year_23_99	4,261,226	3,733,197	3,733,197	87.61%	0.12	9.93%

Supplemetary table 4 Single-cell RNA-seq of 355 cells

Sample_ID	Total_Raw_Reads	Total_Clean_Reads	Uniquely_Mapped_Reads	Uniquely_mapped_ratio	RPKM>0	RPKM>0.1	RPKM>1
Year_10_001	13,585,819	10,184,391	4,879,062	47.91%	8,987	7,723	3,234
Year_10_002	20,274,838	19,678,842	4,985,431	25.33%	8,502	6,997	2,758
Year_10_003	12,846,355	10,300,221	4,234,241	41.11%	11,434	9,892	3,111
Year_10_004	4,428,937	4,204,426	2,391,900	56.89%	9,490	9,139	4,447
Year_10_005	13,177,531	11,687,977	6,089,482	52.10%	7,945	6,458	3,449
Year_10_006	11,966,984	11,478,425	5,186,237	45.18%	8,101	6,811	2,948
Year_10_007	13,028,936	12,892,115	7,207,101	55.90%	9,149	7,215	3,708
Year_10_008	14,901,118	12,199,068	4,438,619	36.38%	8,627	7,371	2,966
Year_10_009	8,206,672	6,048,916	719,183	11.89%	8,358	8,348	5,910
Year_10_010	12,457,609	9,497,250	1,402,981	14.77%	6,542	6,451	2,740
Year_10_011	13,510,213	12,684,039	6,664,749	52.54%	9,463	7,516	3,260
Year_10_012	11,462,614	11,231,286	5,940,423	52.89%	11,697	10,307	5,869
Year_10_013	11,016,312	10,472,654	3,250,902	31.04%	10,241	9,687	5,411
Year_10_014	9,737,298	5,399,413	707,120	13.10%	12,004	11,998	9,605
Year_10_015	7,235,409	5,945,065	2,089,945	35.15%	13,963	13,711	7,727
Year_10_016	11,861,352	11,493,769	59,008	0.51%	4,173	4,173	4,169
Year_10_017	5,805,857	5,701,710	1,808,938	31.73%	14,528	14,437	11,533
Year_10_018	3,393,635	3,336,700	1,143,780	34.28%	13,803	13,785	11,526
Year_10_019	14,486,202	13,515,438	7,943,840	58.78%	12,562	10,117	4,804
Year_10_020	9,108,392	4,907,124	174,117	3.55%	6,258	6,258	6,119
Year_10_021	11,408,343	9,519,753	4,157,279	43.67%	8,099	7,101	3,120
Year_10_022	4,808,564	4,775,599	2,479,339	51.92%	7,331	7,074	3,935
Year_10_023	10,315,700	7,927,446	2,546,539	32.12%	10,721	10,371	5,635
Year_10_024	11,707,357	8,718,676	1,961,450	22.50%	9,876	9,729	6,098
Year_10_025	8,821,214	8,136,964	4,457,254	54.78%	11,696	10,573	5,651
Year_10_026	10,291,974	10,018,397	4,581,440	45.73%	7,118	6,005	2,356
Year_10_027	10,821,078	10,282,042	29,139	0.28%	3,261	3,261	3,260
Year_10_028	10,916,140	9,783,863	1,096,583	11.21%	8,902	8,859	5,368

Year_10_029	14,143,442	13,774,023	6,905,292	50.13%	9,645	7,694	3,679
Year_10_030	41,879,686	37,643,658	21,164,658	56.22%	15,435	10,061	4,968
Year_10_031	20,356,297	20,280,923	11,780,497	58.09%	8,107	5,198	2,291
Year_10_032	16,602,960	16,445,419	10,467,107	63.65%	9,203	7,015	3,779
Year_10_033	18,103,100	17,151,489	8,014,940	46.73%	13,948	11,606	5,618
Year_10_034	27,379,740	20,991,447	3,866,343	18.42%	8,112	7,206	2,767
Year_10_035	24,170,597	24,037,317	8,082,848	33.63%	10,265	8,326	4,228
Year_10_036	40,231,171	37,176,076	11,585,056	31.16%	17,097	13,871	7,329
Year_10_037	29,756,627	27,692,058	14,202,790	51.29%	15,488	10,744	4,418
Year_10_038	3,051,868	3,046,831	1,646,546	54.04%	5,422	5,317	2,875
Year_20_001	6,314,810	6,003,939	3,848,838	64.11%	8,511	7,562	2,949
Year_20_002	6,274,522	5,708,882	2,900,747	50.81%	11,263	10,603	4,293
Year_20_003	3,488,063	3,409,770	2,078,332	60.95%	7,310	7,081	3,430
Year_20_004	2,708,836	2,660,099	1,678,830	63.11%	7,697	7,608	4,396
Year_20_005	4,086,154	3,979,506	2,626,586	66.00%	8,640	8,104	3,135
Year_20_006	2,806,995	2,732,420	1,842,663	67.44%	8,016	7,836	3,970
Year_20_007	5,530,584	5,287,896	3,445,903	65.17%	9,747	8,894	3,369
Year_20_008	4,275,078	4,128,663	2,730,555	66.14%	10,304	9,876	4,716
Year_20_009	2,979,183	2,805,845	1,791,160	63.84%	7,505	7,348	3,642
Year_20_010	5,577,335	5,376,310	3,575,013	66.50%	11,482	10,718	4,766
Year_20_011	5,947,211	5,825,745	3,710,517	63.69%	8,566	7,620	3,008
Year_20_012	7,935,789	7,337,669	4,120,290	56.15%	9,723	8,747	3,971
Year_20_013	4,218,223	4,092,677	2,765,683	67.58%	8,470	8,032	3,726
Year_20_014	5,198,926	4,385,130	394,499	9.00%	5,442	5,442	4,636
Year_20_015	5,997,241	5,758,012	3,227,729	56.06%	9,353	8,768	3,877
Year_20_016	1,921,837	1,907,529	1,256,149	65.85%	6,530	6,488	3,715
Year_20_017	2,377,110	2,303,496	1,231,197	53.45%	7,841	7,799	4,969
Year_20_018	6,222,811	5,683,584	3,369,072	59.28%	11,780	11,241	5,974
Year_20_019	7,258,085	6,738,745	3,696,214	54.85%	10,637	9,903	5,412
Year_20_020	5,242,846	4,981,945	2,855,951	57.33%	8,717	8,152	3,311
Year_20_021	7,207,652	6,993,350	3,995,714	57.14%	10,779	9,662	3,759
Year_20_022	3,019,234	2,861,079	1,541,648	53.88%	8,008	7,909	4,572

Year_20_023	4,925,358	4,800,926	2,950,618	61.46%	10,178	9,534	3,760
Year_20_024	7,380,671	7,077,363	4,125,722	58.29%	10,029	9,101	4,462
Year_20_025	6,199,229	6,046,625	3,793,619	62.74%	10,053	9,156	4,014
Year_20_026	5,007,307	4,820,912	3,150,881	65.36%	11,451	10,859	5,420
Year_20_027	6,569,232	6,292,330	4,142,827	65.84%	10,390	9,436	4,271
Year_20_028	7,134,713	6,558,085	2,113,167	32.22%	10,178	9,870	4,554
Year_20_029	3,627,919	3,574,636	2,281,847	63.83%	10,738	10,450	5,233
Year_20_030	7,175,450	7,031,329	4,606,254	65.51%	8,608	7,358	2,819
Year_20_031	4,245,407	4,002,651	2,558,064	63.91%	8,036	7,675	3,821
Year_20_032	5,601,379	5,511,876	3,352,968	60.83%	8,631	7,897	3,268
Year_20_033	2,901,988	2,861,214	1,917,338	67.01%	8,380	8,179	4,241
Year_20_034	963,082	941,952	541,009	57.43%	6,441	6,437	4,998
Year_20_035	6,079,844	5,781,458	3,480,519	60.20%	9,733	8,862	3,021
Year_20_036	2,331,545	2,178,918	1,313,926	60.30%	8,469	8,406	4,678
Year_20_037	3,816,761	3,700,540	2,368,918	64.02%	7,555	7,229	3,279
Year_20_038	2,926,592	2,786,991	1,700,035	61.00%	8,477	8,323	3,702
Year_20_039	10,511,053	10,165,813	6,519,822	64.13%	9,700	7,590	3,004
Year_20_040	19,666,246	18,873,047	10,739,093	56.90%	12,694	10,075	5,239
Year_20_041	7,364,970	7,267,123	5,064,688	69.69%	11,032	9,649	4,154
Year_20_042	7,187,070	7,122,614	4,579,851	64.30%	9,885	8,719	3,946
Year_20_043	9,336,898	9,268,055	6,390,422	68.95%	14,154	11,789	4,654
Year_20_044	9,577,704	9,330,488	6,060,715	64.96%	12,816	10,683	3,864
Year_20_045	8,077,266	8,015,275	5,376,371	67.08%	10,493	8,752	3,353
Year_20_046	10,563,686	10,253,674	6,673,062	65.08%	12,190	10,164	4,622
Year_20_047	9,336,898	9,268,055	6,391,151	68.96%	14,159	11,777	4,641
Year_20_048	9,724,671	9,477,042	6,278,488	66.25%	11,009	9,126	3,756
Year_23_001	6,032,607	6,005,844	3,390,203	56.45%	8,391	7,815	3,554
Year_23_002	5,563,806	5,535,883	3,025,047	54.64%	7,980	7,517	3,492
Year_23_003	4,800,554	4,777,902	2,584,870	54.10%	6,651	6,315	2,846
Year_23_004	5,515,033	5,489,150	2,983,819	54.36%	6,572	6,113	2,586
Year_23_005	5,017,082	4,989,586	551,968	11.06%	3,269	3,268	2,259
Year_23_006	3,920,933	3,898,002	469,363	12.04%	2,756	2,756	2,080

Year_23_007	18,793,789	18,748,662	10,984,529	58.59%	9,355	6,742	2,885
Year_23_008	19,908,930	19,850,414	10,033,532	50.55%	8,435	5,858	2,283
Year_23_009	21,397,371	21,328,804	11,587,913	54.33%	8,520	5,661	2,154
Year_23_010	19,263,558	19,211,268	10,694,959	55.67%	9,846	7,370	3,225
Year_23_011	19,243,822	19,186,885	9,015,575	46.99%	6,651	4,719	1,931
Year_23_013	4,286,680	4,266,267	2,302,450	53.97%	5,640	5,377	2,563
Year_23_014	5,328,261	5,296,614	2,632,310	49.70%	5,750	5,425	2,318
Year_23_015	5,514,665	5,490,502	2,713,801	49.43%	6,440	6,081	2,470
Year_23_016	5,259,270	5,235,958	2,342,281	44.73%	5,899	5,658	2,506
Year_23_017	5,182,916	5,149,116	2,300,809	44.68%	5,044	4,800	1,986
Year_23_018	6,104,767	6,072,746	2,460,037	40.51%	5,828	5,548	2,276
Year_23_019	24,944,607	24,789,976	12,824,324	51.73%	9,727	7,005	3,010
Year_23_020	28,474,534	28,300,757	15,129,271	53.46%	10,811	7,664	3,329
Year_23_021	21,366,423	21,258,777	11,934,981	56.14%	8,409	5,811	2,477
Year_23_022	22,535,926	22,427,850	10,294,505	45.90%	8,098	5,695	2,216
Year_23_023	26,477,189	26,282,305	12,881,115	49.01%	7,486	4,560	1,898
Year_23_024	25,041,240	24,790,179	10,790,709	43.53%	7,894	5,292	2,100
Year_23_025	5,010,637	4,984,044	2,909,609	58.38%	6,224	5,778	2,609
Year_23_026	5,325,585	5,300,324	2,982,738	56.27%	7,074	6,649	3,170
Year_23_027	6,351,489	6,301,241	3,009,096	47.75%	5,504	5,070	2,019
Year_23_028	5,242,679	5,185,959	2,490,001	48.01%	5,883	5,583	2,501
Year_23_030	19,850,628	19,761,254	10,251,462	51.88%	11,476	9,244	4,777
Year_23_031	23,296,089	23,176,653	14,297,908	61.69%	11,508	8,639	4,311
Year_23_033	21,041,973	20,955,926	11,023,358	52.60%	7,653	5,336	2,316
Year_23_085	5,954,834	5,932,346	3,316,790	55.91%	6,125	5,555	2,405
Year_23_086	4,659,828	4,640,804	2,680,913	57.77%	5,004	4,678	2,110
Year_23_087	4,371,942	4,348,121	2,431,293	55.92%	4,916	4,644	1,892
Year_23_088	5,339,089	5,317,278	2,592,270	48.75%	4,615	4,303	1,755
Year_23_089	5,746,074	5,723,647	3,322,551	58.05%	5,240	4,685	2,090
Year_23_090	4,563,961	4,545,170	2,536,283	55.80%	5,249	4,968	2,201
Year_23_091	4,501,894	4,484,312	715,790	15.96%	2,629	2,626	1,584
Year_23_092	4,533,970	4,516,488	2,691,943	59.60%	6,077	5,745	2,755

Year_23_093	5,423,403	5,401,344	3,294,680	61.00%	5,949	5,417	2,448
Year_23_094	4,801,002	4,764,323	2,512,132	52.73%	5,538	5,247	2,332
Year_23_095	5,855,133	5,833,316	3,147,671	53.96%	5,122	4,679	2,066
Year_23_096	5,550,482	5,352,989	2,728,986	50.98%	6,145	5,740	2,380
Year_23_097	4,998,060	4,979,397	3,061,816	61.49%	6,238	5,797	2,696
Year_23_098	5,503,947	5,483,692	3,570,648	65.11%	6,834	6,165	2,662
Year_23_099	5,420,891	5,399,995	3,456,921	64.02%	6,395	5,820	2,638
Year_23_100	5,317,813	5,296,733	3,217,404	60.74%	6,149	5,630	2,480
Year_23_101	5,220,382	5,200,396	3,081,919	59.26%	6,062	5,575	2,498
Year_23_102	4,383,219	4,360,140	2,748,734	63.04%	6,756	6,345	2,951
Year_23_103	4,749,961	4,730,934	2,811,618	59.43%	7,704	7,296	3,511
Year_23_104	6,003,826	5,981,679	3,477,945	58.14%	5,586	5,005	2,334
Year_23_105	5,244,203	5,220,409	2,560,321	49.04%	4,909	4,575	1,874
Year_23_106	6,005,318	5,983,610	3,723,861	62.23%	4,220	3,645	1,405
Year_23_107	5,680,684	5,657,055	3,455,915	61.09%	7,631	7,009	3,302
Year_23_108	7,331,529	7,258,127	4,010,792	55.26%	8,012	6,977	2,980
Year_23_109	5,480,418	5,456,014	3,003,455	55.05%	6,058	5,582	2,535
Year_23_110	5,048,251	5,024,316	2,655,987	52.86%	5,816	5,438	2,420
Year_23_111	5,442,075	5,419,037	3,393,168	62.62%	6,705	6,097	2,712
Year_23_112	4,875,930	4,856,184	2,657,904	54.73%	6,096	5,687	2,274
Year_23_113	3,843,800	3,827,765	2,193,366	57.30%	4,564	4,359	1,728
Year_23_114	5,400,858	5,379,528	2,857,733	53.12%	6,708	6,263	2,676
Year_23_115	6,175,671	6,151,837	3,863,352	62.80%	6,380	5,637	2,548
Year_23_116	6,315,607	6,292,935	4,062,100	64.55%	6,069	5,225	2,302
Year_23_117	4,924,185	4,901,088	2,634,945	53.76%	5,294	4,957	2,023
Year_23_118	4,286,133	4,269,067	2,594,732	60.78%	5,017	4,706	2,030
Year_23_119	3,857,533	3,841,779	2,492,120	64.87%	5,422	5,144	2,369
Year_23_120	7,120,750	7,000,878	3,968,590	56.69%	5,676	4,965	2,008
Year_23_121	5,603,464	5,570,744	2,468,370	44.31%	4,832	4,542	1,860
Year_23_122	4,402,454	4,382,388	2,287,848	52.21%	4,173	3,964	1,743
Year_23_123	4,376,165	4,355,377	2,653,012	60.91%	6,973	6,597	3,196
Year_23_124	4,817,781	4,800,126	3,035,434	63.24%	6,821	6,326	2,938

Year_23_125	4,729,136	4,712,347	2,911,252	61.78%	7,310	6,895	3,495
Year_23_126	4,201,816	4,182,389	2,637,901	63.07%	8,152	7,821	4,061
Year_23_127	6,021,470	5,995,894	3,614,147	60.28%	6,717	5,936	2,659
Year_23_128	5,141,952	5,121,772	3,113,795	60.80%	5,745	5,287	2,477
Year_23_129	4,182,890	4,164,039	2,128,632	51.12%	3,993	3,805	1,506
Year_23_130	3,763,848	3,748,979	2,004,378	53.46%	4,595	4,455	2,032
Year_23_131	3,770,251	3,754,883	1,942,737	51.74%	3,908	3,785	1,708
Year_23_132	6,473,809	6,420,943	3,582,547	55.79%	6,741	6,043	2,496
Year_23_133	5,436,008	5,385,067	2,756,030	51.18%	5,198	4,823	1,972
Year_23_134	5,758,608	5,737,202	3,225,193	56.22%	5,776	5,195	2,169
Year_23_135	5,693,396	5,668,429	2,843,408	50.16%	5,623	5,206	2,078
Year_23_136	3,989,428	3,974,596	2,200,795	55.37%	5,201	4,966	2,170
Year_23_137	5,521,476	5,499,426	3,253,963	59.17%	5,908	5,331	2,406
Year_23_138	5,420,489	5,399,521	2,637,503	48.85%	6,832	6,515	3,158
Year_23_139	5,668,517	5,646,772	3,525,577	62.44%	6,735	6,148	2,728
Year_23_140	5,382,095	5,359,353	2,852,701	53.23%	5,405	4,966	2,083
Year_23_141	3,833,722	3,816,479	1,914,479	50.16%	4,607	4,469	2,009
Year_23_142	3,581,110	3,566,832	1,769,885	49.62%	5,182	5,056	2,230
Year_23_143	4,194,827	4,177,009	2,077,461	49.74%	4,217	4,069	1,781
Year_23_144	6,951,739	6,897,711	3,001,764	43.52%	6,422	5,972	2,914
Year_23_145	5,033,162	5,012,931	2,733,446	54.53%	5,124	4,784	2,061
Year_23_146	5,337,057	5,314,290	2,768,164	52.09%	5,714	5,333	2,129
Year_23_147	4,575,460	4,555,145	2,418,726	53.10%	5,723	5,420	2,406
Year_23_148	4,568,481	4,547,457	2,669,373	58.70%	6,159	5,793	2,504
Year_23_149	5,906,631	5,884,667	3,503,319	59.53%	5,826	5,243	2,178
Year_23_150	7,131,651	7,105,158	3,719,185	52.34%	6,691	5,906	2,184
Year_23_151	6,007,730	5,986,706	3,541,371	59.15%	6,351	5,641	2,310
Year_23_152	6,048,808	6,027,193	3,487,635	57.86%	5,321	4,790	2,218
Year_23_153	5,179,686	5,159,718	2,812,546	54.51%	5,893	5,461	2,215
Year_23_154	4,562,740	4,543,746	2,655,375	58.44%	4,933	4,610	2,020
Year_23_155	4,145,194	4,127,498	2,428,838	58.85%	4,514	4,303	2,046
Year_23_156	11,218,595	11,006,122	5,558,535	50.50%	8,151	6,652	2,663

Year_23_157	6,860,800	6,837,215	4,102,764	60.01%	6,682	5,804	2,399
Year_23_158	5,990,676	5,965,940	2,913,773	48.84%	5,168	4,741	2,003
Year_23_159	5,051,748	5,030,005	2,735,211	54.38%	5,784	5,406	2,512
Year_23_160	3,984,572	3,964,693	2,101,767	53.01%	4,496	4,290	1,887
Year_23_161	5,125,713	5,107,289	2,962,033	58.00%	5,274	4,882	2,216
Year_23_162	4,311,483	4,292,665	2,395,643	55.81%	5,424	5,168	2,278
Year_23_163	5,865,834	5,842,993	3,713,817	63.56%	7,423	6,707	2,952
Year_23_164	7,002,724	6,978,793	3,848,943	55.15%	7,300	6,544	3,056
Year_23_165	4,074,129	4,056,878	2,052,948	50.60%	4,260	4,094	1,715
Year_23_166	5,150,229	5,130,445	2,859,558	55.74%	4,938	4,559	1,985
Year_23_167	7,384,305	7,357,779	4,067,554	55.28%	5,575	4,775	1,874
Year_23_168	5,388,697	5,366,908	3,236,235	60.30%	6,585	6,012	2,791
Year_23_169	4,776,783	4,755,804	2,599,377	54.66%	4,961	4,650	2,015
Year_23_170	5,057,221	5,036,179	2,530,912	50.25%	5,251	4,912	1,923
Year_23_171	5,244,906	5,220,576	3,250,484	62.26%	6,851	6,243	2,571
Year_23_172	4,971,283	4,946,810	3,033,488	61.32%	7,384	6,900	3,162
Year_23_173	5,042,679	5,024,282	2,877,993	57.28%	5,786	5,396	2,478
Year_23_174	5,439,379	5,419,461	3,003,599	55.42%	5,224	4,833	2,203
Year_23_175	5,571,187	5,548,138	2,828,515	50.98%	4,696	4,336	1,928
Year_23_176	4,205,454	4,187,171	1,720,393	41.09%	4,508	4,390	1,849
Year_23_177	4,378,707	4,351,364	2,259,206	51.92%	5,664	5,433	2,504
Year_23_178	4,347,940	4,327,230	2,629,598	60.77%	6,811	6,485	3,090
Year_23_179	5,243,118	5,223,497	3,142,354	60.16%	14,721	13,879	4,861
Year_23_180	4,904,048	4,875,012	2,647,758	54.31%	6,177	5,846	2,764
Year_26_001	5,200,868	3,359,824	1,977,915	58.87%	9,470	9,218	3,959
Year_26_002	7,031,358	6,573,441	4,144,838	63.05%	9,385	8,471	4,339
Year_26_003	12,274,995	11,903,466	6,130,888	51.51%	11,626	10,373	6,344
Year_26_004	9,535,316	8,336,207	4,201,473	50.40%	12,232	11,213	5,327
Year_26_005	9,292,104	8,104,841	1,369,126	16.89%	13,651	13,576	8,674
Year_26_006	10,123,780	7,391,796	1,062,296	14.37%	10,285	10,242	6,000
Year_26_007	14,237,953	13,243,961	5,730,000	43.27%	10,010	8,414	3,838
Year_26_008	16,062,206	16,009,748	6,150,806	38.42%	7,940	6,364	2,878

Year_26_009	7,679,540	7,276,013	3,659,127	50.29%	13,988	13,268	7,534
Year_26_010	7,252,809	5,811,853	549,777	9.46%	8,961	8,956	6,913
Year_26_011	9,820,957	6,058,181	302,033	4.99%	5,812	5,812	5,290
Year_26_012	10,816,888	10,594,909	5,237,114	49.43%	7,974	6,646	3,180
Year_26_013	13,134,103	12,845,095	6,027,134	46.92%	10,423	8,883	5,005
Year_26_014	16,125,280	14,416,359	5,057,193	35.08%	9,802	8,453	3,707
Year_26_015	6,704,461	5,763,662	872,675	15.14%	13,021	13,011	11,117
Year_26_016	13,178,440	10,418,487	532,852	5.11%	7,879	7,876	6,296
Year_26_017	10,518,214	10,361,474	5,104,368	49.26%	11,204	10,246	6,057
Year_26_018	12,512,917	11,071,053	3,347,305	30.23%	8,878	8,183	3,541
Year_26_019	9,357,772	9,111,157	3,416,111	37.49%	8,139	7,518	3,278
Year_26_020	9,982,855	9,942,949	4,112,327	41.36%	10,087	9,235	4,887
Year_26_021	10,073,777	9,042,529	3,792,817	41.94%	8,861	8,100	3,611
Year_26_022	17,372,674	16,761,932	7,427,246	44.31%	12,472	10,186	4,562
Year_26_023	9,682,104	5,693,859	342,296	6.01%	8,164	8,164	7,324
Year_26_024	10,189,011	8,816,024	631,136	7.16%	6,854	6,849	4,852
Year_26_025	10,187,050	10,030,709	591,531	5.90%	5,354	5,353	3,890
Year_26_026	6,787,385	5,426,336	2,852,510	52.57%	10,309	9,696	4,271
Year_26_027	26,920,402	26,868,027	15,840,650	58.96%	9,437	5,959	2,837
Year_26_028	34,143,491	33,664,044	18,226,106	54.14%	10,206	6,524	3,178
Year_26_029	29,679,004	29,490,586	6,169,119	20.92%	8,784	7,249	3,243
Year_26_030	27,892,821	27,619,126	12,520,129	45.33%	8,809	5,972	2,810
Year_26_031	19,846,102	19,707,894	11,947,763	60.62%	7,603	5,036	2,327
Year_26_032	20,638,943	17,883,388	8,063,613	45.09%	16,309	13,038	4,020
Year_26_033	20,567,992	20,530,372	11,094,684	54.04%	8,818	6,215	2,868
Year_26_034	20,950,601	20,912,814	10,685,029	51.09%	9,659	7,168	3,695
Year_26_035	18,754,467	18,645,276	10,543,577	56.55%	9,596	7,224	3,646
Year_28_001	4,438,038	4,426,132	2,820,707	63.73%	8,776	8,415	4,804
Year_28_002	5,203,487	5,192,395	2,376,809	45.77%	4,650	4,385	1,785
Year_28_003	4,827,292	4,804,202	513,507	10.69%	3,679	3,679	2,783
Year_28_004	4,548,419	4,528,571	147,488	3.26%	2,829	2,829	2,775
Year_28_005	3,896,098	3,887,127	2,085,917	53.66%	5,231	5,052	2,209

Year_28_006	5,057,212	5,041,299	382,514	7.59%	3,204	3,203	2,552
Year_28_007	4,406,282	4,396,525	2,084,287	47.41%	5,329	5,126	2,085
Year_28_008	4,606,780	4,594,764	2,664,031	57.98%	7,423	7,070	3,405
Year_28_009	4,676,126	4,664,713	2,616,107	56.08%	7,949	7,647	4,026
Year_28_010	3,802,191	3,792,641	2,262,859	59.66%	7,579	7,344	4,034
Year_28_011	4,681,980	4,669,388	2,675,197	57.29%	8,969	8,613	4,873
Year_28_012	4,131,289	4,119,450	2,324,491	56.43%	7,232	6,984	3,494
Year_28_013	4,430,905	4,420,022	2,503,419	56.64%	6,138	5,861	2,744
Year_28_014	6,391,649	6,372,745	327,911	5.15%	2,925	2,925	2,504
Year_28_016	4,525,108	4,513,313	2,786,269	61.73%	6,783	6,381	2,978
Year_28_017	4,323,213	4,311,630	2,412,218	55.95%	5,605	5,354	2,478
Year_28_018	3,598,429	3,589,227	2,248,522	62.65%	7,035	6,833	3,640
Year_28_019	4,652,450	4,639,205	2,528,131	54.49%	6,266	5,948	2,497
Year_28_020	4,653,550	4,643,133	2,838,511	61.13%	7,379	6,987	3,535
Year_28_021	3,891,881	3,881,489	2,477,129	63.82%	8,558	8,282	4,618
Year_28_022	5,415,721	5,402,338	3,237,320	59.92%	10,050	9,573	5,737
Year_28_023	4,010,718	3,999,558	2,256,061	56.41%	8,540	8,308	4,819
Year_28_024	4,500,743	4,487,577	2,853,738	63.59%	7,727	7,304	3,527
Year_28_025	6,510,215	6,488,991	3,940,264	60.72%	6,830	6,001	2,458
Year_28_026	4,833,377	4,810,728	170,180	3.54%	2,733	2,733	2,656
Year_28_027	3,935,217	3,925,035	1,771,191	45.13%	6,820	6,702	3,587
Year_28_028	3,246,144	3,237,853	1,955,343	60.39%	7,253	7,111	3,913
Year_28_029	2,473,478	2,463,561	1,382,156	56.10%	4,470	4,412	2,353
Year_28_030	3,697,779	3,687,202	2,187,078	59.32%	8,219	8,015	4,521
Year_28_031	5,464,819	5,450,201	2,860,122	52.48%	5,730	5,284	2,265
Year_28_032	5,246,672	5,229,148	2,829,826	54.12%	6,085	5,652	2,481
Year_28_033	7,343,508	7,316,295	3,771,310	51.55%	8,351	7,451	2,812
Year_28_034	5,219,237	5,204,763	3,141,033	60.35%	5,569	5,038	2,127
Year_28_036	3,770,347	3,760,626	2,234,441	59.42%	6,822	6,616	3,334
Year_28_037	5,036,709	5,022,545	2,841,902	56.58%	8,077	7,609	3,402
Year_28_038	5,117,699	5,103,804	2,871,026	56.25%	6,517	6,081	2,680
Year_28_039	4,044,491	4,033,099	2,512,470	62.30%	6,473	6,189	3,168

Year_28_040	4,488,243	4,476,723	2,583,779	57.72%	7,530	7,142	3,005
Year_28_041	5,164,474	5,147,424	2,955,694	57.42%	6,462	6,006	2,689
Year_28_042	3,439,597	3,428,953	2,028,144	59.15%	7,668	7,481	3,801
Year_28_043	4,237,228	4,225,159	2,165,755	51.26%	6,032	5,804	2,774
Year_28_044	5,131,765	5,119,662	2,935,719	57.34%	5,827	5,382	2,444
Year_28_045	4,481,596	4,468,372	2,669,773	59.75%	7,119	6,727	3,076
Year_28_046	4,739,872	4,725,769	1,999,308	42.31%	5,529	5,332	2,291
Year_28_047	4,050,049	4,039,042	2,104,937	52.11%	5,568	5,361	2,569
Year_28_048	4,416,631	4,405,719	2,380,232	54.03%	7,579	7,293	3,592
Year_28_049	4,969,445	4,953,485	2,754,700	55.61%	6,828	6,423	2,794
Year_28_050	3,033,475	3,026,008	1,767,676	58.42%	7,108	6,985	3,691
Year_28_051	3,074,578	3,066,697	1,754,127	57.20%	7,091	6,962	3,938
Year_28_052	5,380,631	5,359,999	327,492	6.11%	3,769	3,769	3,229
Year_28_053	4,785,909	4,773,327	2,611,064	54.70%	5,584	5,245	2,360
Year_28_055	5,530,245	5,516,172	3,018,662	54.72%	7,242	6,763	3,193
Year_28_056	3,859,651	3,849,552	2,377,917	61.77%	6,793	6,533	3,278
Year_28_057	4,627,396	4,614,575	2,707,876	58.68%	8,047	7,708	4,018
Year_28_058	4,584,222	4,573,527	2,851,160	62.34%	6,372	5,950	2,784
Year_28_059	5,053,372	5,040,838	3,090,624	61.31%	6,612	6,085	2,502
Year_28_060	3,411,666	3,401,820	1,862,111	54.74%	8,342	8,199	4,683
Year_28_061	3,911,661	3,899,581	2,215,631	56.82%	6,681	6,464	3,066
Year_28_063	4,092,032	4,082,263	2,454,465	60.13%	6,436	6,151	2,844
Year_28_064	5,514,611	5,498,939	3,220,114	58.56%	8,098	7,501	3,471
Year_28_072	5,128,861	5,114,315	2,929,172	57.27%	6,858	6,422	2,681
Year_28_077	4,649,632	4,637,422	2,978,728	64.23%	6,956	6,534	3,126
Year_28_078	4,085,275	4,075,007	2,589,398	63.54%	7,124	6,820	3,188
Year_28_079	3,577,234	3,568,213	2,118,047	59.36%	5,350	5,135	2,397
Year_28_080	3,308,740	3,297,014	441,524	13.39%	4,128	4,128	3,358
Year_28_085	2,531,416	2,525,154	1,419,338	56.21%	5,158	5,089	2,603
Year_28_086	3,785,852	3,774,879	2,222,346	58.87%	8,892	8,701	5,108
Year_28_087	5,003,737	1,994,734	1,253,713	62.85%	6,454	6,418	3,867
Year_28_088	3,446,385	3,436,975	2,020,804	58.80%	6,076	5,886	2,738

Year_28_093	1,740,733	1,735,394	1,017,354	58.62%	5,740	5,716	3,610
Year_28_094	3,988,676	3,971,646	2,529,660	63.69%	7,799	7,500	3,901
Year_28_095	2,895,027	2,880,759	1,117,194	38.78%	6,350	6,314	3,555
Year_28_096	3,845,074	3,826,943	2,083,166	54.43%	6,093	5,909	2,636
Year_28_097	3,723,505	3,714,193	2,016,277	54.29%	7,899	7,702	4,156
Year_28_098	3,141,033	3,132,401	1,579,905	50.44%	6,839	6,754	3,672
Year_28_099	4,133,783	4,122,818	2,232,279	54.14%	6,254	6,019	2,698
Year_28_100	4,389,622	4,377,776	2,150,618	49.13%	6,050	5,807	2,574
Year_28_101	4,220,513	4,207,784	2,630,184	62.51%	9,743	9,447	5,580
Year_28_102	3,424,330	3,416,003	1,996,082	58.43%	7,760	7,601	4,286
Year_28_103	4,787,945	4,775,977	3,126,068	65.45%	7,492	6,995	3,488
Year_28_104	3,800,007	3,790,349	2,158,316	56.94%	9,356	9,173	5,599
Year_28_105	5,078,185	5,062,593	2,725,577	53.84%	8,474	8,118	4,212
Year_28_106	3,885,240	3,874,503	2,109,253	54.44%	5,971	5,796	2,727
Year_28_107	3,750,161	3,738,972	2,234,371	59.76%	8,529	8,310	4,726
Year_28_108	4,079,409	4,068,661	2,227,769	54.75%	5,751	5,531	2,619
Year_28_109	4,676,485	4,665,690	2,475,377	53.05%	7,670	7,404	4,024
Year_28_110	3,656,587	3,645,978	331,881	9.10%	3,003	3,002	2,505
Year_28_111	5,080,186	5,056,436	2,718,542	53.76%	8,023	7,644	3,695
Year_28_112	5,291,412	5,268,139	3,000,352	56.95%	8,050	7,585	3,635
Year_28_113	6,032,126	6,000,624	3,562,181	59.36%	6,905	6,207	2,632
Year_28_114	6,771,907	6,703,833	233,196	3.48%	3,590	3,590	3,350
Year_28_115	5,510,764	5,482,992	3,254,434	59.36%	9,130	8,598	4,561
Year_28_116	7,087,187	7,066,684	4,036,631	57.12%	8,705	7,809	3,571
Year_28_117	6,541,216	6,524,005	3,648,573	55.93%	7,878	7,156	2,923
Year_28_118	6,775,181	6,747,070	3,691,696	54.72%	10,732	10,001	5,061
Year_28_119	4,439,670	4,428,589	2,519,861	56.90%	5,899	5,561	2,615
Year_28_120	4,519,993	4,508,802	2,524,393	55.99%	7,893	7,581	3,921
Year_28_121	3,935,295	3,925,715	2,337,872	59.55%	6,903	6,633	3,216
Year_28_122	3,683,011	3,674,282	2,089,449	56.87%	6,536	6,351	3,148
Year_28_123	3,397,077	3,387,348	1,877,320	55.42%	5,268	5,132	2,438
Year_28_124	4,025,545	4,015,433	2,448,770	60.98%	7,578	7,304	3,961

Year_28_126	22,793,457	22,719,386	14,714,078	64.76%	10,613	7,183	3,315
Year_28_127	21,654,700	21,588,512	12,898,615	59.75%	9,349	6,131	2,500
Year_28_128	17,360,660	17,312,477	8,992,635	51.94%	8,411	5,874	2,564
Year_28_129	16,457,164	16,411,499	9,326,320	56.83%	8,386	6,017	2,363
Year_28_130	17,101,317	17,053,093	9,106,046	53.40%	9,044	6,749	2,802
Year_28_131	16,708,378	16,661,053	10,349,038	62.12%	9,609	7,093	3,101
Year_28_132	23,399,623	23,316,083	2,481,305	10.64%	7,050	6,666	2,300
Year_28_133	17,877,898	17,826,125	10,171,855	57.06%	10,163	7,715	3,570
Year_28_134	16,337,235	16,293,760	9,012,548	55.31%	9,250	6,916	2,927
Year_28_135	15,033,051	14,995,214	8,263,255	55.11%	9,156	6,650	2,645
Year_28_137	4,129,281	4,120,178	2,535,893	61.55%	6,285	5,968	2,958
Year_28_138	4,146,846	4,136,321	2,242,404	54.21%	6,362	6,126	2,735
Year_28_139	2,937,901	2,930,495	1,715,288	58.53%	7,133	7,041	3,942
Year_28_140	4,383,977	4,373,499	2,443,138	55.86%	7,052	6,769	3,224

Supplementary table 5 Marker genes specific expressed in certain clusters of the hierarchical analysis

Cluster	Gene	qvalue
cluster A	RP11-685N3.1	3.96E-10
cluster A	TCL1A	5.46E-09
cluster A	PPIB	0.00420902
cluster A	ARPC1B	0.010717552
cluster A	TYROBP	0.011166651
cluster A	C7orf73	0.013115849
cluster A	PARP1	0.01797738
cluster A	COX5B	0.042623299
cluster B	PPIAL4C	1.91E-07
cluster B	PPIAL4G	1.97E-05
cluster B	CH17-132F21.1	0.000218526
cluster B	PPIAL4A	0.000218526
cluster B	COLEC11	0.000686868
cluster B	H3F3A	0.006778142
cluster B	PPIAL4D	0.009682089
cluster B	PFN1	0.03574297
cluster D	ARSI	5.42E-15
cluster D	MTRNR2L6	1.04E-12
cluster D	SLCO5A1	3.46E-12
cluster D	PDE3A	1.05E-11
cluster D	HFM1	3.08E-11
cluster D	UNC45B	6.18E-11
cluster D	LARP1B	1.06E-10
cluster D	DYNC2H1	4.06E-10
cluster D	MTRNR2L7	2.00E-09
cluster D	MEIS2	3.80E-09
cluster D	MAPK4	5.32E-09
cluster D	TACR1	1.62E-08
cluster D	PKNOX2	2.95E-08
cluster D	SNCA	2.95E-08
cluster D	MTRNR2L11	2.97E-08
cluster D	SORCS2	4.04E-08
cluster D	ERBB4	5.42E-08
cluster D	AKAP7	6.45E-07
cluster D	CHST12	7.21E-07
cluster D	DCC	8.59E-07
cluster D	PYY	1.60E-06
cluster D	MTRNR2L13	1.94E-06
cluster D	PDZRN4	2.07E-06

cluster D	RAE1	7.07E-06
cluster D	MTRNR2L4	7.33E-06
cluster D	FAM65C	7.33E-06
cluster D	PDGFA	9.81E-06
cluster D	PRRG3	2.37E-05
cluster D	KCNIP1	4.21E-05
cluster D	DIAPH2	0.000145969
cluster D	TXNL4A	0.000156599
cluster D	KIAA0825	0.000174327
cluster D	GNGT1	0.000267089
cluster D	PTPRQ	0.000303401
cluster D	C6orf58	0.000303401
cluster D	MRPS31	0.00038367
cluster D	CATSPERD	0.000394073
cluster D	PID1	0.000394092
cluster D	CTNNA2	0.000578848
cluster D	PIWIL1	0.000694771
cluster D	ANKDD1A	0.000719268
cluster D	KCNIP4	0.00073675
cluster D	MRPL41	0.001168342
cluster D	DLG2	0.001294919
cluster D	FLVCR1	0.001417708
cluster D	RPL28	0.001562205
cluster D	COL6A3	0.001769337
cluster D	STRIP2	0.001769337
cluster D	PCNXL4	0.003070216
cluster D	YTHDC2	0.003190062
cluster D	RPL39	0.003190062
cluster D	DNAH9	0.003307047
cluster D	TPT1	0.003606769
cluster D	PPEF2	0.00397514
cluster D	ERMP1	0.004195836
cluster D	GFM2	0.004195836
cluster D	MAP2	0.004195836
cluster D	ARHGAP22	0.005049966
cluster D	PPM1L	0.005049966
cluster D	STIP1	0.005409879
cluster D	BORA	0.006162519
cluster D	WDPCP	0.006750988
cluster D	DGKI	0.006750988
cluster D	SLC24A1	0.006750988
cluster D	CLNK	0.006750988

cluster D	RPL35	0.006924081
cluster D	TMEM117	0.007389227
cluster D	OPHN1	0.008601777
cluster D	GPC5	0.008747268
cluster D	MCC	0.010796324
cluster D	DHX36	0.011806722
cluster D	RPS21	0.012932622
cluster D	COMMD10	0.013691971
cluster D	CACNA1D	0.01420239
cluster D	KDM1B	0.014563631
cluster D	RPS2	0.015090336
cluster D	RNF152	0.015204567
cluster D	RSPO1	0.01657052
cluster D	RPTOR	0.018583366
cluster D	POLR3B	0.019863541
cluster D	ATP11B	0.019999666
cluster D	DOCK2	0.019999666
cluster D	SUFU	0.020161703
cluster D	ALPK1	0.020255901
cluster D	TCEANC2	0.020255901
cluster D	MAB21L3	0.020287543
cluster D	ACSL3	0.021234454
cluster D	H2AFV	0.023471163
cluster D	PHKB	0.023669386
cluster D	LGMN	0.023795813
cluster D	SLC41A1	0.024715499
cluster D	LRP1B	0.027252105
cluster D	CLASP1	0.027252105
cluster D	LUC7L3	0.027252105
cluster D	KIAA1328	0.031687279
cluster D	RPL37	0.031687279
cluster D	NTM	0.033317015
cluster D	PWWP2A	0.033862024
cluster D	MICAL1	0.034279318
cluster D	DEF6	0.035148089
cluster D	RSF1	0.036815628
cluster D	SPATS2L	0.038960147
cluster D	UBE2E1	0.038960147
cluster D	RPS15A	0.039667263
cluster D	NPAS3	0.044466065
cluster D	DDX54	0.044946078
cluster D	NFATC1	0.045628775

cluster D	VPS8	0.045628775
cluster E	CNKSR3	4.24E-05
cluster E	APOE	4.24E-05
cluster E	MYT1L	4.24E-05
cluster E	TMEM212	7.36E-05
cluster E	CTRC	7.36E-05
cluster E	C12orf50	0.000165475
cluster E	FGF5	0.000276481
cluster E	DPPA4	0.000276761
cluster E	ACSL6	0.000314757
cluster E	RBM34	0.000329505
cluster E	TIE1	0.000359872
cluster E	ZBTB16	0.000405302
cluster E	POMGNT1	0.000540019
cluster E	HSPA4L	0.000540019
cluster E	LINC00908	0.000662838
cluster E	ARMC10	0.000825868
cluster E	SPC25	0.000856902
cluster E	ZNF543	0.000911869
cluster E	RP11-47I22.3	0.001006275
cluster E	SOX13	0.001006275
cluster E	TRIM73	0.001302877
cluster E	SLC12A9	0.001324996
cluster E	SLC26A8	0.00135537
cluster E	RGSL1	0.001368871
cluster E	C12orf79	0.001388779
cluster E	VASH2	0.001388779
cluster E	PKD1L2	0.001388779
cluster E	AP3S2	0.001783988
cluster E	NMNAT1	0.001783988
cluster E	TONSL	0.001783988
cluster E	SETD2	0.001859821
cluster E	CEP104	0.001895634
cluster E	HES2	0.001895634
cluster E	SIX4	0.001895634
cluster E	GRK4	0.001940699
cluster E	SRGAP3	0.001940699
cluster E	STAP2	0.001940699
cluster E	FUT1	0.002001869
cluster E	CSNK1D	0.003252504
cluster E	CCDC142	0.00327414
cluster E	NEIL1	0.00327414

cluster E	ATP1A4	0.00327414
cluster E	LTBR	0.00388546
cluster E	FEZ1	0.004220613
cluster E	PYGL	0.004220613
cluster E	FCHSD2	0.004220613
cluster E	CPT1C	0.004485089
cluster E	PLEKHH3	0.004485089
cluster E	C4orf36	0.004485089
cluster E	IL17RD	0.004505884
cluster E	VPS13B	0.004505884
cluster E	EBPL	0.004537257
cluster E	XKR9	0.004649291
cluster E	AACS	0.004649291
cluster E	PIF1	0.004698687
cluster E	ZNF454	0.005087102
cluster E	MEMO1	0.005384029
cluster E	TMIGD2	0.005419474
cluster E	C14orf37	0.006147352
cluster E	CDHR4	0.006148358
cluster E	CDK10	0.006207855
cluster E	TTLL7	0.006521626
cluster E	BCL7A	0.006521626
cluster E	ATG2A	0.006806315
cluster E	PCDP1	0.006806315
cluster E	NDRG2	0.007091805
cluster E	GIPR	0.007091805
cluster E	HOXB7	0.007808051
cluster E	MPP4	0.008021891
cluster E	C12orf60	0.008021891
cluster E	ATP1A3	0.008021891
cluster E	PRR19	0.008021891
cluster E	WDR31	0.008045606
cluster E	TMEM180	0.008227155
cluster E	TMEM17	0.008946019
cluster E	PLCXD1	0.009639269
cluster E	SH2D3A	0.010874657
cluster E	TMEM45A	0.011477118
cluster E	KIF18B	0.012005439
cluster E	AAK1	0.012005439
cluster E	ABCB9	0.012005439
cluster E	SLC29A4	0.012005439
cluster E	ACOT11	0.012464066

cluster E	SAG	0.012464066
cluster E	SLC9A4	0.012464066
cluster E	PUM2	0.012464066
cluster E	VWCE	0.012464066
cluster E	KLHL35	0.012698604
cluster E	POLB	0.013323509
cluster E	GEMIN6	0.01348034
cluster E	DENND2A	0.01350739
cluster E	KANK2	0.01350739
cluster E	NPHS1	0.015235564
cluster E	GPR75-ASB3	0.015380029
cluster E	CYP3A43	0.017894053
cluster E	INTU	0.017894053
cluster E	MIPOL1	0.017894053
cluster E	CCDC125	0.018239129
cluster E	CCL16	0.018931905
cluster E	TRPV3	0.018931905
cluster E	PACS2	0.019101768
cluster E	PCYOX1	0.019101768
cluster E	SLC26A4	0.021314753
cluster E	RSPH3	0.021573813
cluster E	SHISA9	0.022935225
cluster E	FHL2	0.022935225
cluster E	SYP	0.025566634
cluster E	POU2F1	0.025566634
cluster E	PPIL6	0.025566634
cluster E	WDR83	0.025566634
cluster E	ZNF774	0.027040532
cluster E	PIWIL2	0.027437665
cluster E	AC008686.1	0.027677994
cluster E	PLEKHG1	0.027677994
cluster E	PSPH	0.027677994
cluster E	METTL2B	0.027677994
cluster E	LPIN1	0.028547228
cluster E	LRCH3	0.028547228
cluster E	C10orf82	0.028798509
cluster E	DNAH14	0.028798509
cluster E	LYN	0.028798509
cluster E	ADAMTS4	0.030472068
cluster E	SUN3	0.030669448
cluster E	TPM1	0.030669448
cluster E	DACT3	0.030902777

cluster E	SIMC1	0.030902777
cluster E	RALGPS1	0.030902777
cluster E	BEND7	0.030902777
cluster E	CMBL	0.031078199
cluster E	HAMP	0.031078199
cluster E	TPGS1	0.031890283
cluster E	C5AR2	0.032424156
cluster E	MCF2L2	0.032424156
cluster E	NUMA1	0.033065922
cluster E	ABCC9	0.03361594
cluster E	PNMA2	0.03361594
cluster E	MPP7	0.03361594
cluster E	SPIRE2	0.03361594
cluster E	SULT1B1	0.03361594
cluster E	CARD14	0.03361594
cluster E	FBXL18	0.03361594
cluster E	RDH13	0.03361594
cluster E	SGPP1	0.03361594
cluster E	ADRA1A	0.034116323
cluster E	EFCAB12	0.034918218
cluster E	CYHR1	0.034918218
cluster E	DDX17	0.035462431
cluster E	TTC39B	0.035462431
cluster E	COA7	0.035462431
cluster E	PSKH1	0.035462431
cluster E	C5AR1	0.035595634
cluster E	ECT2	0.035595634
cluster E	LIN7B	0.035595634
cluster E	PDDC1	0.03568073
cluster E	C14orf23	0.035838437
cluster E	COX6B2	0.035838437
cluster E	C12orf55	0.038164077
cluster E	ZNF620	0.038164077
cluster E	PLA2G4C	0.039115325
cluster E	PML	0.039115325
cluster E	ROR1	0.040225804
cluster E	CCDC150	0.040225804
cluster E	PCSK7	0.041296791
cluster E	C4orf32	0.041581012
cluster E	MAP3K12	0.042064354
cluster E	CENPL	0.042064354
cluster E	ATP13A3	0.043824097

cluster E	HSD17B13	0.043824097
cluster E	GDA	0.044200211
cluster E	C1orf61	0.048663215
cluster E	AC079354.1	0.048671264
cluster E	SPC24	0.048671264
cluster E	ULK2	0.048671264
cluster E	PPP1R13L	0.049294729
cluster E	TMPRSS2	0.049294729
cluster F	JUND	3.16E-14
cluster F	RGCC	1.06E-12
cluster F	KLF6	2.65E-12
cluster F	ZNF331	2.10E-09
cluster F	C16orf80	1.60E-08
cluster F	ID3	1.65E-07
cluster F	KLF4	3.62E-07
cluster F	ATF3	4.63E-07
cluster F	NAMPT	2.02E-06
cluster F	FOS	2.02E-06
cluster F	JUN	7.45E-06
cluster F	PTPRM	1.17E-05
cluster F	NR4A2	2.08E-05
cluster F	TRA2A	6.23E-05
cluster F	CCNL1	8.18E-05
cluster F	NFKBIA	8.24E-05
cluster F	RRP12	8.24E-05
cluster F	RAB11FIP1	0.000128842
cluster F	IER2	0.000128842
cluster F	MIDN	0.000128842
cluster F	MICAL3	0.000140122
cluster F	YPEL5	0.0003333
cluster F	SIK3	0.000564001
cluster F	ARL4A	0.000564001
cluster F	RASGEF1B	0.000574524
cluster F	NDRG4	0.000859615
cluster F	NUP98	0.000859615
cluster F	SAT1	0.000957567
cluster F	DUSP10	0.001177993
cluster F	GADD45B	0.001320521
cluster F	NUCKS1	0.001465402
cluster F	MAP3K8	0.002050594
cluster F	JMY	0.002134161
cluster F	RCBTB1	0.002332492

cluster F	DNAJA1	0.002373586
cluster F	TRA2B	0.002373586
cluster F	H3F3C	0.002373586
cluster F	KLF2	0.002909374
cluster F	GYLTL1B	0.002944017
cluster F	NAMPTL	0.002944017
cluster F	DDX24	0.002944017
cluster F	RGPD5	0.002944017
cluster F	TOR3A	0.003312757
cluster F	SLC2A14	0.00382652
cluster F	PAFAH1B1	0.004417141
cluster F	REL	0.004417141
cluster F	RGPD1	0.004417141
cluster F	RGPD6	0.004417141
cluster F	SON	0.004417141
cluster F	RP11-127H5.1	0.005286727
cluster F	ALG13	0.005286727
cluster F	CREM	0.005382583
cluster F	RPS6KA5	0.006156931
cluster F	RLF	0.007140765
cluster F	SFPQ	0.008167908
cluster F	PARP2	0.008167908
cluster F	TRIM62	0.008167908
cluster F	SORCS2	0.008716962
cluster F	LY9	0.008742874
cluster F	SRSF7	0.008847885
cluster F	JMJD1C	0.008972228
cluster F	PDCD4	0.008972228
cluster F	CCDC173	0.010754584
cluster F	CDKN1A	0.01095698
cluster F	TESK2	0.01095698
cluster F	NAALADL2	0.01095698
cluster F	CCRN4L	0.011035731
cluster F	EIF2AK3	0.011252951
cluster F	SLC3A2	0.011503675
cluster F	GIMAP2	0.012805701
cluster F	RGPD8	0.014229132
cluster F	DDIT3	0.01424568
cluster F	IZUMO2	0.014969262
cluster F	MASTL	0.014969262
cluster F	SLC2A3	0.015950045
cluster F	A2M	0.017984976

cluster F	ST18	0.018395597
cluster F	LUC7L	0.018395597
cluster F	RBM38	0.018395597
cluster F	ZNF775	0.019197304
cluster F	GSPT1	0.019197304
cluster F	IFRD1	0.021982416
cluster F	KDM6B	0.021982416
cluster F	PTP4A1	0.024178232
cluster F	STK17B	0.024184596
cluster F	NPIPB5	0.026000445
cluster F	RGPD3	0.026000445
cluster F	NPIPB3	0.027025602
cluster F	CD55	0.027692459
cluster F	SKIL	0.027715027
cluster F	RPS15A	0.027715027
cluster F	ZNF131	0.027715027
cluster F	CYCS	0.027715027
cluster F	PLK2	0.027715027
cluster F	CCDC174	0.028300814
cluster F	SELK	0.028300814
cluster F	AHI1	0.028300814
cluster F	DDX21	0.028300814
cluster F	RSBN1	0.028300814
cluster F	RGPD4	0.028403452
cluster F	ZNF165	0.032775802
cluster F	DDX3X	0.034791785
cluster F	BRIX1	0.038442878
cluster F	FBLN5	0.038442878
cluster F	EGR1	0.038458839
cluster F	GTF2E2	0.038458839
cluster F	MARCKS	0.038458839
cluster F	PPP1R15A	0.040784349
cluster F	ETV5	0.040784349
cluster F	TBC1D15	0.040784349
cluster F	BTAF1	0.046252389
cluster F	CREB5	0.04831975
cluster F	TUBA1A	0.04831975
cluster F	SRSF3	0.049324075
cluster F	SORBS2	0.04949026

Supplementary table 6 Pathway enrichemnts of the marker genes of the hierarchical analysis

Cluster	Gene_num_cluster	Gene_set	Description	Gene_num	p-value	FDR_q-value	Genes
clusterD	108	STRUCTURAL_CONSTITUENT_OF_RIBOSOME	Genes annotated by the GO term GO:0003735. The action of a molecule that contributes to the structural integrity of the ribosome.	8	8.86E-12	1.29E-08	RPL35,RPL28,RPL39,RPS2,RPS21,RPS15A,MRPL41,RPL37
clusterD	108	STRUCTURAL_MOLECULE_ACTIVITY	Genes annotated by the GO term GO:0005198. The action of a molecule that contributes to the structural integrity of a complex or assembly within or outside a cell.	8	6.36E-08	0.0000462	RPL35,RPL28,RPL39,RPS2,RPS21,RPS15A,MRPL41,RPL37
clusterD	108	RNA_BINDING	Genes annotated by the GO term GO:0003723. Interacting selectively with an RNA molecule or a portion thereof.	8	0.0000001	0.0000487	RPL35,RPL28,RPL39,RPS2,RPS21,RPS15A,RAE1,TXNL4A

clusterD	108	TRANSLATION	Genes annotated by the GO term GO:0006412. The chemical reactions and pathways resulting in the formation of a protein. This is a ribosome-mediated process in which the information in messenger RNA (mRNA) is used to specify the sequence of amino acids in the protein.	6	0.00000275	0.001	RPL35,RPL28,RPL39,RPS2,MRPL41,RPL37
clusterD	108	CELLULAR_PROTEIN_METABOLIC_PROCESS	Genes annotated by the GO term GO:0044267. The chemical reactions and pathways involving a specific protein, rather than of proteins in general, occurring at the level of an individual cell. Includes protein modification.	12	0.00000532	0.00119	RPL35,RPL28,RPL39,RPS2,MRPL41,RPL37,CHST12,CLASP1,SUFU,MAPK4,PPEF2,UBE2E1

clusterD	108	CELLULAR_MACROMOLECULE_METABOLIC_PROCESS	Genes annotated by the GO term GO:0044260. The chemical reactions and pathways involving macromolecules, large molecules including proteins, nucleic acids and carbohydrates, as carried out by individual cells.	12	0.00000603	0.00119	RPL35,RPL28,RPL39,RPS2,MRPL41,RPL37,CHST12,CLASP1,SUFU,MAPK4,PPEF2,UBE2E1
clusterD	108	CELLULAR_BIOSYNTHETIC_PROCESS	Genes annotated by the GO term GO:0044249. The chemical reactions and pathways resulting in the formation of substances, carried out by individual cells.	7	0.00000653	0.00119	RPL35,RPL28,RPL39,RPS2,MRPL41,RPL37,CHST12
clusterD	108	MACROMOLECULE_BIOSYNTHETIC_PROCESS	Genes annotated by the GO term GO:0009059. The chemical reactions and pathways resulting in the formation of macromolecules, large molecules including proteins, nucleic acids and carbohydrates.	7	0.00000653	0.00119	RPL35,RPL28,RPL39,RPS2,MRPL41,RPL37,CHST12

clusterD	108	PROTEIN_METABOLIC_PROCESS	Genes annotated by the GO term GO:0019538. The chemical reactions and pathways involving a specific protein, rather than of proteins in general. Includes protein modification.	12	0.000014	0.00227	RPL35,RPL28,RPL39,RPS2,MRPL41,RPL37,CHST12,CLASP1,SUFU,MAPK4,PPEF2,UBE2E1
clusterD	108	INTRACELLULAR_NON_MEMBRANE_BOUND_ORGANELLE	Genes annotated by the GO term GO:0043232. Organized structure of distinctive morphology and function, not bounded by a lipid bilayer membrane and occurring within the cell. Includes ribosomes, the cytoskeleton and chromosomes.	8	0.0000692	0.00898	RPL35,MRPL41,RAE1,CLASP1,DDX54,TPT1,MAP2,DNAH9
clusterD	108	NON_MEMBRANE_BOUND_ORGANELLE	Genes annotated by the GO term GO:0043228. Organized structure of distinctive morphology and function, not bounded by a lipid bilayer membrane.	8	0.0000692	0.00898	RPL35,MRPL41,RAE1,CLASP1,DDX54,TPT1,MAP2,DNAH9

			Includes ribosomes, the cytoskeleton and chromosomes.				
clusterD	108	BIOSYNTHETIC_PROCESS	Genes annotated by the GO term GO:0009058. The energy-requiring part of metabolism in which simpler substances are transformed into more complex ones, as in growth and other biosynthetic processes.	7	0.0000741	0.00898	RPL35,RPL28,RPL39,RPS2,MRPL41,RPL37,CHST12
clusterD	108	SIGNAL_TRANSDUCTION	Genes annotated by the GO term GO:0007165. The cascade of processes by which a signal interacts with a receptor, causing a change in the level or activity of a second messenger or other downstream	12	0.000209	0.0218	SUFU,DDX54,NFATC1,AKAP7,PYY,TACR1,KCNIP1,OPHN1,ERBB4,CLNK,PDGFA,MCC

			target, and ultimately effecting a change in the functioning of the cell.				
clusterD	108	MACROMOLECULAR_COMPLEX	Genes annotated by the GO term GO:0032991. A stable assembly of two or more macromolecules, i.e. proteins, nucleic acids, carbohydrates or lipids, in which the constituent parts function together.	9	0.00021	0.0218	MRPL41,RAE1,TXNL4A,CLASP1,TPT1,MAP2,RSF1,CACNA1D,SPATS2L
clusterD	108	INTRACELLULAR_ORGANELLE_PART	Genes annotated by the GO term GO:0044446. A constituent part of an intracellular organelle, an organized structure of distinctive morphology and function, occurring within the cell. Includes constituent parts of the nucleus, mitochondria, plastids, vacuoles, vesicles,	10	0.000257	0.0241	RPL35,MRPL41,RAE1,TXNL4A,CLASP1,DDX54,TPT1,MAP2,DNAH9,RSF1

			ribosomes and the cytoskeleton but excludes the plasma membrane.				
clusterD	108	ORGANELLE_PART	Genes annotated by the GO term GO:0044422. Any constituent part of an organelle, an organized structure of distinctive morphology and function. Includes constituent parts of the nucleus, mitochondria, plastids, vacuoles, vesicles, ribosomes and the cytoskeleton, but excludes the plasma membrane.	10	0.000265	0.0241	RPL35,MRPL41,RAE1,TXNL4A,CLASP1,DDX54,TPT1,MAP2,DNAH9,RSF1
clusterD	108	TRANSCRIPTION_REPRESSOR_ACTIVITY	Genes annotated by the GO term GO:0016564. Any transcription regulator	4	0.000339	0.029	SUFU,DDX54,RSF1,MEIS2

			activity that prevents or downregulates transcription.				
clusterF	115	BIOPOLYMER_METABOLIC_PROCESS	Genes annotated by the GO term GO:0043283. The chemical reactions and pathways involving biopolymers, long, repeating chains of monomers found in nature e.g. polysaccharides and proteins.	27	1.65E-15	3.52E-12	RPS6KA5,FOS,CCRN4L,SRSF7,TRA2B,TRA2A,CYCS,NUP98,JMY,DDIT3,JMJD1C,CREB5,JUND,KLF4,CREM,SKIL,GTF2E2,SFPQ,GSPT1,STK17B,TESK2,DUSP10,EIF2AK3,MAP3K8,SIK3,PTPRM,PARP2
clusterF	115	NUCLEOBASENUCLEOSIDENUCLEOTIDE_AND_NUCLEIC_ACID_METABOLIC_PROCESS	Genes annotated by the GO term GO:0006139. The chemical reactions and pathways involving nucleobases, nucleosides, nucleotides and nucleic acids.	22	1.45E-13	1.54E-10	RPS6KA5,FOS,CCRN4L,SRSF7,TRA2B,TRA2A,CYCS,NUP98,JMY,DDIT3,JMJD1C,CREB5,JUND,KLF4,CREM,SKIL,GTF2E2,SFPQ,GSPT1,EGR1,PDCD4,ID3

clusterF	115	NUCLEUS	Genes annotated by the GO term GO:0005634. A membrane-bounded organelle of eukaryotic cells in which chromosomes are housed and replicated. In most cells, the nucleus contains all of the cell's chromosomes except the organellar chromosomes, and is the site of RNA synthesis and processing. In some species, or in specialized cell types, RNA metabolism or DNA replication may be absent.	22	2.26E-12	1.61E-09	RPS6KA5,FOS,CCRN4L,SRSF7,TRA2B,TRA2A,CYCS,NUP98,STK17B,TESK2,DUSP10,EGR1,PDCD4,NFKBIA,JUN,DDX21,DDX3X,DDX24,SORBS2,ARL4A,NR4A2,KLF6
clusterF	115	RNA_METABOLIC_PROCESS	Genes annotated by the GO term GO:0016070. The chemical reactions and pathways involving RNA, ribonucleic acid, one of the two main type of nucleic acid, consisting of a long, unbranched	17	1.44E-11	7.66E-09	RPS6KA5,FOS,CCRN4L,SRSF7,TRA2B,TRA2A,JMY,DDIT3,JMJD1C,CREB5,JUND,KLF4,CREM,SKIL,GTF2E2,SFPQ,GSPT1

			macromolecule formed from ribonucleotides joined in 3',5'-phosphodiester linkage.				
clusterF	115	TRANSCRIPTION	Genes annotated by the GO term GO:0006350. The synthesis of either RNA on a template of DNA or DNA on a template of RNA.	15	3.07E-10	0.000000131	RPS6KA5,FOS,CCRN4L,JMY,DDIT3,JMJD1C,CREB5,JUND,KLF4,CREM,SKIL,GTF2E2,EGR1,PDCD4,ID3
clusterF	115	REGULATION_OF_CELLULAR_METABOLIC_PROCESS	Genes annotated by the GO term GO:0031323. Any process that modulates the frequency, rate or extent of the chemical reactions and pathways by which individual cells transform chemical substances.	14	5.27E-09	0.00000187	RPS6KA5,FOS,JMY,DDIT3,JMJD1C,CREB5,JUND,KLF4,CREM,EIF2AK3,EGR1,PDCD4,ID3,CDKN1A

clusterF	115	REGULATION_OF_METABOLIC_PROCESS	Genes annotated by the GO term GO:0019222. Any process that modulates the frequency, rate or extent of the chemical reactions and pathways within a cell or an organism.	14	6.38E-09	0.00000194	RPS6KA5,FOS,JMY,DDIT3,JMJD1C,CREB5,JUND,KLF4,CREM,EIF2AK3,EGR1,PDCD4,ID3,CDKN1A
clusterF	115	REGULATION_OF_GENE_EXPRESSION	Genes annotated by the GO term GO:0010468. Any process that modulates the frequency, rate or extent of gene expression. Gene expression is the process in which a gene's coding sequence is converted into a mature gene product or products (proteins or RNA). This includes the production of an RNA transcript as well as any processing to produce a mature RNA product or an mRNA (for protein-coding genes) and the translation of that	13	7.33E-09	0.00000195	RPS6KA5,FOS,JMY,DDIT3,JMJD1C,CREB5,JUND,KLF4,CREM,EIF2AK3,EGR1,PDCD4,ID3

			mRNA into protein. Some protein processing events may be included when they are required to form an active form of a product from an inactive precursor form.				
clusterF	115	REGULATION_OF_TRANSCRIPTION	Genes annotated by the GO term GO:0045449. Any process that modulates the frequency, rate or extent of the synthesis of either RNA on a template of DNA or DNA on a template of RNA.	12	1.04E-08	0.00000245	RPS6KA5,FOS,JMY,DDIT3,JMJD1C,CREB5,JUND,KLF4,CREM,EGR1,PDCD4,ID3

clusterF	115	REGULATION_OF_NUCLEOBASENUCLEOSIDENUCLEOTIDE_AND_NUCLEIC_ACID_METABOLIC_PROCESS	Genes annotated by the GO term GO:0019219. Any process that modulates the frequency, rate or extent of the chemical reactions and pathways involving nucleobases, nucleosides, nucleotides and nucleic acids.	12	2.71E-08	0.00000578	RPS6KA5,FOS,JMY,DDIT3,JMJD1C,CREB5,JUND,KLF4,CREM,EGR1,PDCD4,ID3
clusterF	115	TRANSCRIPTION_DNA_DEPENDENT	Genes annotated by the GO term GO:0006351. The synthesis of RNA on a template of DNA.	12	3.71E-08	0.00000681	RPS6KA5,FOS,CCRN4L,JMY,DDIT3,JMJD1C,CREB5,JUND,KLF4,CREM,SKIL,GTF2E2
clusterF	115	RNA_BIOSYNTHETIC_PROCESS	Genes annotated by the GO term GO:0032774. The chemical reactions and pathways resulting in the formation of RNA, ribonucleic acid, one of the two main type of nucleic acid, consisting of a long, unbranched macromolecule formed from ribonucleotides joined in 3',5'-	12	3.84E-08	0.00000681	RPS6KA5,FOS,CCRN4L,JMY,DDIT3,JMJD1C,CREB5,JUND,KLF4,CREM,SKIL,GTF2E2

			phosphodiester linkage. Includes polymerization of ribonucleotide monomers.				
clusterF	115	REGULATION_OF_TRANSCRIPTIONDNA_DEPENDENT	Genes annotated by the GO term GO:0006355. Any process that modulates the frequency, rate or extent of DNA-dependent transcription.	9	0.00000155	0.000253	RPS6KA5,FOS,JMY,DDIT3,JMJD1C,CREB5,JUND,KLF4,CREM
clusterF	115	MRNA_METABOLIC_PROCESS	Genes annotated by the GO term GO:0016071. The chemical reactions and pathways involving mRNA, messenger RNA, which is responsible for carrying the coded genetic 'message', transcribed from	5	0.00000174	0.000262	SRSF7,TRA2B,TRA2A,SFPQ,GSPT1

			DNA, to sites of protein assembly at the ribosomes.				
clusterF	115	REGULATION_OF_RNA_METABOLIC_PROCESS	Genes annotated by the GO term GO:0051252. Any process that modulates the frequency, rate or extent of the chemical reactions and pathways involving RNA.	9	0.00000185	0.000262	RPS6KA5,FOS,JMY,DDIT3,JMJD1C,CREB5,JUND,KLF4,CREM
clusterF	115	POST_TRANSLATIONAL_PROTEIN_MODIFICATION	Genes annotated by the GO term GO:0043687. The covalent alteration of one or more amino acids occurring in a protein after the protein has been completely translated and released from the ribosome.	9	0.00000201	0.000267	RPS6KA5,STK17B,TESK2,DUSP10,EIF2AK3,MAP3K8,SIK3,PTPRM,PARP2

clusterF	115	BIOPOLYMER_MODIFICATION	Genes annotated by the GO term GO:0043412. The covalent alteration of one or more monomeric units in a polypeptide, polynucleotide, polysaccharide, or other biological polymer, resulting in a change in its properties.	10	0.00000334	0.000418	RPS6KA5,FOS,STK17B,TESK2,DUSP10,EIF2AK3,MAP3K8,SIK3,PTPRM,PARP2
clusterF	115	TRANSCRIPTION_FACTOR_BINDING	Genes annotated by the GO term GO:0008134. Interacting selectively with a transcription factor, any protein required to initiate or regulate transcription.	7	0.00000869	0.000911	JMY,DDIT3,JMJD1C,SKIL,ID3,NFKBIA,ATF3

clusterF	115	APOPTOSIS_GO	Genes annotated by the GO term GO:0006915. A form of programmed cell death induced by external or internal signals that trigger the activity of proteolytic caspases, whose actions dismantle the cell and result in cell death. Apoptosis begins internally with condensation and subsequent fragmentation of the cell nucleus (blebbing) while the plasma membrane remains intact. Other characteristics of apoptosis include DNA fragmentation and the exposure of phosphatidyl serine on the cell surface.	8	0.00000871	0.000911	CYCS,JMY,STK17B,NFKBIA,CDKN1A,GADD45B,PPP1R15A,SON

clusterF	115	PROGRAMMED_CELL_DEATH	Genes annotated by the GO term GO:0012501. Cell death resulting from activation of endogenous cellular processes.	8	0.00000886	0.000911	CYCS,JMY,STK17B,NFKBIA,CDKN1A,GADD45B,PPP1R15A,SON
clusterF	115	CELL_DEVELOPMENT	Genes annotated by the GO term GO:0048468. The process whose specific outcome is the progression of the cell over time, from its formation to the mature structure. Cell development does not include the steps involved in committing a cell to a specific fate.	9	0.00000947	0.000911	CYCS,JMY,STK17B,NFKBIA,CDKN1A,GADD45B,PPP1R15A,SON,IFRD1
clusterF	115	PROTEIN_SERINE_THREONINE_KINASE_ACTIVITY	Genes annotated by the GO term GO:0004674. Catalysis of the reaction: ATP + a protein serine/threonine = ADP + protein serine/threonine phosphate.	6	0.0000096	0.000911	RPS6KA5,STK17B,TESK2,EIF2AK3,MAP3K8,SIK3

clusterF	115	PHOSPHORYLATION	Genes annotated by the GO term GO:0016310. The process of introducing a phosphate group into a molecule, usually with the formation of a phosphoric ester, a phosphoric anhydride or a phosphoric amide.	7	0.00000985	0.000911	RPS6KA5,STK17B,TESK2,EIF2AK3,MAP3K8,SIK3,CDKN1A
clusterF	115	TRANSCRIPTION_FROM_RNA_POLYMERASE_II_PROMOTER	Genes annotated by the GO term GO:0006366. The synthesis of RNA from a DNA template by RNA polymerase II (Pol II), originating at a Pol II-specific promoter. Includes transcription of messenger RNA (mRNA) and certain small nuclear RNAs (snRNAs).	8	0.0000133	0.00118	FOS,CCRN4L,JMY,DDIT3,CREB5,JUND,SKIL,GTF2E2

clusterF	115	PROTEIN_MODIFICATION_PROCESS	Genes annotated by the GO term GO:0006464. The covalent alteration of one or more amino acids occurring in proteins, peptides and nascent polypeptides (co-translational, post-translational modifications). Includes the modification of charged tRNAs that are destined to occur in a protein (pre-translation modification).	9	0.0000192	0.00163	RPS6KA5,STK17B,TESK2,DUSP10,EIF2AK3,MAP3K8,SIK3,PTPRM,PARP2
clusterF	115	TRANSCRIPTION_FACTOR_ACTIVITY	Genes annotated by the GO term GO:0003700. The function of binding to a specific DNA sequence in order to modulate transcription. The transcription factor may or may not also interact selectively with a protein or macromolecular complex.	7	0.0000217	0.00178	CCRN4L,CREB5,EGR1,JUN,ATF3,RLF,ETV5

clusterF	115	REACTOME_NFKB_AND_MAP_KINASES_ACTIVATION_MEDIATED_BY_TLR4_SIGNALING_REPERTOIRE	Genes involved in NFkB and MAP kinases activation mediated by TLR4 signaling repertoire	4	0.0000262	0.00207	RPS6KA5,FOS,NFKBIA,JUN
clusterF	115	MRNA_PROCESSING_GO_0006397	Genes annotated by the GO term GO:0006397. Any process involved in the conversion of a primary mRNA transcript into one or more mature mRNA(s) prior to translation into polypeptide.	4	0.0000277	0.00207	SRSF7,TRA2B,TRA2A,SFPQ
clusterF	115	RESPONSE_TO_STRESS	Genes annotated by the GO term GO:0006950. A change in state or activity of a cell or an organism (in terms of movement, secretion, enzyme production, gene expression, etc.) as a result of a stimulus indicating the organism is under	8	0.0000283	0.00207	RPS6KA5,FOS,DDIT3,DUSP10,EIF2AK3,GADD45B,PPP1R15A,DNAJA1

			stress. The stress is usually, but not necessarily, exogenous (e.g. temperature, humidity, ionizing radiation).				
clusterF	115	REACTOME_TRIF_MEDIATED_TLR3_SIGNALING	Genes involved in TRIF mediated TLR3 signaling	4	0.0000292	0.00207	RPS6KA5,FOS,NFKBIA,JUN
clusterF	115	TRANSCRIPTION_REPRESSOR_ACTIVITY	Genes annotated by the GO term GO:0016564. Any transcription regulator activity that prevents or downregulates transcription.	5	0.0000314	0.00216	DDIT3,KLF4,SKIL,ID3,ATF3
clusterF	115	REACTOME_TRAF6_MEDIATED_INDUCTION_OF_NFKB_AND_MAP_KINASES_UPON_TLR7_8_OR_9_ACTIVATION	Genes involved in TRAF6 mediated induction of NFkB and MAP kinases upon TLR7/8 or 9 activation	4	0.0000342	0.00227	RPS6KA5,FOS,NFKBIA,JUN

clusterF	115	REACTOME_PERK_REGULATED_GENE_EXPRESSION	Genes involved in PERK regulated gene expression	3	0.0000441	0.00284	DDIT3,EIF2AK3,ATF3
clusterF	115	REACTOME_MYD88_MAL_CASCADE_INITIATED_ON_PLASMA_MEMBRANE	Genes involved in MyD88:Mal cascade initiated on plasma membrane	4	0.0000459	0.00288	RPS6KA5,FOS,NFKBIA,JUN
clusterF	115	REACTOME_MAPK_TARGETS_NUCLEAR_EVENTS_MEDIATED_BY_MAP_KINASES	Genes involved in MAPK targets/ Nuclear events mediated by MAP kinases	3	0.0000489	0.00297	RPS6KA5,FOS,JUN
clusterF	115	PROTEIN_AMINO_ACID_PHOSPHORYLATION	Genes annotated by the GO term GO:0006468. The process of introducing a phosphate group on to a protein.	6	0.0000541	0.0032	RPS6KA5,STK17B,TESK2,EIF2AK3,MAP3K8,SIK3

clusterF	115	PROTEIN_KINASE_ACTIVITY	Genes annotated by the GO term GO:0004672. Catalysis of the phosphorylation of an amino acid residue in a protein, usually according to the reaction: a protein + ATP = a phosphoprotein + ADP.	6	0.0000608	0.0035	RPS6KA5,STK17B,TESK2,EIF2AK3,MAP3K8,SIK3
clusterF	115	RNA_SPLICING	Genes annotated by the GO term GO:0008380. The process of removing sections of the primary RNA transcript to remove sequences not present in the mature form of the RNA and joining the remaining sections to form the mature form of the RNA.	4	0.0000659	0.00369	SRSF7,TRA2B,TRA2A,SFPQ
clusterF	115	PROTEIN_KINASE_CASCADE	Genes annotated by the GO term GO:0007243. A series of reactions, mediated by protein kinases, which occurs	6	0.0000708	0.00381	RPS6KA5,STK17B,DUSP10,GADD45B,REL,PLK2

			as a result of a single trigger reaction or compound.				
clusterF	115	REACTOME_ACTIVATED_TLR4_SIGNALLING	Genes involved in Activated TLR4 signalling	4	0.0000717	0.00381	RPS6KA5,FOS,NFKBIA,JUN
clusterF	115	TRANSCRIPTION_COREPRESSOR_ACTIVITY	Genes annotated by the GO term GO:0003714. The function of a transcription cofactor that represses transcription from a RNA polymerase II promoter; does not bind DNA itself.	4	0.0000747	0.00388	DDIT3,SKIL,ID3,ATF3
clusterF	115	DNA_BINDING	Genes annotated by the GO term GO:0003677. Interacting selectively with DNA (deoxyribonucleic acid).	8	0.0000925	0.00469	CCRN4L,CREB5,EGR1,JUN,ATF3,SON,RLF,ETV5
clusterF	115	PHOSPHOTRANSFERASE_ACTIVITY_ALCOHOL_GROUP_AS_ACCEPTOR	Genes annotated by the GO term GO:0016773. Catalysis of the transfer of a phosphorus-containing group from one	6	0.000145	0.00716	RPS6KA5,STK17B,TESK2,EIF2AK3,MAP3K8,SIK3

			compound (donor) to an alcohol group (acceptor).				
clusterF	115	NEGATIVE_REGULATION_OF_CELLULAR_PROCESS	Genes annotated by the GO term GO:0048523. Any process that stops, prevents or reduces the frequency, rate or extent of cellular processes, those that are carried out at the cellular level, but are not necessarily restricted to a single cell. For example, cell communication occurs among more than one cell, but occurs at the cellular level.	8	0.00015	0.00725	JMY,KLF4,EIF2AK3,PDCD4,ID3,CDKN1A,PPP1R15A,SON
clusterF	115	REACTOME_TOLL_RECEPTOR_CASCADES	Genes involved in Toll Receptor Cascades	4	0.00018	0.00851	RPS6KA5,FOS,NFKBIA,JUN

clusterF	115	INTRACELLULAR_SIGNALING_CASCADE	Genes annotated by the GO term GO:0007242. A series of reactions within the cell that occur as a result of a single trigger reaction or compound.	8	0.000186	0.00851	RPS6KA5,CYCS,STK17B,DUSP10,EIF2AK3,GADD45B,REL,PLK2
clusterF	115	POSITIVE_REGULATION_OF_CELLULAR_PROCESS	Genes annotated by the GO term GO:0048522. Any process that activates or increases the frequency, rate or extent of cellular processes, those that are carried out at the cellular level, but are not necessarily restricted to a single cell. For example, cell communication occurs among more than one cell, but occurs at the cellular level.	8	0.000188	0.00851	JMY,CREB5,STK17B,EGR1,CDKN1A,REL,PLK2,NAMPT

clusterF	115	NEGATIVE_REGULATION_OF_BIOLOGICAL_PROCESS	Genes annotated by the GO term GO:0048519. Any process that stops, prevents or reduces the frequency, rate or extent of a biological process. Biological processes are regulated by many means; examples include the control of gene expression, protein modification or interaction with a protein or substrate molecule.	8	0.000206	0.00912	JMY,KLF4,EIF2AK3,PDCD4,ID3,CDKN1A,PPP1R15A,SON
clusterF	115	TRANSCRIPTION_COFACTOR_ACTIVITY	Genes annotated by the GO term GO:0003712. The function that links a sequence-specific transcription factor to the core RNA polymerase II complex but does not bind DNA itself.	5	0.000212	0.00919	JMY,DDIT3,SKIL,ID3,ATF3

clusterF	115	REACTOME_MAP_KINASE_ACTIVATION_IN_TLR_CASCADE	Genes involved in MAP kinase activation in TLR cascade	3	0.000228	0.00971	RPS6KA5,FOS,JUN
clusterF	115	REACTOME_ACTIVATION_OF_THE_AP1_FAMILY_OF_TRANSCRIPTION_FACTORS	Genes involved in Activation of the AP-1 family of transcription factors	2	0.000243	0.0101	FOS,JUN
clusterF	115	KINASE_ACTIVITY	Genes annotated by the GO term GO:0016301. Catalysis of the transfer of a phosphate group, usually from ATP, to a substrate molecule.	6	0.000247	0.0101	RPS6KA5,STK17B,TESK2,EIF2AK3,MAP3K8,SIK3
clusterF	115	POSITIVE_REGULATION_OF_BIOLOGICAL_PROCESS	Genes annotated by the GO term GO:0048518. Any process that activates or increases the frequency, rate or extent of a biological process. Biological processes are regulated by many means; examples	8	0.000281	0.0112	JMY,CREB5,STK17B,EGR1,CDKN1A,REL,PLK2,NAMPT

			include the control of gene expression, protein modification or interaction with a protein or substrate molecule.				
clusterF	115	REACTOME_DIABETES_PATHWAYS	Genes involved in Diabetes pathways	4	0.000285	0.0112	DDIT3,KLF4,EIF2AK3,ATF3
clusterF	115	CELLULAR_PROTEIN_METABOLIC_PROCESS	Genes annotated by the GO term GO:0044267. The chemical reactions and pathways involving a specific protein, rather than of proteins in general, occurring at the level of an individual cell. Includes protein modification.	10	0.000312	0.0121	RPS6KA5,STK17B,TESK2,DUSP10,EIF2AK3,MAP3K8,SIK3,PTPRM,PARP2,DNAJA1
clusterF	115	CELLULAR_MACROMOLECULE_METABOLIC_PROCESS	Genes annotated by the GO term GO:0044260. The chemical reactions and pathways involving macromolecules, large	10	0.000344	0.0131	RPS6KA5,STK17B,TESK2,DUSP10,EIF2AK3,MAP3K8,SIK3,PTPRM,PARP2,DNAJA1

			molecules including proteins, nucleic acids and carbohydrates, as carried out by individual cells.				
clusterF	115	REACTOME_IMMUNE_SYSTEM	Genes involved in Immune System	9	0.000368	0.0137	RPS6KA5,FOS,MAP3K8,EGR1,NFKBIA,JUN,CDKN1A,REL,CD55
clusterF	115	NEGATIVE_REGULATION_OF_CELLULAR_METABOLIC_PROCESS	Genes annotated by the GO term GO:0031324. Any process that stops, prevents or reduces the frequency, rate or extent of the chemical reactions and pathways by which individual cells transform chemical substances.	5	0.00038	0.0139	KLF4,EIF2AK3,PDCD4,ID3,CDKN1A
clusterF	115	NEGATIVE_REGULATION_OF_METABOLIC_PROCESS	Genes annotated by the GO term GO:0009892. Any process that stops, prevents or reduces the frequency, rate or	5	0.000401	0.0144	KLF4,EIF2AK3,PDCD4,ID3,CDKN1A

			extent of the chemical reactions and pathways within a cell or an organism.				
clusterF	115	CELL_FATE_COMMITMENT	Genes annotated by the GO term GO:0045165. The commitment of cells to specific cell fates and their capacity to differentiate into particular kinds of cells. Positional information is established through protein signals that emanate from a localized source within a cell (the initial one-cell zygote) or within a developmental field.	2	0.00042	0.0149	KLF4,IFRD1
clusterF	115	SIGNAL_TRANSDUCTION	Genes annotated by the GO term GO:0007165. The cascade of processes by which a signal interacts with a receptor, causing a change in the level or activity of	12	0.00047	0.0164	RPS6KA5,CYCS,CREM,SKIL,STK17B,DUSP10,EIF2AK3,GADD45B,REL,PLK2,NAMPT,PAFAH1B1

			a second messenger or other downstream target, and ultimately effecting a change in the functioning of the cell.				
clusterF	115	TRANSFERASE_ACTIVITY_TRANSFERRING_PHOSPHORUS_CONTAINING_GROUPS	Genes annotated by the GO term GO:0016772. Catalysis of the transfer of a phosphorus-containing group from one compound (donor) to another (acceptor).	6	0.000516	0.0177	RPS6KA5,STK17B,TESK2,EIF2AK3,MAP3K8,SIK3
clusterF	115	REACTOME_INNATE_IMMUNE_SYSTEM	Genes involved in Innate Immune System	5	0.000533	0.018	RPS6KA5,FOS,NFKBIA,JUN,CD55
clusterF	115	PROTEIN_METABOLIC_PROCESS	Genes annotated by the GO term GO:0019538. The chemical reactions and pathways involving a specific protein, rather than of proteins in general. Includes protein modification.	10	0.000663	0.0221	RPS6KA5,STK17B,TESK2,DUSP10,EIF2AK3,MAP3K8,SIK3,PTPRM,PARP2,DNAJA1

clusterF	115	ATP_DEPENDENT_RNA_HELICASE_ACTIVITY	Genes annotated by the GO term GO:0004004. Catalysis of the reaction: ATP + H2O = ADP + phosphate, driving the unwinding of an RNA helix.	2	0.000727	0.0238	DDX21,DDX3X
clusterF	115	INTRACELLULAR_NON_MEMBRANE_BOUND_ORGANELLE	Genes annotated by the GO term GO:0043232. Organized structure of distinctive morphology and function, not bounded by a lipid bilayer membrane and occurring within the cell. Includes ribosomes, the cytoskeleton and chromosomes.	7	0.000751	0.0238	JUND,JUN,DDX21,DDX24,SORBS2,PAFAH1B1,MARCKS
clusterF	115	NON_MEMBRANE_BOUND_ORGANELLE	Genes annotated by the GO term GO:0043228. Organized structure of distinctive morphology and function, not bounded by a lipid bilayer membrane.	7	0.000751	0.0238	JUND,JUN,DDX21,DDX24,SORBS2,PAFAH1B1,MARCKS

			Includes ribosomes, the cytoskeleton and chromosomes.				
clusterF	115	RNA_PROCESSING	Genes annotated by the GO term GO:0006396. Any process involved in the conversion of one or more primary RNA transcripts into one or more mature RNA molecules.	4	0.000766	0.024	SRSF7,TRA2B,TRA2A,SFPQ
clusterF	115	RNA_DEPENDENT_ATPASE_ACTIVITY	Genes annotated by the GO term GO:0008186. Catalysis of the reaction: ATP + H2O = ADP + phosphate, in the presence of RNA; drives another reaction.	2	0.000817	0.0252	DDX21,DDX3X
clusterF	115	REACTOME_UNFOLDED_PROTEIN_RESPONSE	Genes involved in Unfolded Protein Response	3	0.000909	0.0271	DDIT3,EIF2AK3,ATF3

clusterF	115	CELLULAR_RESPONSE_TO_STIMULUS	Genes annotated by the GO term GO:0051716. A change in state or activity of a cell (in terms of movement, secretion, enzyme production, gene expression, etc.) as a result of a stimulus.	2	0.000912	0.0271	EIF2AK3,CDKN1A
clusterF	115	CELL_CYCLE_GO_0007049	Genes annotated by the GO term GO:0007049. The progression of biochemical and morphological phases and events that occur in a cell during successive cell replication or nuclear replication events. Canonically, the cell cycle comprises the replication and segregation of genetic material followed by the division of the cell, but in endocycles or syncytial cells nuclear	5	0.000918	0.0271	JMY,GSPT1,CDKN1A,PPP1R15A,PAFAH1B1

			replication or nuclear division may not be followed by cell division.				
clusterF	115	REGULATION_OF_MOLECULAR_FUNCTION	Genes annotated by the GO term GO:0065009. Any process that modulates the frequency, rate or extent of molecular functions. Molecular functions are elemental biological activities occurring at the molecular level, such as catalysis or binding.	5	0.00104	0.0303	CYCS,JMY,PDCD4,ID3,GADD45B
clusterF	115	REGULATION_OF_APOPTOSIS	Genes annotated by the GO term GO:0042981. Any process that modulates	5	0.0013	0.0375	CYCS,JMY,STK17B,CDKN1A,SON

			the occurrence or rate of cell death by apoptosis.				
clusterF	115	REGULATION_OF_PROGRAMMED_CELL_DEATH	Genes annotated by the GO term GO:0043067. Any process that modulates the frequency, rate or extent of programmed cell death, cell death resulting from activation of endogenous cellular processes.	5	0.00132	0.0375	CYCS,JMY,STK17B,CDKN1A,SON
clusterF	115	RNA_HELICASE_ACTIVITY	Genes annotated by the GO term GO:0003724. Catalysis of the unwinding of an RNA helix.	2	0.00146	0.0409	DDX21,DDX3X
clusterF	115	REACTOME_DOWNSTREAM_SIGNALING_EVENTS_OF_B_CELL_RECEPTOR_BCR	Genes involved in Downstream Signaling Events Of B Cell Receptor (BCR)	3	0.00159	0.0438	NFKBIA,CDKN1A,REL

clusterF	115	NUCLEOSIDE_TRIPHOSPHATASE_ACTIVITY	Genes annotated by the GO term GO:0017111. Catalysis of the reaction: a nucleoside triphosphate + H2O = nucleoside diphosphate + phosphate.	4	0.00162	0.0443	GSPT1,DDX21,DDX3X,ARL4A
clusterF	115	REACTOME_ACTIVATION_OF_GENES_BY_ATF4	Genes involved in Activation of Genes by ATF4	2	0.00171	0.0462	DDIT3,ATF3
clusterF	115	ATP_DEPENDENT_HELICASE_ACTIVITY	Genes annotated by the GO term GO:0008026. Catalysis of the reaction: ATP + H2O = ADP + phosphate to drive the unwinding of a DNA or RNA helix.	2	0.00185	0.0486	DDX21,DDX3X
clusterF	115	G1_S_TRANSITION_OF_MITOTIC_CELL_CYCLE	Genes annotated by the GO term GO:0000082. Progression from G1 phase to S phase of the mitotic cell cycle.	2	1.85E-03	4.86E-02	GSPT1,CDKN1A

Supplementary table 7 Pathway enrichemnt of the genes in certain Monocle clusters

Cluster	Description	qvalue	geneID
1	Retinoid metabolism and transport	0.062	AKR1B10/APOE/BCO2/HSPG2
1	Regulation of Complement cascade	0.062	C2/C3/C5/CFI
1	Complement cascade	0.062	C2/C3/C5/CFI
1	Peptide ligand-binding receptors	0.062	C3/C5/CCL16/CCL28/CXCL16
1	Class A/1 (Rhodopsin-like receptors)	0.075	C3/C5/CCL16/CCL28/CNR2/CXCL16/CYSLTR1
1	G alpha (i) signalling events	0.113	ADCY2/C3/C5/CCL16/CCL28/CNR2/CXCL16
1	Effects of PIP2 hydrolysis	0.165	DAGLB/DGKB/DGKE/DGKZ
1	Cell surface interactions at the vascular wall	0.165	CXADR/GLG1/ITGA3/ITGAL/ITGAX/LCK/YES1
1	Immunoregulatory interactions between a Lymphoid and a non-Lymphoid cell	0.165	C3/CD19/CXADR/ITGAL/KLRD1
1	Visual phototransduction	0.165	AKR1B10/APOE/BCO2/HSPG2/SAG
1	Diseases associated with visual transduction	0.165	AKR1B10/APOE/BCO2/HSPG2/SAG
2	Class B/2 (Secretin family receptors)	0.064	CD97/GNAS/GNB5/GNG2/PTH2R/WNT3
3	Signaling by FGFR in disease	0.006	AKT3/CAMK4/CBL/CUX1/FRS2/ITPR2/KRAS/MAP2K2/MDM2/MKNK1/NRG4/RICTOR/SOS1/STAT1/ZMYM2
3	Signaling by FGFR mutants	0.025	CUX1/FRS2/KRAS/SOS1/STAT1/ZMYM2
3	Signaling by FGFR	0.025	AKT3/CAMK4/CBL/FRS2/ITPR2/KRAS/MAP2K2/MDM2/MKNK1/NRG4/RICTOR/SOS1
3	Signaling by the B Cell Receptor (BCR)	0.025	AKT3/BTRC/CARD11/CBL/FRS2/ITPR2/KRAS/MDM2/NFKBIB/NRG4/PSMC6/PSMD5/RICTOR/SOS1/STIM1
3	Fc epsilon receptor (FCERI) signaling	0.031	AKT3/CARD11/FRS2/ITPR2/KRAS/MAP2K2/MAP2K4/MDM2/NRG4/PPP3CB/RICTOR/SOS1/VAV3
3	GRB2 events in ERBB2 signaling	0.031	KRAS/MAP2K2/NRG4/SOS1
3	Signaling by EGFR	0.031	ADAM10/AKT3/CAMK4/CBL/FRS2/ITPR2/KRAS/MAP2K2/MDM2/NRG4/RICTOR/SOS1
3	SHC1 events in ERBB2 signaling	0.031	KRAS/MAP2K2/NRG4/SOS1
3	SHC1 events in ERBB4 signaling	0.031	KRAS/MAP2K2/NRG4/SOS1
3	Signaling by EGFR in Cancer	0.031	ADAM10/AKT3/CAMK4/CBL/FRS2/ITPR2/KRAS/MAP2K2/MDM2/NRG4/RICTOR/SOS1

3	DAP12 signaling	0.031	AKT3/CAMK4/FRS2/ITPR2/KRAS/MAP2K2/MDM2/NRG4/RICTOR/SOS1/VAV3
3	FRS2-mediated cascade	0.031	FRS2/KRAS/MAP2K2/SOS1
3	Downstream signal transduction	0.031	AKT3/CAMK4/FRS2/ITPR2/KRAS/MAP2K2/MDM2/NRG4/RICTOR/SOS1/STAT1
3	Signaling by SCF-KIT	0.031	AKT3/CBL/FRS2/KRAS/MAP2K2/MDM2/NRG4/RICTOR/SOS1/STAT1
3	DAP12 interactions	0.031	AKT3/CAMK4/FRS2/ITPR2/KRAS/MAP2K2/MDM2/NRG4/RICTOR/SOS1/VAV3
3	Signaling by ERBB4	0.033	AKT3/BTRC/FRS2/ITCH/KRAS/MAP2K2/MDM2/NRG4/RICTOR/SOS1
3	Downstream signaling of activated FGFR	0.034	AKT3/CAMK4/FRS2/ITPR2/KRAS/MAP2K2/MDM2/NRG4/RICTOR/SOS1
3	Signalling to ERKs	0.041	FRS2/KRAS/MAP2K2/MAPK14/SOS1
3	NCAM signaling for neurite out-growth	0.046	CACNB2/KRAS/MAP2K2/SOS1/SPTAN1
3	Signalling to RAS	0.048	KRAS/MAP2K2/MAPK14/SOS1
3	FCERI mediated MAPK activation	0.048	KRAS/MAP2K2/MAP2K4/SOS1/VAV3
3	Signaling by PDGF	0.048	AKT3/CAMK4/FRS2/ITPR2/KRAS/MAP2K2/MDM2/NRG4/RICTOR/SOS1/STAT1
3	Signaling by ERBB2	0.048	AKT3/CAMK4/FRS2/ITPR2/KRAS/MAP2K2/MDM2/NRG4/RICTOR/SOS1
3	Downstream signaling events of B Cell Receptor (BCR)	0.055	AKT3/BTRC/CARD11/FRS2/KRAS/MDM2/NFKBIB/NRG4/PSMC6/PSMD5/RICTOR
3	Translocation of GLUT4 to the plasma membrane	0.082	EXOC4/EXOC6/LNPEP/RALGAPB/STX4/TBC1D1
3	ISG15 antiviral mechanism	0.084	EIF4E/KPNA1/KPNA4/NUP188/NUP35/NUP85/STAT1
3	Antiviral mechanism by IFN-stimulated genes	0.084	EIF4E/KPNA1/KPNA4/NUP188/NUP35/NUP85/STAT1
3	NGF signalling via TRKA from the plasma membrane	0.098	AKT3/CAMK4/FRS2/ITPR2/KRAS/MAP2K2/MAPK14/MDM2/NRG4/RICTOR/SOS1
3	FCERI mediated Ca+2 mobilization	0.098	ITPR2/PPP3CB/SOS1/VAV3
3	Platelet homeostasis	0.121	ATP2A2/ITPR2/MAPK14/PPP2R5E/STIM1

3	Innate Immune System	0.123	AKT3/CAMK4/CARD11/DHX9/FRS2/ITCH/ITPR2/KRAS/MAP2K2/MAP2K3/MAP2K4/MAPK14/MDM2/NFKBIB/NRG4/PLD3/PPP3CB/PRKDC/RICTOR/SOS1/TRIM56/VAV3
3	Antigen activates B Cell Receptor (BCR) leading to generation of second messengers	0.131	CBL/ITPR2/SOS1/STIM1
3	Immune System	0.131	AKT3/ANAPC7/BTRC/CAMK4/CARD11/CBL/DHX9/EIF4E/FBXW8/FRS2/ITCH/ITPR2/KPNA1/KPNA4/KRAS/LNPEP/MAP2K2/MAP2K3/MAP2K4/MAPK14/MDM2/NFKBIB/NRG4/NUP188/NUP35/NUP85/PLD3/PPP2R5E/PPP3CB/PRKDC/PSMC6/PSMD5/PTK2B/RICTOR/RNF4/SOS1/STAT1/STIM1/TRIM56/UBE2G1/UBE2V2/UBE3C/VAV3
3	Signaling by Interleukins	0.152	BTRC/CBL/KRAS/MAP2K2/MAP2K4/PTK2B/SOS1/STAT1
3	Constitutive PI3K/AKT Signaling in Cancer	0.152	AKT3/FRS2/MDM2/NRG4/RICTOR
3	Vpr-mediated nuclear import of PICs	0.155	KPNA1/NUP188/NUP35/NUP85
3	Nuclear Pore Complex (NPC) Disassembly	0.155	NEK7/NUP188/NUP35/NUP85
3	Glucose transport	0.155	HK1/NUP188/NUP35/NUP85
3	Hexose transport	0.163	HK1/NUP188/NUP35/NUP85
3	Interleukin-2 signaling	0.163	KRAS/MAP2K2/PTK2B/SOS1
3	Cytokine Signaling in Immune system	0.163	BTRC/CBL/EIF4E/KPNA1/KPNA4/KRAS/MAP2K2/MAP2K4/NUP188/NUP35/NUP85/PTK2B/SOS1/STAT1
3	Disease	0.165	ADAM10/AKT3/ATP6V0D1/BTRC/CAMK4/CBL/CHST15/CUX1/DOCK2/FRS2/GOT2/HK1/ITCH/ITPR2/KPNA1/KRAS/MAP2K2/MDM2/MKNK1/MTR/NRG4/NUMB/NUP188/NUP35/NUP85/PANK2/PSMC6/PSMD5/RICTOR/SOS1/ST3GAL3/STAT1/TAF1/TBP/TFDP2/TPK1/USP15/ZMYM2
3	Role of LAT2/NTAL/LAB on calcium mobilization	0.166	AKT3/FRS2/MDM2/NRG4/RICTOR/SOS1
3	Interactions of Vpr with host cellular proteins	0.168	KPNA1/NUP188/NUP35/NUP85
3	IRS-mediated signalling	0.168	EIF4E/FRS2/KRAS/MAP2K2/SOS1
3	IRS-related events triggered by IGF1R	0.168	EIF4E/FRS2/KRAS/MAP2K2/SOS1
3	IRS-related events	0.168	EIF4E/FRS2/KRAS/MAP2K2/SOS1
3	Developmental Biology	0.168	AKT3/ANK3/CACNB2/CAP2/DPYSL2/KRAS/MAP2K2/MAPK14/MED13/MED17/NCOA2/NUMB/SOS1/SPTAN1

3	TRAF6 Mediated Induction of proinflammatory cytokines	0.168	MAP2K2/MAP2K3/MAP2K4/MAPK14/NFKBIB
3	Signaling by Type 1 Insulin-like Growth Factor 1 Receptor (IGF1R)	0.168	EIF4E/FRS2/KRAS/MAP2K2/SOS1
3	IGF1R signaling cascade	0.168	EIF4E/FRS2/KRAS/MAP2K2/SOS1
3	Signaling by Insulin receptor	0.173	ATP6V0D1/EIF4E/FRS2/KRAS/MAP2K2/SOS1
3	Insulin receptor signalling cascade	0.173	EIF4E/FRS2/KRAS/MAP2K2/SOS1
3	L1CAM interactions	0.188	ANK3/DPYSL2/MAP2K2/NUMB/SPTAN1
3	Activation of NF-kappaB in B cells	0.188	BTRC/CARD11/NFKBIB/PSMC6/PSMD5
3	PI3K events in ERBB4 signaling	0.188	AKT3/FRS2/MDM2/NRG4/RICTOR
3	PIP3 activates AKT signaling	0.188	AKT3/FRS2/MDM2/NRG4/RICTOR
3	PI-3K cascade	0.188	AKT3/FRS2/MDM2/NRG4/RICTOR
3	PI3K events in ERBB2 signaling	0.188	AKT3/FRS2/MDM2/NRG4/RICTOR
3	PI3K/AKT Signaling in Cancer	0.188	AKT3/FRS2/MDM2/NRG4/RICTOR
3	Signalling by NGF	0.188	AKT3/CAMK4/FRS2/ITPR2/KRAS/MAP2K2/MAPK14/MDM2/NRG4/RICTOR/SOS1/VAV3
3	Toll Like Receptor 10 (TLR10) Cascade	0.188	MAP2K2/MAP2K3/MAP2K4/MAPK14/NFKBIB
3	Toll Like Receptor 5 (TLR5) Cascade	0.188	MAP2K2/MAP2K3/MAP2K4/MAPK14/NFKBIB
3	GAB1 signalosome	0.188	AKT3/FRS2/MDM2/NRG4/RICTOR
3	PI3K/AKT activation	0.188	AKT3/FRS2/MDM2/NRG4/RICTOR
3	MyD88 cascade initiated on plasma membrane	0.188	MAP2K2/MAP2K3/MAP2K4/MAPK14/NFKBIB
3	Host Interactions of HIV factors	0.188	BTRC/DOCK2/KPNA1/NUP188/NUP35/NUP85/PSMC6/PSMD5
3	Signal Transduction	0.188	ADAM10/AKT3/ARHGAP15/ARHGAP26/ATP6V0D1/BTRC/CAMK4/CBL/DGKD/EIF4E/FRS2/ITCH/ITPR2/KRAS/MAP2K2/MAPK14/MDM2/MKNK1/MOB1B/NRG4/NUMB/PDE8A/PPP2R5E/PPP3CB/PSMC6/PSMD5/RICTOR/ROR1/SMARCA4/SOS1/ST3GAL3/STAT1/STK3/TFDP2/TNKS2/USP15/VAV3
3	TRAF6 mediated induction of NFkB and MAP kinases upon TLR7/8 or 9 activation	0.188	MAP2K2/MAP2K3/MAP2K4/MAPK14/NFKBIB
3	Stabilization of p53	0.188	ATM/MDM2/PSMC6/PSMD5
3	MyD88:Mal cascade initiated on plasma membrane	0.188	MAP2K2/MAP2K3/MAP2K4/MAPK14/NFKBIB
3	Toll Like Receptor TLR1:TLR2 Cascade	0.188	MAP2K2/MAP2K3/MAP2K4/MAPK14/NFKBIB
3	Toll Like Receptor 7/8 (TLR7/8) Cascade	0.188	MAP2K2/MAP2K3/MAP2K4/MAPK14/NFKBIB

3	Toll Like Receptor TLR6:TLR2 Cascade	0.188	MAP2K2/MAP2K3/MAP2K4/MAPK14/NFKBIB
3	Toll Like Receptor 2 (TLR2) Cascade	0.188	MAP2K2/MAP2K3/MAP2K4/MAPK14/NFKBIB
3	MyD88 dependent cascade initiated on endosome	0.188	MAP2K2/MAP2K3/MAP2K4/MAPK14/NFKBIB
3	MAP kinase activation in TLR cascade	0.191	MAP2K2/MAP2K3/MAP2K4/MAPK14
3	Adaptive Immune System	0.191	AKT3/ANAPC7/BTRC/CARD11/CBL/FBXW8/FRS2/ITCH/ITPR2/KRAS/LNPEP/MDM2/NFKBIB/NRG4/PPP2R5E/PSMC6/PSMD5/RICTOR/RNF4/SOS1/STIM1/UBE2G1/UBE2V2/UBE3C
3	Antigen processing	0.191	ANAPC7/BTRC/FBXW8/ITCH/LNPEP/PSMC6/PSMD5/RNF4/UBE2G1/UBE2V2/UBE3C
3	Nuclear Envelope Breakdown	0.197	NEK7/NUP188/NUP35/NUP85
3	Toll Like Receptor 9 (TLR9) Cascade	0.197	MAP2K2/MAP2K3/MAP2K4/MAPK14/NFKBIB
4	Signalling by NGF	0.050	ABR/ADAM17/AGO2/AKAP13/ARHGEF1/ARHGEF3/BRAF/FOXO1/FYN/GAB1/GSK3B/IRS2/ITGB3BP/ITPR1/MAPK1/MAPK8/MEF2C/PHLPP1/PRKCE/RAF1/RAP1A/RAPGEF1/RPS6KA5/RTN4/TNRC6A/TRIO
4	NCAM signaling for neurite out-growth	0.050	CACNA1D/COL6A3/FYN/MAPK1/PTPRA/RAF1/RPS6KA5/SPTBN1
4	Signal Transduction	0.050	ABR/ADAM17/AGO2/AKAP13/APP/ARHGAP12/ARHGAP25/ARHGAP44/ARHGEF1/ARHGEF3/ATP6V1H/BRAF/CD55/CDC73/COL6A3/DAAM1/ERBB2IP/FAM13B/FER/FLT1/FOXO1/FYN/GAB1/GAB2/GNA13/GNG7/GPC5/GRAP/GRM7/GSK3B/HECW1/IRS2/ITGB3BP/ITPR1/LATS1/LEF1/MAML2/MAPK1/MAPK8/MEF2C/MYO9B/OPHN1/PHLPP1/PRKAG2/PRKCE/PSMD12/PSMD14/PSME4/RAF1/RALBP1/RAP1A/RAPGEF1/RASA1/RNF111/ROCK2/RPS6KA5/RTN4/RYK/SH3KBP1/STK4/TAB2/TBL1X/TCF7/TGFBR2/TLE4/TNRC6A/TP53/TRIO/USP9X/WWOX
4	Frs2-mediated activation	0.061	BRAF/MAPK1/RAF1/RAP1A/RAPGEF1
4	Developmental Biology	0.061	CACNA1D/CDK19/CLASP2/COL6A3/CTNNA1/DLG1/EZR/FOXO1/FYN/GSK3B/MAPK1/MED13L/MED14/MED31/MEF2C/NCOA3/PAK1/PTPRA/RAF1/RANBP9/ROCK2/RPS6KA5/SIAH1/SPTBN1/TCF12/TRIO

4	Fc epsilon receptor (FCERI) signaling	0.061	AGO2/FOXO1/FYN/GAB1/GAB2/GSK3B/IRS2/ITPR1/MAP3K1/MAPK1/MAPK8/PAK1/PHLPP1/PLCG2/RAF1/TAB2/TNRC6A
4	Signaling by FGFR in disease	0.061	AGO2/BRAF/CPSF6/FOXO1/FYN/GAB1/GAB2/GSK3B/IRS2/ITPR1/LRRFIP1/MAPK1/PHLPP1/PRKCE/RAF1/TNRC6A
4	Immune System	0.061	ACTR2/ADAM17/AGO2/AP1G1/APP/ARIH1/ATG7/BLMH/CD55/CD59/CD81/CDC16/CTSC/DCTN4/DHX36/EIF4A3/EIF4G3/FBXW11/FOXO1/FYN/GAB1/GAB2/GSK3B/IL1RAP/IL6R/IRS2/ITPR1/KPNA5/LRRFIP1/MAP3K1/MAPK1/MAPK8/MEF2C/MYO9B/NLRP1/NPEPPS/NUP133/NUP160/NUP214/NUP88/NUPL1/PAK1/PELI2/PHLPP1/PIAS1/PIK3AP1/PLCG2/POLR3E/PRKCE/PSMD12/PSMD14/PSME4/RAF1/RANBP2/RAP1A/RAPGEF1/RIPK1/RNF111/RPS6KA5/SEC24B/SH3KBP1/TAB2/TANK/TNRC6A/TPP2/TRAF3/TRIP12/UBE2E1/UBE2H/UBE2K/UBR2/VPRBP
4	Prolonged ERK activation events	0.061	BRAF/MAPK1/RAF1/RAP1A/RAPGEF1
4	Nephrin interactions	0.061	CASK/CD2AP/FYN/IQGAP1/SPTBN1
4	ISG15 antiviral mechanism	0.061	ARIH1/EIF4A3/EIF4G3/KPNA5/NUP133/NUP160/NUP214/NUP88/NUPL1/RANBP2/UBE2E1
4	Antiviral mechanism by IFN-stimulated genes	0.061	ARIH1/EIF4A3/EIF4G3/KPNA5/NUP133/NUP160/NUP214/NUP88/NUPL1/RANBP2/UBE2E1
4	Vpr-mediated nuclear import of PICs	0.082	NUP133/NUP160/NUP214/NUP88/NUPL1/PSIP1/RANBP2
4	Signaling by SCF-KIT	0.082	AGO2/FER/FOXO1/FYN/GAB1/GAB2/GRAP/GSK3B/IRS2/MAPK1/PHLPP1/RAF1/TNRC6A
4	Cell-Cell communication	0.082	CASK/CD2AP/CD47/CLDN11/CTNNA1/FYN/IQGAP1/MAPK8/PAK1/SKAP2/SPTBN1
4	Signaling by ERBB4	0.086	ADAM17/AGO2/FOXO1/FYN/GAB1/GSK3B/IRS2/MAPK1/PHLPP1/RAF1/TAB2/TNRC6A/WWOX
4	NGF signalling via TRKA from the plasma membrane	0.086	AGO2/BRAF/FOXO1/FYN/GAB1/GSK3B/IRS2/ITPR1/MAPK1/MEF2C/PHLPP1/PRKCE/RAF1/RAP1A/RAPGEF1/RPS6KA5/TNRC6A
4	Downstream signal transduction	0.091	AGO2/FOXO1/FYN/GAB1/GSK3B/IRS2/ITPR1/MAPK1/PHLPP1/PRKCE/RAF1/RAPGEF1/RASA1/TNRC6A
4	ARMS-mediated activation	0.091	BRAF/MAPK1/RAF1/RAP1A

4	Interactions of Vpr with host cellular proteins	0.091	NUP133/NUP160/NUP214/NUP88/NUPL1/PSIP1/RANBP2
4	Regulation of Glucokinase by Glucokinase Regulatory Protein	0.091	NUP133/NUP160/NUP214/NUP88/NUPL1/RANBP2
4	Signaling by PDGF	0.091	AGO2/COL6A3/FOXO1/FYN/GAB1/GSK3B/IRS2/ITPR1/MAPK1/PHLPP1/PRKCE/RAF1/RAPGEF1/RASA1/TNRC6A
4	Signaling by Rho GTPases	0.091	ABR/AKAP13/ARHGAP12/ARHGAP25/ARHGAP44/ARHGEF3/FAM13B/MYO9B/OPHN1/RALBP1/TRIO
4	Rho GTPase cycle	0.091	ABR/AKAP13/ARHGAP12/ARHGAP25/ARHGAP44/ARHGEF3/FAM13B/MYO9B/OPHN1/RALBP1/TRIO
4	Signaling by FGFR	0.100	AGO2/BRAF/FOXO1/FYN/GAB1/GSK3B/IRS2/ITPR1/MAPK1/PHLPP1/PRKCE/RAF1/TNRC6A
4	Cytokine Signaling in Immune system	0.100	ADAM17/ARIH1/EIF4A3/EIF4G3/FYN/GAB2/IL1RAP/IL6R/IRS2/KPNA5/MAPK1/NUP133/NUP160/NUP214/NUP88/NUPL1/PELI2/PIAS1/RAF1/RANBP2/RAPGEF1/TAB2/UBE2E1
4	FCERI mediated MAPK activation	0.103	MAP3K1/MAPK1/MAPK8/PAK1/PLCG2/RAF1
4	Innate Immune System	0.103	ACTR2/AGO2/APP/CD55/CD59/DHX36/FOXO1/FYN/GAB1/GAB2/GSK3B/IRS2/ITPR1/LRRFIP1/MAP3K1/MAPK1/MAPK8/MEF2C/MYO9B/NLRP1/PAK1/PELI2/PHLPP1/PLCG2/POLR3E/PRKCE/RAF1/RIPK1/RPS6KA5/TAB2/TANK/TNRC6A/TRAF3/UBE2K
4	Signaling by ERBB2	0.103	AGO2/ERBB2IP/FOXO1/FYN/GAB1/GSK3B/IRS2/ITPR1/MAPK1/PHLPP1/PRKCE/RAF1/TNRC6A
4	DAP12 signaling	0.103	AGO2/FOXO1/FYN/GAB1/GSK3B/IRS2/ITPR1/MAPK1/PHLPP1/PLCG2/PRKCE/RAF1/TNRC6A
4	Signaling by EGFR	0.103	ADAM17/AGO2/FOXO1/FYN/GAB1/GSK3B/IRS2/ITPR1/MAPK1/PHLPP1/PRKCE/RAF1/SH3KBP1/TNRC6A
4	Axon guidance	0.103	CACNA1D/CLASP2/COL6A3/DLG1/EZR/FYN/GSK3B/MAPK1/PAK1/PTPRA/RAF1/RANBP9/ROCK2/RPS6KA5/SIAH1/SPTBN1/TRIO
4	p75 NTR receptor-mediated signalling	0.103	ABR/ADAM17/AKAP13/ARHGEF1/ARHGEF3/ITGB3BP/MAPK8/RTN4/TRIO

4	Downstream signaling of activated FGFR	0.103	AGO2/FOXO1/FYN/GAB1/GSK3B/IRS2/ITPR1/MAPK1/PHLPP1/PRKCE/RAF1/TNRC6A
4	Signaling by EGFR in Cancer	0.103	ADAM17/AGO2/FOXO1/FYN/GAB1/GSK3B/IRS2/ITPR1/MAPK1/PHLPP1/PRKCE/RAF1/SH3KBP1/TNRC6A
4	Rev-mediated nuclear export of HIV RNA	0.103	NUP133/NUP160/NUP214/NUP88/NUPL1/RANBP2
4	Nuclear import of Rev protein	0.103	NUP133/NUP160/NUP214/NUP88/NUPL1/RANBP2
4	Nuclear Pore Complex (NPC) Disassembly	0.103	NUP133/NUP160/NUP214/NUP88/NUPL1/RANBP2
4	Glucose transport	0.103	NUP133/NUP160/NUP214/NUP88/NUPL1/RANBP2
4	G alpha (12/13) signalling events	0.103	ABR/AKAP13/ARHGEF1/ARHGEF3/GNA13/GNG7/ROCK2/TRIO
4	DAP12 interactions	0.103	AGO2/FOXO1/FYN/GAB1/GSK3B/IRS2/ITPR1/MAPK1/PHLPP1/PLCG2/PRKCE/RAF1/TNRC6A
4	Hexose transport	0.113	NUP133/NUP160/NUP214/NUP88/NUPL1/RANBP2
4	Interactions of Rev with host cellular proteins	0.128	NUP133/NUP160/NUP214/NUP88/NUPL1/RANBP2
4	Signaling by the B Cell Receptor (BCR)	0.134	AGO2/FBXW11/FOXO1/FYN/GAB1/GSK3B/IRS2/ITPR1/PHLPP1/PIK3AP1/PLCG2/PSMD12/PSMD14/PSME4/SH3KBP1/TNRC6A
4	Role of LAT2/NTAL/LAB on calcium mobilization	0.137	AGO2/FOXO1/FYN/GAB1/GAB2/GSK3B/IRS2/PHLPP1/TNRC6A
4	Netrin-1 signaling	0.137	EZR/FYN/SIAH1/TRIO
4	Signalling to ERKs	0.152	BRAF/MAPK1/RAF1/RAP1A/RAPGEF1
4	PI3K events in ERBB4 signaling	0.152	AGO2/FOXO1/FYN/GAB1/GSK3B/IRS2/PHLPP1/TNRC6A
4	PIP3 activates AKT signaling	0.152	AGO2/FOXO1/FYN/GAB1/GSK3B/IRS2/PHLPP1/TNRC6A
4	PI-3K cascade	0.152	AGO2/FOXO1/FYN/GAB1/GSK3B/IRS2/PHLPP1/TNRC6A
4	PI3K events in ERBB2 signaling	0.152	AGO2/FOXO1/FYN/GAB1/GSK3B/IRS2/PHLPP1/TNRC6A
4	PI3K/AKT Signaling in Cancer	0.152	AGO2/FOXO1/FYN/GAB1/GSK3B/IRS2/PHLPP1/TNRC6A
4	GAB1 signalosome	0.158	AGO2/FOXO1/FYN/GAB1/GSK3B/IRS2/PHLPP1/TNRC6A
4	PI3K/AKT activation	0.158	AGO2/FOXO1/FYN/GAB1/GSK3B/IRS2/PHLPP1/TNRC6A
4	Host Interactions of HIV factors	0.158	AP1G1/ATP6V1H/FYN/NUP133/NUP160/NUP214/NUP88/NUPL1/PSIP1/PSMD12/PSMD14/PSME4/RANBP2
4	Antigen activates B Cell Receptor (BCR) leading to generation of second messengers	0.158	FYN/ITPR1/PIK3AP1/PLCG2/SH3KBP1

4	Ca2+ pathway	0.161	AGO2/GNG7/ITPR1/LEF1/TCF7/TNRC6A
4	NRAGE signals death through JNK	0.173	ABR/AKAP13/ARHGEF3/MAPK8/TRIO
4	Cell death signalling via NRAGE, NRIF and NADE	0.173	ABR/AKAP13/ARHGEF3/ITGB3BP/MAPK8/TRIO
4	Fcgamma receptor (FCGR) dependent phagocytosis	0.173	ACTR2/FYN/ITPR1/MAPK1/MYO9B/PAK1/PLCG2/PRKCE
4	Glucagon-like Peptide-1 (GLP1) regulates insulin secretion	0.173	GNG7/IQGAP1/ITPR1/RAP1A
4	Ion transport by P-type ATPases	0.173	ATP11B/ATP1B3/ATP2A3/ATP2B1
4	Cell Cycle	0.176	AHCTF1/ARPP19/ATR/CDC16/CDC25B/CEP192/CLASP2/DIDO1/DYRK1A/FBXW11/HUS1/ITGB3BP/LIN52/MAD1L1/MAPK1/MAPRE1/MLH3/NIPBL/NUP133/NUP160/NUP214/NUP88/NUPL1/OFD1/PAFAH1B1/PCM1/PRIM2/PSMD12/PSMD14/PSME4/RANBP2/RB1/RBBP8/RFWD2/RSF1/SMARCA5/STAG1/TP53/UBE2E1
4	Separation of Sister Chromatids	0.191	AHCTF1/CDC16/CLASP2/ITGB3BP/MAD1L1/MAPRE1/NUP133/NUP160/PAFAH1B1/PSMD12/PSMD14/PSME4/RANBP2/STAG1/UBE2E1
4	Activated TLR4 signalling	0.191	MAP3K1/MAPK1/MAPK8/MEF2C/PELI2/RIPK1/RPS6KA5/TAB2/TRAF3
5	NOTCH1 Intracellular Domain Regulates Transcription	0.161	CDK8/HDAC8/KAT2B/NCOR2
5	Constitutive Signaling by NOTCH1 PEST Domain Mutants	0.161	CDK8/HDAC8/KAT2B/NCOR2
5	Constitutive Signaling by NOTCH1 HD+PEST Domain Mutants	0.161	CDK8/HDAC8/KAT2B/NCOR2
5	PI Metabolism	0.161	FIG4/MTM1/MTMR6/PIP5K1B
5	Resolution of Sister Chromatid Cohesion	0.161	CLASP1/HDAC8/NSL1/PDS5B/SEC13/WAPAL
5	Phospholipid metabolism	0.161	AGPAT5/FIG4/MTM1/MTMR6/PIP5K1B/SLC44A2
5	Regulation of Apoptosis	0.161	APPL1/DCC/PAK2/PSMB7
5	Signaling by NOTCH1	0.161	CDK8/HDAC8/KAT2B/NCOR2
5	Signaling by NOTCH1 PEST Domain Mutants in Cancer	0.161	CDK8/HDAC8/KAT2B/NCOR2
5	Signaling by NOTCH1 in Cancer	0.161	CDK8/HDAC8/KAT2B/NCOR2
5	FBXW7 Mutants and NOTCH1 in Cancer	0.161	CDK8/HDAC8/KAT2B/NCOR2
5	Signaling by NOTCH1 t(7;9)(NOTCH1:M1580_K2555) Translocation Mutant	0.161	CDK8/HDAC8/KAT2B/NCOR2
5	Signaling by NOTCH1 HD Domain Mutants in Cancer	0.161	CDK8/HDAC8/KAT2B/NCOR2

5	Signaling by NOTCH1 HD+PEST Domain Mutants in Cancer	0.161	CDK8/HDAC8/KAT2B/NCOR2
5	Mitotic Prometaphase	0.161	CLASP1/HDAC8/NSL1/PDS5B/SEC13/WAPAL
5	RNA Polymerase I, RNA Polymerase III, and Mitochondrial Transcription	0.161	CDK7/KAT2B/SNAPC5/TAF1B
5	G2/M Transition	0.161	CDK11B/CDK7/CLASP1/CSNK1E/DYNC1I2/WEE1
5	Mitotic G2-G2/M phases	0.161	CDK11B/CDK7/CLASP1/CSNK1E/DYNC1I2/WEE1
5	Signaling by TGF-beta Receptor Complex	0.161	CDK8/NCOR2/TGFB1/UCHL5
5	Loss of Function of SMAD4 in Cancer	0.161	CDK8/NCOR2/TGFB1/UCHL5
5	Loss of Function of SMAD2/3 in Cancer	0.161	CDK8/NCOR2/TGFB1/UCHL5
5	Signaling by TGF-beta Receptor Complex in Cancer	0.161	CDK8/NCOR2/TGFB1/UCHL5
5	SMAD2/3 Phosphorylation Motif Mutants in Cancer	0.161	CDK8/NCOR2/TGFB1/UCHL5
5	SMAD4 MH2 Domain Mutants in Cancer	0.161	CDK8/NCOR2/TGFB1/UCHL5
5	SMAD2/3 MH2 Domain Mutants in Cancer	0.161	CDK8/NCOR2/TGFB1/UCHL5
5	Loss of Function of TGFBR2 in Cancer	0.161	CDK8/NCOR2/TGFB1/UCHL5
5	TGFBR2 MSI Frameshift Mutants in Cancer	0.161	CDK8/NCOR2/TGFB1/UCHL5
5	TGFBR2 Kinase Domain Mutants in Cancer	0.161	CDK8/NCOR2/TGFB1/UCHL5
5	TGFBR1 KD Mutants in Cancer	0.161	CDK8/NCOR2/TGFB1/UCHL5
5	Loss of Function of TGFBR1 in Cancer	0.161	CDK8/NCOR2/TGFB1/UCHL5
5	TGFBR1 LBD Mutants in Cancer	0.161	CDK8/NCOR2/TGFB1/UCHL5
5	Asparagine N-linked glycosylation	0.175	GLB1/MAN1A1/PMM2/SEC13/ST6GAL1
5	Separation of Sister Chromatids	0.187	CLASP1/HDAC8/NSL1/PDS5B/PSMB7/SEC13/WAPAL
7	PCP/CE pathway	0.000	PFN1/PSMB8/PSMB9/PSMC4/RAC2/RAC3
7	beta-catenin independent WNT signaling	0.001	PFN1/PSMB8/PSMB9/PSMC4/RAC2/RAC3
7	Vpu mediated degradation of CD4	0.001	PSMB8/PSMB9/PSMC4/SKP1
7	SCF(Skp2)-mediated degradation of p27/p21	0.001	PSMB8/PSMB9/PSMC4/SKP1
7	SCF-beta-TrCP mediated degradation of Emi1	0.001	PSMB8/PSMB9/PSMC4/SKP1
7	Vif-mediated degradation of APOBEC3G	0.001	APOBEC3G/PSMB8/PSMB9/PSMC4
7	ER-Phagosome pathway	0.001	HLA-G/PSMB8/PSMB9/PSMC4
7	Cyclin E associated events during G1/S transition	0.001	PSMB8/PSMB9/PSMC4/SKP1
7	Cyclin A:Cdk2-associated events at S phase entry	0.001	PSMB8/PSMB9/PSMC4/SKP1
7	Antigen processing-Cross presentation	0.001	HLA-G/PSMB8/PSMB9/PSMC4

7	Activation of NF-kappaB in B cells	0.001	PSMB8/PSMB9/PSMC4/SKP1
7	Regulation of APC/C activators between G1/S and early anaphase	0.002	PSMB8/PSMB9/PSMC4/SKP1
7	APC/C-mediated degradation of cell cycle proteins	0.002	PSMB8/PSMB9/PSMC4/SKP1
7	Regulation of mitotic cell cycle	0.002	PSMB8/PSMB9/PSMC4/SKP1
7	Host Interactions of HIV factors	0.002	APOBEC3G/PSMB8/PSMB9/PSMC4/SKP1
7	Interferon Signaling	0.002	HLA-DRB5/HLA-G/ISG20/PSMB8/UBE2L6
7	Platelet activation, signaling and aggregation	0.003	CAP1/CDC42/PFN1/POTEM/RAC2
7	Adaptive Immune System	0.003	CD79B/CDC42/CTLA4/HLA-DRB5/HLA-G/PSMB8/PSMB9/PSMC4/SKP1/UBE2L6
7	G1/S Transition	0.003	PSMB8/PSMB9/PSMC4/SKP1
7	Signaling by Wnt	0.003	PFN1/PSMB8/PSMB9/PSMC4/RAC2/RAC3
7	Signaling by the B Cell Receptor (BCR)	0.004	CD79B/PSMB8/PSMB9/PSMC4/SKP1
7	Class I MHC mediated antigen processing & presentation	0.004	HLA-G/PSMB8/PSMB9/PSMC4/SKP1/UBE2L6
7	Cytokine Signaling in Immune system	0.004	HLA-DRB5/HLA-G/ISG20/PSMB8/SKP1/UBE2L6
7	Mitotic G1-G1/S phases	0.005	PSMB8/PSMB9/PSMC4/SKP1
7	S Phase	0.006	PSMB8/PSMB9/PSMC4/SKP1
7	Antigen processing	0.007	PSMB8/PSMB9/PSMC4/SKP1/UBE2L6
7	Downstream signaling events of B Cell Receptor (BCR)	0.008	PSMB8/PSMB9/PSMC4/SKP1
7	HIV Infection	0.010	APOBEC3G/PSMB8/PSMB9/PSMC4/SKP1
7	Immune System	0.013	BIRC3/CD79B/CDC42/CTLA4/HLA-DRB5/HLA-G/ISG20/PSMB8/PSMB9/PSMC4/SKP1/UBE2L6
7	Axon guidance	0.013	CAP1/CDC42/PFN1/RAC2
7	Signal Transduction	0.015	CDC42/FCER2/PFN1/PPP1CA/PSMB8/PSMB9/PSMC4/RAC2/RAC3/RPTOR/SKP1
7	Metabolism of mRNA	0.018	PSMB8/PSMB9/PSMC4/RPS3A
7	Metabolism of RNA	0.025	PSMB8/PSMB9/PSMC4/RPS3A
7	Disease	0.025	APOBEC3G/CDC42/CYB5R3/MDH2/PPP1CA/PSMB8/PSMB9/PSMC4/RPS3A/SKP1
7	Hemostasis	0.028	CAP1/CDC42/PFN1/POTEM/RAC2
7	Developmental Biology	0.034	CAP1/CDC42/PFN1/RAC2

8	Transport of Mature mRNA derived from an Intron-Containing Transcript	0.002	RBM8A/SRRM1/SRSF3/SRSF7/SRSF9/UPF3B
8	Transport of Mature Transcript to Cytoplasm	0.002	RBM8A/SRRM1/SRSF3/SRSF7/SRSF9/UPF3B
8	Cleavage of Growing Transcript in the Termination Region	0.002	RBM8A/SNRPG/SRRM1/SRSF3/SRSF7/SRSF9/UPF3B
8	RNA Polymerase II Transcription Termination	0.002	RBM8A/SNRPG/SRRM1/SRSF3/SRSF7/SRSF9/UPF3B
8	Post-Elongation Processing of the Transcript	0.002	RBM8A/SNRPG/SRRM1/SRSF3/SRSF7/SRSF9/UPF3B
8	Metabolism of mRNA	0.002	EIF4A2/KHSRP/LSM5/LSM6/PAIP1/PSMA7/PSMB4/PSMD7/RBM8A/RPL22/RPL28/RPS17/RPS2/UPF3B
8	Metabolism of RNA	0.002	EIF4A2/KHSRP/LSM5/LSM6/PAIP1/PSMA7/PSMB4/PSMD7/RBM8A/RPL22/RPL28/RPS17/RPS2/SNRPG/UPF3B
8	mRNA Splicing - Major Pathway	0.002	GTF2F1/PCBP1/POLR2K/RBM8A/SNRPG/SRRM1/SRSF3/SRSF7/SRSF9/TXNL4A/UPF3B
8	mRNA Splicing	0.002	GTF2F1/PCBP1/POLR2K/RBM8A/SNRPG/SRRM1/SRSF3/SRSF7/SRSF9/TXNL4A/UPF3B
8	Post-Elongation Processing of Intron-Containing pre-mRNA	0.002	RBM8A/SRRM1/SRSF3/SRSF7/SRSF9/UPF3B
8	mRNA 3'-end processing	0.002	RBM8A/SRRM1/SRSF3/SRSF7/SRSF9/UPF3B
8	Processing of Capped Intron-Containing Pre-mRNA	0.002	GTF2F1/PCBP1/POLR2K/RBM8A/SNRPG/SRRM1/SRSF3/SRSF7/SRSF9/TXNL4A/UPF3B
8	Transcription	0.003	CCNH/GTF2F1/POLR2K/RBM8A/SNRPG/SRRM1/SRSF3/SRSF7/SRSF9/TAF1D/TFAM/UPF3B
8	RNA Polymerase II Transcription	0.003	CCNH/GTF2F1/POLR2K/RBM8A/SNRPG/SRRM1/SRSF3/SRSF7/SRSF9/UPF3B
8	Gene Expression	0.008	AIMP1/CCNH/DARS/EIF1AX/EIF4A2/EPRS/GTF2F1/HIST1H2BK/KHSRP/LSM5/LSM6/MAML1/MED30/NR4A2/PAIP1/PCBP1/POLR2K/PSMA7/PSMB4/PSMD7/RBM8A/RPL22/RPL28/RPS17/RPS2/SEC61B/SNRPG/SRP54/SRRM1/SRSF3/SRSF7/SRSF9/TAF1D/TFAM/TGIF1/TXNL4A/UPF3B
8	Cyclin E associated events during G1/S transition	0.014	CCNH/CDKN1A/CDKN1B/PSMA7/PSMB4/PSMD7
8	Cyclin A:Cdk2-associated events at S phase entry	0.016	CCNH/CDKN1A/CDKN1B/PSMA7/PSMB4/PSMD7
8	mRNA Splicing - Minor Pathway	0.027	GTF2F1/POLR2K/SNRPG/SRSF7/TXNL4A
8	Cyclin D associated events in G1	0.027	CCND2/CCNH/CDKN1A/CDKN1B
8	G1 Phase	0.027	CCND2/CCNH/CDKN1A/CDKN1B

8	Translation	0.027	EIF1AX/EIF4A2/RPL22/RPL28/RPS17/RPS2/SEC61B/SRP54
8	SCF(Skp2)-mediated degradation of p27/p21	0.027	CDKN1A/CDKN1B/PSMA7/PSMB4/PSMD7
8	L13a-mediated translational silencing of Ceruloplasmin expression	0.027	EIF1AX/EIF4A2/RPL22/RPL28/RPS17/RPS2
8	3' -UTR-mediated translational regulation	0.027	EIF1AX/EIF4A2/RPL22/RPL28/RPS17/RPS2
8	Nonsense-Mediated Decay (NMD)	0.027	RBM8A/RPL22/RPL28/RPS17/RPS2/UPF3B
8	Nonsense Mediated Decay (NMD) enhanced by the Exon Junction Complex (EJC)	0.027	RBM8A/RPL22/RPL28/RPS17/RPS2/UPF3B
8	Mitotic G1-G1/S phases	0.028	CCND2/CCNH/CDKN1A/CDKN1B/DBF4/PSMA7/PSMB4/PSMD7
8	G1/S Transition	0.028	CCNH/CDKN1A/CDKN1B/DBF4/PSMA7/PSMB4/PSMD7
8	GTP hydrolysis and joining of the 60S ribosomal subunit	0.028	EIF1AX/EIF4A2/RPL22/RPL28/RPS17/RPS2
8	p53-Dependent G1 DNA Damage Response	0.028	CDKN1A/CDKN1B/PSMA7/PSMB4/PSMD7
8	p53-Dependent G1/S DNA damage checkpoint	0.028	CDKN1A/CDKN1B/PSMA7/PSMB4/PSMD7
8	SRP-dependent cotranslational protein targeting to membrane	0.028	RPL22/RPL28/RPS17/RPS2/SEC61B/SRP54
8	G1/S DNA Damage Checkpoints	0.032	CDKN1A/CDKN1B/PSMA7/PSMB4/PSMD7
8	Eukaryotic Translation Initiation	0.037	EIF1AX/EIF4A2/RPL22/RPL28/RPS17/RPS2
8	Cap-dependent Translation Initiation	0.037	EIF1AX/EIF4A2/RPL22/RPL28/RPS17/RPS2
8	Formation of a pool of free 40S subunits	0.042	EIF1AX/RPL22/RPL28/RPS17/RPS2
8	Translation initiation complex formation	0.051	EIF1AX/EIF4A2/RPS17/RPS2
8	Orc1 removal from chromatin	0.051	CDKN1A/CDKN1B/PSMA7/PSMB4/PSMD7
8	Switching of origins to a post-replicative state	0.051	CDKN1A/CDKN1B/PSMA7/PSMB4/PSMD7
8	Activation of the mRNA upon binding of the cap-binding complex and eIFs, and subsequent binding to 43S	0.051	EIF1AX/EIF4A2/RPS17/RPS2
8	Ribosomal scanning and start codon recognition	0.051	EIF1AX/EIF4A2/RPS17/RPS2
8	Removal of licensing factors from origins	0.051	CDKN1A/CDKN1B/PSMA7/PSMB4/PSMD7
8	Regulation of DNA replication	0.051	CDKN1A/CDKN1B/PSMA7/PSMB4/PSMD7
8	Metabolism of amino acids and derivatives	0.054	DLD/GLUD1/HSD17B10/ODC1/PSMA7/PSMB4/PSMD7/SAT1
8	Influenza Viral RNA Transcription and Replication	0.054	RPL22/RPL28/RPS17/RPS2
8	Viral mRNA Translation	0.054	RPL22/RPL28/RPS17/RPS2
8	Peptide chain elongation	0.056	RPL22/RPL28/RPS17/RPS2

8	Eukaryotic Translation Termination	0.056	RPL22/RPL28/RPS17/RPS2
8	Regulation of ornithine decarboxylase (ODC)	0.059	ODC1/PSMA7/PSMB4/PSMD7
8	Deadenylation-dependent mRNA decay	0.059	EIF4A2/LSM5/LSM6/PAIP1
8	DNA Replication	0.060	CDKN1A/CDKN1B/DBF4/PSMA7/PSMB4/PSMD7
8	Eukaryotic Translation Elongation	0.061	RPL22/RPL28/RPS17/RPS2
8	Disease	0.062	AP2B1/APH1B/ATP6V1D/CCNH/CDKN1A/CDKN1B/CSK/ERBB4/GNGT1/GTF2F1/HIST1H2BK/HSP90AA1/ITM2B/MAML1/METAP2/PAPSS1/POLR2K/PRKAR1A/PSMA7/PSMB4/PSMD7/RAE1/RPL22/RPL28/RPS17/RPS2/STRAP/TGIF1
8	Influenza Life Cycle	0.062	RPL22/RPL28/RPS17/RPS2
8	Nonsense Mediated Decay (NMD) independent of the Exon Junction Complex (EJC)	0.062	RPL22/RPL28/RPS17/RPS2
8	Signaling by EGFR in Cancer	0.063	AP2B1/CDKN1A/CDKN1B/CSK/ERBB4/HSP90AA1/PRKAR1A
8	ER-Phagosome pathway	0.065	PSMA7/PSMB4/PSMD7/SEC61B
8	Cellular responses to stress	0.070	CBX6/CDKN1A/CDKN1B/CUL2/CYCS/FOS/HIST1H2BK/HSP90AA1/JUN/KDM6B
8	Downstream signaling events of B Cell Receptor (BCR)	0.074	CDKN1A/CDKN1B/ERBB4/NFKBIA/PSMA7/PSMB4/PSMD7
8	Influenza Infection	0.074	RPL22/RPL28/RPS17/RPS2
8	RNA Polymerase I, RNA Polymerase III, and Mitochondrial Transcription	0.074	CCNH/POLR2K/TAF1D/TFAM
8	Immune System	0.075	AP2B1/CASP4/CDKN1A/CDKN1B/CSK/CUL2/EGR1/EIF4A2/ERBB4/FOS/HLA-DQA2/HSP90AA1/IFI16/IFNGR1/IL6ST/JUN/KIF5B/NFKBIA/POLR2K/PRKAR1A/PSMA7/PSMB4/PSMD7/RAE1/RNF144B/RNF6/SEC61B/TNFRSF14/TUBA4A/UBE2J1
8	Antigen processing-Cross presentation	0.091	PSMA7/PSMB4/PSMD7/SEC61B
8	Metabolism of proteins	0.091	ARSI/ATF3/ATF4/DNAJC19/EIF1AX/EIF4A2/KIF5B/NOP56/PIGF/RPL22/RPL28/RPS17/RPS2/SEC61B/SERP1/SRP54/TUBA4A
8	Oxidative Stress Induced Senescence	0.091	CBX6/FOS/HIST1H2BK/JUN/KDM6B
8	L1CAM interactions	0.092	AP2B1/RDX/SDCBP/TUBA4A
8	Cell Cycle Checkpoints	0.092	CDKN1A/CDKN1B/DBF4/PSMA7/PSMB4/PSMD7

8	Activation of NF-kappaB in B cells	0.095	NFKBIA/PSMA7/PSMB4/PSMD7
8	Cellular Senescence	0.095	CBX6/CDKN1A/CDKN1B/FOS/HIST1H2BK/JUN/KDM6B
8	S Phase	0.095	CCNH/CDKN1A/CDKN1B/PSMA7/PSMB4/PSMD7
8	Respiratory electron transport	0.097	COX7C/CYCS/NDUFA6/NDUFS5
8	Adaptive Immune System	0.097	AP2B1/CDKN1A/CDKN1B/CSK/CUL2/ERBB4/HLA-DQA2/KIF5B/NFKBIA/PSMA7/PSMB4/PSMD7/RNF144B/RNF6/SEC61B/TNFRSF14/TUBA4A/UBE2J1
8	GAB1 signalosome	0.099	CDKN1A/CDKN1B/CSK/ERBB4
8	Synthesis of DNA	0.100	CDKN1A/CDKN1B/PSMA7/PSMB4/PSMD7
8	NoRC negatively regulates rRNA expression	0.106	CCNH/HIST1H2BK/POLR2K/TAF1D
8	Signaling by EGFR	0.106	AP2B1/CDKN1A/CDKN1B/CSK/ERBB4/PRKAR1A
8	PCP/CE pathway	0.113	AP2B1/PSMA7/PSMB4/PSMD7
8	Negative epigenetic regulation of rRNA expression	0.113	CCNH/HIST1H2BK/POLR2K/TAF1D
8	Signaling by the B Cell Receptor (BCR)	0.116	CDKN1A/CDKN1B/ERBB4/NFKBIA/PSMA7/PSMB4/PSMD7
8	M/G1 Transition	0.125	DBF4/PSMA7/PSMB4/PSMD7
8	DNA Replication Pre-Initiation	0.125	DBF4/PSMA7/PSMB4/PSMD7
8	GPCR ligand binding	0.129	CXCR5/GNGT1/PTGER4/PYY
8	Fc epsilon receptor (FCERI) signaling	0.131	CDKN1A/CDKN1B/ERBB4/FOS/JUN/NFKBIA
8	Regulation of mRNA stability by proteins that bind AU-rich elements	0.148	KHSRP/PSMA7/PSMB4/PSMD7
8	Signaling by ERBB2	0.150	CDKN1A/CDKN1B/ERBB4/HSP90AA1/PRKAR1A
8	beta-catenin independent WNT signaling	0.150	AP2B1/GNGT1/PSMA7/PSMB4/PSMD7
8	Respiratory electron transport, ATP synthesis by chemiosmotic coupling, and heat production by uncoupling proteins.	0.154	COX7C/CYCS/NDUFA6/NDUFS5
8	HIV Infection	0.178	AP2B1/CCNH/GTF2F1/POLR2K/PSMA7/PSMB4/PSMD7/RAE1
8	Epigenetic regulation of gene expression	0.179	CCNH/HIST1H2BK/POLR2K/TAF1D
8	MHC class II antigen presentation	0.200	AP2B1/HLA-DQA2/KIF5B/TUBA4A
8	The citric acid (TCA) cycle and respiratory electron transport	0.200	COX7C/CYCS/DLD/NDUFA6/NDUFS5
9	Immune System	0.063	CDH1/DDOST/EIF4G2/HLA-DOB/PDIA3/PSME2/PTEN
9	Adaptive Immune System	0.063	CDH1/HLA-DOB/PDIA3/PSME2/PTEN
9	Gene Expression	0.137	DDOST/HDAC1/PSME2/SNRPB/SRPRB/ZNF490

10	Influenza Viral RNA Transcription and Replication	0.000	RPL24/RPL27/RPL30/RPL35/RPL37/RPL37A/RPL38/RPL39/RPL6/RPS15/RPS15A/RPS21/RPS26
10	Viral mRNA Translation	0.000	RPL24/RPL27/RPL30/RPL35/RPL37/RPL37A/RPL38/RPL39/RPL6/RPS15/RPS15A/RPS21/RPS26
10	Peptide chain elongation	0.000	RPL24/RPL27/RPL30/RPL35/RPL37/RPL37A/RPL38/RPL39/RPL6/RPS15/RPS15A/RPS21/RPS26
10	Eukaryotic Translation Termination	0.000	RPL24/RPL27/RPL30/RPL35/RPL37/RPL37A/RPL38/RPL39/RPL6/RPS15/RPS15A/RPS21/RPS26
10	Eukaryotic Translation Elongation	0.000	RPL24/RPL27/RPL30/RPL35/RPL37/RPL37A/RPL38/RPL39/RPL6/RPS15/RPS15A/RPS21/RPS26
10	Influenza Life Cycle	0.000	RPL24/RPL27/RPL30/RPL35/RPL37/RPL37A/RPL38/RPL39/RPL6/RPS15/RPS15A/RPS21/RPS26
10	Nonsense Mediated Decay (NMD) independent of the Exon Junction Complex (EJC)	0.000	RPL24/RPL27/RPL30/RPL35/RPL37/RPL37A/RPL38/RPL39/RPL6/RPS15/RPS15A/RPS21/RPS26
10	Influenza Infection	0.000	RPL24/RPL27/RPL30/RPL35/RPL37/RPL37A/RPL38/RPL39/RPL6/RPS15/RPS15A/RPS21/RPS26
10	L13a-mediated translational silencing of Ceruloplasmin expression	0.000	EIF2S3/RPL24/RPL27/RPL30/RPL35/RPL37/RPL37A/RPL38/RPL39/RPL6/RPS15/RPS15A/RPS21/RPS26
10	3' -UTR-mediated translational regulation	0.000	EIF2S3/RPL24/RPL27/RPL30/RPL35/RPL37/RPL37A/RPL38/RPL39/RPL6/RPS15/RPS15A/RPS21/RPS26
10	GTP hydrolysis and joining of the 60S ribosomal subunit	0.000	EIF2S3/RPL24/RPL27/RPL30/RPL35/RPL37/RPL37A/RPL38/RPL39/RPL6/RPS15/RPS15A/RPS21/RPS26
10	Formation of a pool of free 40S subunits	0.000	RPL24/RPL27/RPL30/RPL35/RPL37/RPL37A/RPL38/RPL39/RPL6/RPS15/RPS15A/RPS21/RPS26
10	Eukaryotic Translation Initiation	0.000	EIF2S3/RPL24/RPL27/RPL30/RPL35/RPL37/RPL37A/RPL38/RPL39/RPL6/RPS15/RPS15A/RPS21/RPS26
10	Cap-dependent Translation Initiation	0.000	EIF2S3/RPL24/RPL27/RPL30/RPL35/RPL37/RPL37A/RPL38/RPL39/RPL6/RPS15/RPS15A/RPS21/RPS26
10	Nonsense-Mediated Decay (NMD)	0.000	RPL24/RPL27/RPL30/RPL35/RPL37/RPL37A/RPL38/RPL39/RPL6/RPS15/RPS15A/RPS21/RPS26
10	Nonsense Mediated Decay (NMD) enhanced by the Exon Junction Complex (EJC)	0.000	RPL24/RPL27/RPL30/RPL35/RPL37/RPL37A/RPL38/RPL39/RPL6/RPS15/RPS15A/RPS21/RPS26

10	SRP-dependent cotranslational protein targeting to membrane	0.000	RPL24/RPL27/RPL30/RPL35/RPL37/RPL37A/RPL38/RPL39/RPL6/RPS15/RPS15A/RPS21/RPS26
10	Translation	0.000	EIF2S3/RPL24/RPL27/RPL30/RPL35/RPL37/RPL37A/RPL38/RPL39/RPL6/RPS15/RPS15A/RPS21/RPS26
10	Metabolism of proteins	0.000	COX17/CPN1/DDIT3/DNAJB9/EIF2S3/EIF5A/HERPUD1/MGAT1/RPL24/RPL27/RPL30/RPL35/RPL37/RPL37A/RPL38/RPL39/RPL6/RPS15/RPS15A/RPS21/RPS26/SAMM50/TOMM7/TUBA1A
10	Metabolism of RNA	0.000	HSPA8/RPL24/RPL27/RPL30/RPL35/RPL37/RPL37A/RPL38/RPL39/RPL6/RPS15/RPS15A/RPS21/RPS26/SNRPF/ZFP36
10	Metabolism of mRNA	0.000	HSPA8/RPL24/RPL27/RPL30/RPL35/RPL37/RPL37A/RPL38/RPL39/RPL6/RPS15/RPS15A/RPS21/RPS26/ZFP36
10	Formation of the ternary complex, and subsequently, the 43S complex	0.000	EIF2S3/RPS15/RPS15A/RPS21/RPS26
10	Translation initiation complex formation	0.000	EIF2S3/RPS15/RPS15A/RPS21/RPS26
10	Activation of the mRNA upon binding of the cap-binding complex and eIFs, and subsequent binding to 43S	0.000	EIF2S3/RPS15/RPS15A/RPS21/RPS26
10	Ribosomal scanning and start codon recognition	0.000	EIF2S3/RPS15/RPS15A/RPS21/RPS26
10	Gene Expression	0.003	EIF2S3/HNRNPF/HSPA8/KARS/PCBP2/PHF5A/POLR1D/PTBP1/RPL24/RPL27/RPL30/RPL35/RPL37/RPL37A/RPL38/RPL39/RPL6/RPS15/RPS15A/RPS21/RPS26/SNRPB2/SNRPF/TAF9/ZFP36
10	Disease	0.005	CHMP2A/CHMP2B/ENO1/PFKFB2/PPP1R15A/RPL24/RPL27/RPL30/RPL35/RPL37/RPL37A/RPL38/RPL39/RPL6/RPS15/RPS15A/RPS21/RPS26/SLC25A5/SNCA/TAF9/TLE1
10	mRNA Splicing - Major Pathway	0.008	HNRNPF/PCBP2/PHF5A/PTBP1/SNRPB2/SNRPF
10	mRNA Splicing	0.008	HNRNPF/PCBP2/PHF5A/PTBP1/SNRPB2/SNRPF
10	Processing of Capped Intron-Containing Pre-mRNA	0.009	HNRNPF/PCBP2/PHF5A/PTBP1/SNRPB2/SNRPF
10	Respiratory electron transport, ATP synthesis by chemiosmotic coupling, and heat production by uncoupling proteins.	0.022	ATP5I/NDUFB10/NDUFB3/NDUFS6
10	Membrane Trafficking	0.050	CHMP2A/CHMP2B/HSPA8/TUBA1A/YWHAQ

10	The citric acid (TCA) cycle and respiratory electron transport	0.066	ATP5I/NDUFB10/NDUFB3/NDUFS6
10	Cellular responses to stress	0.116	CDC26/DNAJB1/HSPA8/RPA3/SOD1

Supplementary table 8 SV detected in the bulk WGS data

ChrA	PosA	OrtA	ChrB	PosB	OrtB	Type	GeneA	GeneB	Samples
chr1	101978981	-	chr1	65918241	+	ITX	S1PR1(dist=271905)	LEPR	Year_14,Year_28
chr1	106396413	-	chr1	97350030	+	ITX	LOC100129138(dist=1776720)	PTBP2(dist=69425)	Year_10,Year_14,Year_23,Year_24,Year_26
chr1	114646030	+	chr1	114654351	-	ITX	SYT6	SYT6	Year_10,Year_14,Year_21,Year_23,Year_24,Year_26,Year_28
chr1	145183392	-	chr1	120690692	+	ITX	SEC22B(dist=66470)	NOTCH2(dist=78375)	Year_10,Year_14,Year_21,Year_23,Year_24,Year_26,Year_28
chr1	148183891	-	chr1	145109010	+	ITX	NBPF14(dist=158043)	SEC22B	Year_23
chr1	16415316	+	chr1	16416097	-	INV	FAM131C(dist=15189)	FAM131C(dist=15970)	Year_21
chr1	16416094	+	chr1	16415316	-	ITX	FAM131C(dist=15189)	-	Year_10,Year_21,Year_23,Year_26,Year_28
chr1	168186186	-	chr1	182274316	+	ITX	TIPRL(dist=14835)	ZNF648(dist=243469)	Year_10
chr1	175200569	+	chr1	175201937	-	ITX	KIAA0040(dist=38340)	KIAA0040(dist=39708)	Year_10,Year_14,Year_23,Year_26,Year_28
chr1	178397600	-	chr1	73768790	+	ITX	RASAL2	NEGR1(dist=1020513)	Year_21
chr1	182274316	-	chr1	168186186	+	ITX	ZNF648(dist=243469)	TIPRL(dist=14835)	Year_21,Year_23
chr1	187464828	+	chr1	187466727	-	ITX	PLA2G4A(dist=506715)	PLA2G4A(dist=508614)	Year_10,Year_14,Year_21,Year_23,Year_24,Year_26,Year_28
chr1	187466730	-	chr1	187466476	+	ITX	PLA2G4A(dist=508617)	PLA2G4A(dist=508363)	Year_14,Year_21,Year_24,Year_28
chr1	194638694	-	chr1	194638619	+	ITX	CDC73(dist=1414752)	CDC73(dist=1414677)	Year_28

chr1	197756788	+	chr1	197757985	-	INV	DENND1B(dist=12165)	DENND1B(dist=13362)	Year_23,Year_26
chr1	197756788	+	chr1	197757986	-	ITX	DENND1B(dist=12165)	DENND1B(dist=13363)	Year_10,Year_21,Year_23,Year_24,Year_26,Year_28
chr1	197757985	-	chr1	197756789	+	ITX	DENND1B(dist=13362)	DENND1B(dist=12166)	Year_10,Year_14,Year_21,Year_23,Year_26,Year_28
chr1	197757986	-	chr1	197756789	+	INV	DENND1B(dist=13363)	DENND1B(dist=12166)	Year_10,Year_21,Year_23
chr1	207292362	+	chr1	207292429	-	ITX	C4BPA	C4BPA	Year_10,Year_14,Year_21,Year_23,Year_24,Year_26,Year_28
chr1	237566106	+	chr1	237566207	-	ITX	RYR2	RYR2	Year_10,Year_14,Year_21,Year_23,Year_24,Year_26,Year_28
chr1	237566205	-	chr1	237566104	+	ITX	RYR2	RYR2	Year_10,Year_14,Year_21,Year_23,Year_24,Year_26,Year_28
chr1	240116379	+	chr1	240116680	-	ITX	CHRM3(dist=43662)	CHRM3(dist=43963)	Year_10,Year_14,Year_21,Year_23,Year_28
chr1	30878513	-	chr1	30878819	+	ITX	PTPRU(dist=1225188)	PTPRU(dist=1225494)	Year_26
chr1	30878819	-	chr1	30878513	+	ITX	PTPRU(dist=1225494)	PTPRU(dist=1225188)	Year_10,Year_14,Year_21,Year_23,Year_24,Year_28
chr1	36733250	+	chr1	36734642	-	ITX	THRAP3	THRAP3	Year_10,Year_14,Year_21,Year_23,Year_24,Year_26,Year_28
chr1	44059296	+	chr1	44059886	-	ITX	PTPRF	PTPRF	Year_10,Year_14,Year_21,Year_23,Year_24,Year_26,Year_28
chr1	45417937	+	chr1	163314443	-	ITX	EIF2B3	NUF2	Year_14,Year_23,Year_26,Year_28

chr1	80795112	-	chr1	80794624	+	ITX	ELTD1(dist=1322617)	ELTD1(dist=1322129)	Year_10,Year_14,Year_23,Year_24,Year_26
chr1	81660351	+	chr1	81661558	-	ITX	NONE(dist=NONE)	NONE(dist=NONE)	Year_10,Year_14,Year_21,Year_23,Year_24,Year_26,Year_28
chr1	81661371	-	chr1	81660357	+	ITX	NONE(dist=NONE)	NONE(dist=NONE)	Year_10,Year_14,Year_21,Year_23,Year_24,Year_26,Year_28
chr10	107143136	+	chr10	9525646	-	ITX	SORCS3(dist=118143)	GATA3(dist=1408482)	Year_10
chr10	127190418	-	chr10	127201102	+	ITX	CTBP2(dist=340794)	CTBP2(dist=351478)	Year_10,Year_23,Year_28
chr10	127190681	+	chr10	127197222	-	ITX	CTBP2(dist=341057)	CTBP2(dist=347598)	Year_10,Year_23,Year_28
chr10	27605765	+	chr10	27622858	-	ITX	LOC387646(dist=64530)	LOC387646(dist=81623)	Year_24,Year_26
chr10	38772446	+	chr10	42813717	-	INV	LOC399744(dist=31365)	NONE(dist=NONE)	Year_24
chr10	38777672	+	chr10	42797005	-	ITX	LOC399744(dist=36591)	NONE(dist=NONE)	Year_14
chr10	38783805	+	chr10	42370086	-	ITX	LOC399744(dist=42724)	NONE(dist=NONE)	Year_14
chr10	38803534	+	chr10	39110664	-	ITX	LOC399744(dist=62453)	ACTR3BP5(dist=119293)	Year_28
chr10	38873809	+	chr10	42812089	-	ITX	LOC399744(dist=132728)	NONE(dist=NONE)	Year_21
chr10	39103490	+	chr10	38782077	-	ITX	ACTR3BP5(dist=112119)	LOC399744(dist=40996)	Year_21
chr10	39117845	-	chr10	39081984	+	INV	ACTR3BP5(dist=126474)	ACTR3BP5(dist=90613)	Year_26
chr10	39126844	+	chr10	39105356	-	ITX	ACTR3BP5(dist=135473)	ACTR3BP5(dist=113985)	Year_26
chr10	42381064	-	chr10	38775965	+	INV	NONE(dist=NONE)	LOC399744(dist=34884)	Year_14

chr10	42806953	+	chr10	38816311	-	ITX	NONE(dist=NONE)	LOC399744(dist=75230)	Year_10
chr10	59256945	+	chr10	59257945	-	ITX	MIR3924(dist=192626)	MIR3924(dist=193626)	Year_10,Year_23,Year_28
chr10	59257984	-	chr10	59257659	+	ITX	MIR3924(dist=193665)	MIR3924(dist=193340)	Year_10,Year_28
chr10	98511631	+	chr10	58902214	-	ITX	PIK3AP1(dist=31352)	ZWINT(dist=781180)	Year_10
chr11	25076632	+	chr11	25083531	-	ITX	LUZP2	LUZP2	Year_14,Year_26
chr11	57967348	+	chr11	78369024	-	ITX	OR9Q2(dist=8358)	ODZ4	Year_23,Year_26
chr11	66019898	+	chr11	66018698	-	ITX	PACS1(dist=7680)	PACS1(dist=6480)	Year_10,Year_14,Year_21,Year_23,Year_24,Year_26,Year_28
chr11	99690483	+	chr11	99691307	-	ITX	CNTN5	CNTN5	Year_10,Year_14,Year_21,Year_23,Year_24,Year_26,Year_28
chr12	10732133	+	chr12	10732294	-	ITX	KLRC1(dist=124918)	KLRC1(dist=125079)	Year_10,Year_14,Year_21,Year_23,Year_24,Year_26
chr12	125801148	-	chr12	104359630	+	ITX	AACS(dist=173277)	TDG	Year_10,Year_14,Year_21,Year_23,Year_24,Year_26,Year_28
chr12	128391066	+	chr12	128391944	-	ITX	FLJ37505(dist=7882)	FLJ37505(dist=8760)	Year_10,Year_14,Year_21,Year_23,Year_26,Year_28
chr12	71533278	+	chr12	71533752	-	ITX	TSPAN8	TSPAN8	Year_10,Year_21,Year_23,Year_24,Year_26,Year_28
chr12	71533755	-	chr12	71532794	+	ITX	TSPAN8	TSPAN8	Year_10,Year_14,Year_21,Year_23,Year_24,Year_26,Year_28
chr12	78387844	-	chr12	78383889	+	ITX	NAV3	NAV3	Year_10,Year_14,Year_21,Year_23,Year_24,Year_26,Year_28
chr12	85036177	+	chr12	85046603	-	ITX	TMTC2(dist=1508110)	TMTC2(dist=1518536)	Year_10,Year_14,Year_23,Year_24,Year_26,Year_28

chr12	85046490	-	chr12	85040836	+	ITX	TMTC2(dist=1518423)	TMTC2(dist=1512769)	Year_10,Year_21,Year_24,Year_26
chr13	100522672	+	chr13	100522730	-	ITX	CLYBL	CLYBL	Year_10,Year_14,Year_21,Year_23,Year_26,Year_28
chr13	100522728	-	chr13	100522670	+	ITX	CLYBL	CLYBL	Year_23
chr13	100522730	-	chr13	100522670	+	INV	CLYBL	CLYBL	Year_10,Year_14,Year_21,Year_23,Year_26,Year_28
chr13	19922621	-	chr13	25111436	+	ITX	LINC00421(dist=1732)	PARP4(dist=24488)	Year_10
chr13	21790169	+	chr13	25563861	-	ITX	MRP63(dist=36949)	TPTE2P1(dist=21254)	Year_23,Year_28
chr13	23023797	-	chr13	23023711	+	ITX	FGF9(dist=745157)	FGF9(dist=745071)	Year_28
chr13	24269086	+	chr13	25512014	-	ITX	TNFRSF19(dist=18842)	TPTE2P1	Year_10
chr13	43822339	+	chr13	68114187	-	ITX	ENOX1	PCDH9(dist=309719)	Year_24
chr13	48956334	+	chr13	25125777	-	ITX	RB1	PARP4(dist=38829)	Year_10
chr13	82356872	+	chr13	82356963	-	ITX	SPRY2(dist=1441786)	SPRY2(dist=1441877)	Year_10,Year_14,Year_21,Year_23,Year_24,Year_26,Year_28
chr13	82356975	-	chr13	82356877	+	ITX	SPRY2(dist=1441889)	SPRY2(dist=1441791)	Year_10,Year_14,Year_21,Year_23,Year_24,Year_26,Year_28
chr13	99258525	-	chr13	99257406	+	ITX	STK24(dist=29129)	STK24(dist=28010)	Year_14
chr14	63226284	-	chr14	47117868	+	ITX	KCNH5	MIS18BP1(dist=1395263)	Year_14,Year_24,Year_26,Year_28
chr14	65842537	+	chr14	65843135	-	ITX	MIR4708(dist=40636)	MIR4708(dist=41234)	Year_10,Year_14,Year_21,Year_23,Year_24,Year_26,Year_28
chr14	67170331	+	chr14	67171999	-	ITX	GPHN	GPHN	Year_10,Year_14,Year_21,Year_23,Year_24,Year_26,Year_28

chr14	67171710	-	chr14	67170334	+	ITX	GPHN	GPHN	Year_10,Year_14,Year_21,Year_23,Year_24,Year_26,Year_28
chr14	68922455	+	chr14	78559063	-	ITX	RAD51B	ADCK1(dist=158766)	Year_10,Year_23
chr15	28834038	-	chr15	23379103	+	ITX	GOLGA8G(dist=55895)	HERC2P2	Year_10
chr15	71153742	-	chr15	46236788	+	ITX	LARP6(dist=7244)	SQRDL(dist=253309)	Year_24,Year_28
chr16	21594442	+	chr16	22710751	-	ITX	SLC7A5P2(dist=62677)	LOC653786(dist=122565)	Year_14,Year_21,Year_23,Year_24,Year_26,Year_28
chr16	32031446	-	chr16	33692988	+	ITX	ZNF267(dist=102819)	LOC390705(dist=394286)	Year_23
chr16	32296630	+	chr16	32656581	-	ITX	LOC729264(dist=29387)	LOC390705(dist=355279)	Year_23,Year_24,Year_28
chr16	46440101	+	chr16	34185585	-	ITX	NONE(dist=NONE)	LINC00273(dist=223082)	Year_24
chr16	48650883	+	chr16	48651349	-	ITX	N4BP1(dist=6763)	N4BP1(dist=7229)	Year_21,Year_24,Year_28
chr16	48905293	+	chr16	48906227	-	ITX	N4BP1(dist=261173)	N4BP1(dist=262107)	Year_10,Year_14,Year_21,Year_23,Year_24,Year_26,Year_28
chr16	48906236	-	chr16	48905811	+	ITX	N4BP1(dist=262116)	N4BP1(dist=261691)	Year_10,Year_14,Year_21,Year_23,Year_24,Year_26,Year_28
chr16	69762131	+	chr16	69762889	-	ITX	NQO1(dist=1598)	NQO1(dist=2356)	Year_10,Year_14,Year_21,Year_23,Year_24,Year_26,Year_28
chr16	69762896	-	chr16	69761807	+	ITX	NQO1(dist=2363)	NQO1(dist=1274)	Year_10,Year_14,Year_21,Year_23,Year_24,Year_26,Year_28
chr16	74434768	-	chr16	70228732	+	ITX	LOC283922(dist=32615)	CLEC18C(dist=7934)	Year_24

chr17	36350387	+	chr17	36406169	-	ITX	TBC1D3(dist=1721)	LOC440434	Year_10,Year_14,Year_21,Year_23,Year_24,Year_26,Year_28
chr17	36399835	-	chr17	36401099	+	ITX	LOC440434	LOC440434	Year_10
chr17	36401099	-	chr17	36399835	+	ITX	LOC440434	LOC440434	Year_14,Year_21,Year_23,Year_24,Year_26,Year_28
chr17	41401056	-	chr17	41379304	+	ITX	TMEM106A(dist=29467)	TMEM106A(dist=7715)	Year_14,Year_24,Year_26
chr17	48102014	-	chr17	48101918	+	ITX	DLX3(dist=29426)	DLX3(dist=29330)	Year_23
chr17	5594699	+	chr17	5595556	-	ITX	NLRP1(dist=106867)	NLRP1(dist=107724)	Year_10,Year_14,Year_21,Year_23,Year_24,Year_26,Year_28
chr17	5595558	-	chr17	5595283	+	ITX	NLRP1(dist=107726)	NLRP1(dist=107451)	Year_10,Year_21,Year_23,Year_26,Year_28
chr17	58446661	+	chr17	64419953	-	ITX	USP32	PRKCA	Year_10
chr18	11509274	+	chr18	11511469	-	ITX	PIEZO2(dist=360513)	PIEZO2(dist=362708)	Year_10,Year_14,Year_21,Year_23,Year_24,Year_26,Year_28
chr18	11511287	-	chr18	11509264	+	ITX	PIEZO2(dist=362526)	PIEZO2(dist=360503)	Year_10,Year_14,Year_21,Year_24,Year_26,Year_28
chr19	48268139	+	chr19	51088715	-	ITX	GLTSCR2(dist=7816)	LRRC4B(dist=17413)	Year_21,Year_23
chr2	10826038	+	chr2	10827195	-	ITX	NOL10	NOL10	Year_10,Year_14,Year_21,Year_23,Year_24,Year_26,Year_28
chr2	10827022	-	chr2	10826046	+	ITX	NOL10	NOL10	Year_10,Year_14,Year_21,Year_23,Year_24,Year_26,Year_28
chr2	10944768	+	chr2	147020926	-	ITX	PDIA6	DKFZp686O1327(dist=1186635)	Year_14
chr2	116974340	-	chr2	116981887	+	ITX	DPP10(dist=372014)	DPP10(dist=379561)	Year_26

chr2	116978299	+	chr2	116982310	-	ITX	DPP10(dist=375973)	DPP10(dist=379984)	Year_10,Year_14,Year_21,Year_23,Year_24,Year_26,Year_28
chr2	116981887	-	chr2	116974340	+	ITX	DPP10(dist=379561)	DPP10(dist=372014)	Year_10,Year_14,Year_21,Year_23,Year_24,Year_28
chr2	117708120	+	chr2	117708206	-	ITX	DPP10(dist=1105794)	DPP10(dist=1105880)	Year_10,Year_21,Year_23,Year_24,Year_26,Year_28
chr2	123476570	+	chr2	123477379	-	ITX	TSN(dist=951142)	TSN(dist=951951)	Year_10,Year_24,Year_26
chr2	123482506	-	chr2	123476602	+	ITX	TSN(dist=957078)	TSN(dist=951174)	Year_10,Year_14,Year_21,Year_23,Year_24,Year_26,Year_28
chr2	125051756	+	chr2	125053240	-	ITX	CNTNAP5	CNTNAP5	Year_10,Year_14,Year_21,Year_23,Year_24,Year_26,Year_28
chr2	125051760	-	chr2	125052915	+	ITX	CNTNAP5	CNTNAP5	Year_10,Year_14,Year_21,Year_23,Year_26,Year_28
chr2	13183197	+	chr2	192630400	-	ITX	LOC100506474(dist=36059)	OBFC2A(dist=77152)	Year_26
chr2	132579354	-	chr2	132534927	+	ITX	C2orf27B(dist=20120)	C2orf27A(dist=9950)	Year_10,Year_21,Year_23,Year_24,Year_26,Year_28
chr2	132976507	-	chr2	132976036	+	ITX	ANKRD30BL	ANKRD30BL	Year_14
chr2	167547087	-	chr2	155006887	+	ITX	SCN7A(dist=203606)	GALNT13	Year_23,Year_24
chr2	169578126	+	chr2	169578072	-	ITX	CERS6	CERS6	Year_24,Year_26
chr2	182565575	+	chr2	66658991	-	ITX	NEUROD1(dist=20183)	MIR4778(dist=73531)	Year_24
chr2	182793657	+	chr2	181880746	-	ITX	SSFA2	UBE2E3	Year_28
chr2	192630400	+	chr2	13183197	-	ITX	OBFC2A(dist=77152)	LOC100506474(dist=36059)	Year_24
chr2	225292951	+	chr2	225293082	-	ITX	FAM124B(dist=26240)	FAM124B(dist=26371)	Year_10,Year_14,Year_23,Year_24

chr2	225293952	-	chr2	225292981	+	ITX	FAM124B(dist=27241)	FAM124B(dist=26270)	Year_10,Year_14,Year_21,Year_23,Year_24,Year_26,Year_28
chr2	28007149	+	chr2	31559696	-	ITX	RBKS	XDH	Year_21,Year_28
chr2	48830521	+	chr2	231423984	-	ITX	STON1-GTF2A1L	SP100(dist=13667)	Year_10,Year_14,Year_28
chr2	61700753	+	chr2	61703458	-	ITX	USP34(dist=2904)	USP34(dist=5609)	Year_10,Year_14,Year_21,Year_23,Year_24,Year_26,Year_28
chr2	89416655	+	chr2	90249525	-	ITX	MIR4436A(dist=304687)	MIR4436A(dist=1137557)	Year_24
chr20	18591542	+	chr20	18595880	-	ITX	DTD1	DTD1	Year_10,Year_26
chr20	26210907	+	chr20	26211109	-	ITX	LOC284801(dist=21038)	LOC284801(dist=21240)	Year_28
chr20	43305847	+	chr20	43312215	-	ITX	LOC79015(dist=5467)	LOC79015(dist=11835)	Year_10,Year_21,Year_23,Year_24,Year_26,Year_28
chr20	43311909	-	chr20	43308892	+	ITX	LOC79015(dist=11529)	LOC79015(dist=8512)	Year_10,Year_14,Year_21,Year_23,Year_24,Year_26,Year_28
chr21	10793841	-	chr21	30514417	+	ITX	TEKT4P2(dist=825248)	C21orf7	Year_14
chr21	27374158	+	chr21	27374701	-	INV	APP	APP	Year_14,Year_23,Year_28
chr21	27374158	+	chr21	27374706	-	ITX	APP	APP	Year_10,Year_14,Year_21,Year_23,Year_24,Year_26,Year_28
chr21	27374701	-	chr21	27374159	+	ITX	APP	APP	Year_10,Year_14,Year_23,Year_24,Year_26,Year_28
chr21	27374706	-	chr21	27374159	+	INV	APP	APP	Year_10,Year_26,Year_28
chr21	28021726	-	chr21	28020683	+	ITX	CYYR1(dist=76145)	CYYR1(dist=75102)	Year_14,Year_21,Year_24
chr3	158795913	+	chr3	158795956	-	ITX	IQCJ	IQCJ	Year_10,Year_23,Year_24,Year_26,Year_28

chr3	158795917	-	chr3	158795875	+	ITX	IQCJ	IQCJ	Year_10,Year_14,Year_21,Year_23,Year_24,Year_26,Year_28
chr3	187860494	+	chr3	193080602	-	ITX	BCL6(dist=396981)	ATP13A5	Year_10
chr3	43835095	-	chr3	43835992	+	ITX	ABHD5(dist=70878)	ABHD5(dist=71775)	Year_10
chr3	43835648	+	chr3	43835995	-	ITX	ABHD5(dist=71431)	ABHD5(dist=71778)	Year_10,Year_14,Year_21,Year_23,Year_24,Year_26,Year_28
chr3	43835992	-	chr3	43835095	+	ITX	ABHD5(dist=71775)	ABHD5(dist=70878)	Year_14,Year_21,Year_23,Year_24,Year_26,Year_28
chr3	80065102	-	chr3	80064470	+	ITX	ROBO1(dist=248043)	ROBO1(dist=247411)	Year_14
chr3	82204007	-	chr3	82203550	+	ITX	GBE1(dist=393057)	GBE1(dist=392600)	Year_14,Year_28
chr4	113397103	+	chr4	113498208	-	ITX	ALPK1(dist=33339)	C4orf21	Year_23,Year_28
chr4	158107564	+	chr4	148053950	-	ITX	GLRB(dist=14322)	TTC29(dist=186916)	Year_28
chr4	168109163	+	chr4	168115265	-	ITX	SPOCK3	SPOCK3	Year_23,Year_28
chr4	168115506	-	chr4	168115072	+	ITX	SPOCK3	SPOCK3	Year_23,Year_28
chr4	17150902	-	chr4	46613058	+	ITX	LDB2(dist=250478)	GABRA2(dist=221002)	Year_14
chr4	190585811	+	chr4	190613116	-	ITX	HSP90AA4P(dist=189467)	HSP90AA4P(dist=216772)	Year_28
chr4	190585813	+	chr4	190613116	-	ITX	HSP90AA4P(dist=189469)	HSP90AA4P(dist=216772)	Year_23,Year_28
chr4	3985166	+	chr4	9104603	-	ITX	FAM86EP(dist=28018)	LOC650293(dist=152476)	Year_14
chr4	43222817	+	chr4	43227658	-	ITX	GRXCR1(dist=190142)	GRXCR1(dist=194983)	Year_10,Year_14,Year_21,Year_23,Year_24,Year_26,Year_28
chr4	43233218	-	chr4	43227524	+	ITX	GRXCR1(dist=200543)	GRXCR1(dist=194849)	Year_10,Year_14,Year_21,Year_23,Year_24,Year_26,Year_28
chr4	49118556	+	chr4	49657310	-	ITX	CWH43(dist=54461)	CWH43(dist=593215)	Year_21
chr4	68717	+	chr4	49298868	-	ITX	ZNF595	CWH43(dist=234773)	Year_10,Year_14,Year_21,Year_24,Year_26

chr4	68717	+	chr4	49305004	-	ITX	ZNF595	CWH43(dist=240909)	Year_10,Year_14
chr4	88847164	+	chr4	88858699	-	ITX	HSP90AB3P(dist=31997)	HSP90AB3P(dist=43532)	Year_23,Year_28
chr4	88858700	-	chr4	88847164	+	ITX	HSP90AB3P(dist=43533)	HSP90AB3P(dist=31997)	Year_28
chr4	91936102	-	chr4	91933591	+	ITX	FAM190A	FAM190A	Year_23,Year_28
chr5	115346717	+	chr5	115351065	-	ITX	AQPEP	AQPEP	Year_23,Year_24,Year_26,Year_28
chr5	115350905	-	chr5	115346739	+	ITX	AQPEP	AQPEP	Year_14,Year_21,Year_23,Year_24,Year_26,Year_28
chr5	169597697	+	chr5	169598751	-	ITX	FOXI1(dist=60968)	FOXI1(dist=62022)	Year_10,Year_14,Year_21,Year_23,Year_24,Year_26,Year_28
chr5	169598756	-	chr5	169597468	+	ITX	FOXI1(dist=62027)	FOXI1(dist=60739)	Year_10,Year_14,Year_21,Year_23,Year_24,Year_26,Year_28
chr5	176578165	+	chr5	176577539	-	ITX	NSD1	NSD1	Year_23,Year_26
chr5	28932651	+	chr5	28935059	-	ITX	LOC729862(dist=5231)	LOC729862(dist=7639)	Year_14,Year_21,Year_23,Year_24,Year_26,Year_28
chr5	28934877	-	chr5	28932856	+	ITX	LOC729862(dist=7457)	LOC729862(dist=5436)	Year_10
chr5	79046316	+	chr5	79049540	-	ITX	CMYA5	CMYA5	Year_10,Year_14,Year_21,Year_24,Year_26,Year_28
chr5	79051661	-	chr5	79046132	+	ITX	CMYA5	CMYA5	Year_10,Year_14,Year_21,Year_23,Year_24,Year_26,Year_28
chr6	112950566	+	chr6	114091216	-	ITX	RFPL4B(dist=278068)	RFPL4B(dist=1418718)	Year_23,Year_26
chr6	122979993	-	chr6	136735090	+	ITX	PKIB	MAP7	Year_28
chr6	131884961	+	chr6	131884865	-	ITX	AKAP7(dist=280286)	AKAP7(dist=280190)	Year_14,Year_28
chr6	136807000	+	chr6	74504855	-	ITX	MAP7	CD109	Year_10,Year_28

chr6	22815019	+	chr6	18626780	-	ITX	HDGFL1(dist=244269)	MIR548A1(dist=54669)	Year_14,Year_28
chr6	25647709	+	chr6	25647927	-	ITX	LRRC16A(dist=26951)	LRRC16A(dist=27169)	Year_10,Year_14,Year_21,Year_23,Year_24,Year_26,Year_28
chr6	34699922	+	chr6	34701124	-	ITX	C6orf106(dist=35297)	C6orf106(dist=36499)	Year_10,Year_21,Year_24
chr6	78843172	-	chr6	144633296	+	ITX	HTR1B(dist=670052)	UTRN	Year_28
chr6	82816414	-	chr6	113856165	+	ITX	FAM46A(dist=353986)	RFPL4B(dist=1183667)	Year_10
chr6	84207449	+	chr6	84610835	-	ITX	ME1(dist=66511)	CYB5R4	Year_21,Year_28
chr6	89923634	+	chr6	89923949	-	ITX	GABRR1	GABRR1	Year_10,Year_14,Year_21,Year_24,Year_26,Year_28
chr6	89923949	+	chr6	89923634	-	ITX	GABRR1	GABRR1	Year_23
chr7	24937247	+	chr7	91751552	-	ITX	OSBPL3	CYP51A1	Year_26,Year_28
chr7	31591996	-	chr7	31586772	+	ITX	CCDC129	CCDC129	Year_10,Year_14,Year_21,Year_23,Year_24,Year_26,Year_28
chr7	40879375	+	chr7	40880470	-	ITX	C7orf10	C7orf10	Year_10,Year_21,Year_23,Year_24,Year_26,Year_28
chr7	40880470	-	chr7	40879376	+	INV	C7orf10	C7orf10	Year_10,Year_14,Year_26
chr7	40880470	-	chr7	40879376	+	ITX	C7orf10	C7orf10	Year_14,Year_21,Year_24,Year_26,Year_28
chr7	45884323	+	chr7	35968528	-	ITX	SEPT7P2(dist=75706)	SEPT7(dist=21813)	Year_28
chr7	70420969	+	chr7	70438887	-	ITX	AUTS2(dist=163084)	AUTS2(dist=181002)	Year_10,Year_14,Year_23,Year_24,Year_26,Year_28
chr7	70438880	-	chr7	70426185	+	ITX	AUTS2(dist=180995)	AUTS2(dist=168300)	Year_10,Year_14,Year_21,Year_23,Year_24,Year_26,Year_28
chr7	70438887	+	chr7	70420969	-	ITX	AUTS2(dist=181002)	AUTS2(dist=163084)	Year_21
chr7	75664293	+	chr7	75666108	-	ITX	STYXL1	STYXL1	Year_10,Year_21,Year_23,Year_24,Year_26,Year_28

chr7	75666028	-	chr7	75667808	+	ITX	STYXL1	STYXL1	Year_24
chr7	75667808	-	chr7	75666028	+	ITX	STYXL1	STYXL1	Year_10,Year_14,Year_23,Year_26,Year_28
chr7	7583516	+	chr7	10708427	-	ITX	COL28A1(dist=8056)	PER4(dist=1032980)	Year_10
chr7	78662180	-	chr7	90022145	+	ITX	MAGI2	GTPBP10(dist=1376)	Year_26
chr8	108569293	+	chr8	108569363	-	ITX	ANGPT1(dist=59039)	ANGPT1(dist=59109)	Year_10,Year_14,Year_21,Year_24,Year_26,Year_28
chr8	118873738	+	chr8	118875337	-	ITX	EXT1	EXT1	Year_10,Year_14,Year_21,Year_23,Year_24,Year_26,Year_28
chr8	118875594	-	chr8	118875219	+	ITX	EXT1	EXT1	Year_10,Year_14,Year_21,Year_23,Year_24,Year_26,Year_28
chr8	96467779	-	chr8	117763168	+	ITX	LOC100616530	EIF3H	Year_21
chr9	107816979	+	chr9	107817341	-	ITX	ABCA1(dist=126452)	ABCA1(dist=126814)	Year_10,Year_14,Year_21,Year_23,Year_24,Year_26,Year_28
chr9	107817348	-	chr9	107816637	+	ITX	ABCA1(dist=126821)	ABCA1(dist=126110)	Year_10,Year_14,Year_21,Year_23,Year_24,Year_28
chr9	66462154	+	chr9	66474387	-	ITX	FAM75A5(dist=952544)	FAM75A5(dist=964777)	Year_10,Year_14,Year_21,Year_24,Year_26,Year_28
chr9	66478619	-	chr9	66469908	+	ITX	FAM75A5(dist=969009)	FAM75A5(dist=960298)	Year_10,Year_14,Year_21,Year_23,Year_26,Year_28
chr9	67711289	+	chr9	45080739	-	ITX	AQP7P1(dist=421797)	FAM27C(dist=89247)	Year_10,Year_14,Year_23
chr9	89154557	+	chr9	89155930	-	ITX	ZCCHC6(dist=185155)	ZCCHC6(dist=186528)	Year_10,Year_14,Year_21,Year_23,Year_24,Year_26,Year_28
chr9	89155947	-	chr9	89155843	+	ITX	ZCCHC6(dist=186545)	ZCCHC6(dist=186441)	Year_23,Year_26,Year_28
chrX	114544514	-	chrX	114216669	+	ITX	LUZP4(dist=2393)	HTR2C(dist=72045)	Year_14,Year_23,Year_26

chrX	131005307	+	chrX	131007921	-	ITX	LOC286467(dist=40636)	LOC286467(dist=43250)	Year_10,Year_21,Year_23,Year_26,Year_28
chrX	131008631	-	chrX	131007721	+	ITX	LOC286467(dist=43960)	LOC286467(dist=43050)	Year_10,Year_14,Year_21,Year_23,Year_24,Year_26,Year_28
chrX	29269149	-	chrX	29270197	+	ITX	IL1RAPL1	IL1RAPL1	Year_23,Year_28
chrX	5729126	+	chrX	5730792	-	ITX	LOC389906(dist=1967191)	LOC389906(dist=1968857)	Year_10,Year_14,Year_21,Year_23,Year_24,Year_26,Year_28
chrX	5730607	-	chrX	5729125	+	ITX	LOC389906(dist=1968672)	LOC389906(dist=1967190)	Year_10,Year_14,Year_21,Year_23,Year_24,Year_26,Year_28
chrX	6137049	+	chrX	6138384	-	ITX	NLGN4X	NLGN4X	Year_10,Year_14,Year_21,Year_23,Year_24,Year_26,Year_28
chrX	78921889	+	chrX	78923365	-	ITX	ITM2A(dist=298840)	ITM2A(dist=300316)	Year_10,Year_14,Year_21,Year_24,Year_26,Year_28
chrX	78926077	-	chrX	78923226	+	ITX	ITM2A(dist=303028)	ITM2A(dist=300177)	Year_10,Year_14,Year_21,Year_23,Year_24,Year_26,Year_28
